# Procyanidin B3 and Its Derivatives Alleviate Neuronal Injury by Targeting G3BP1 for Ischemic Stroke Therapy

**DOI:** 10.1002/advs.202509781

**Published:** 2025-09-26

**Authors:** Heyanhao Zhang, Yuyu Zhang, Yibo Chen, Wen Zhong, Subei Tan, Jinghuan Wang, Huanren Yan, Ning Yan, Mengjia Lin, Xinhua Liu, Jun Chang

**Affiliations:** ^1^ Phenome Research Center of TCM Department of Traditional Chinese Medicine Shanghai Pudong Hospital Pharmacophenomics Laboratory Human Phenome Institute Fudan University Shanghai 201203 China

**Keywords:** chemical proteomics, G3BP1, ischemic stroke, procyanidin B3, structural modification

## Abstract

Ischemic stroke (IS) is a leading cause of mortality and disability worldwide, but effective therapeutic options are limited. In this study, a chemical proteomic strategy is employed using the active compound procyanidin B3 (PB3) as a chemical probe to identify the therapeutic targets for IS. It is discovered that the Ras GTPase‐activating protein SH3 domain‐binding protein 1 (G3BP1) is a key target of PB3, which exerts a neuroprotective effect by inhibiting the degradation of stress granules and reducing apoptosis. Based on this finding, 14 PB3 derivatives are designed and synthesized, among which compound **6c** exhibited potent neuroprotective activity and favorable blood‐brain barrier permeability. This study not only establishes G3BP1 as a promising therapeutic target for IS but also highlights the potential of PB3 and its derivatives for the development of IS therapeutic agents.

## Graphical abstract

Ischemic stroke is a serious disease with high rates of mortality and disability, but there is a lack of novel therapeutic targets and agents for it. Now it is shown that Ras GTPase‐activating protein SH3 domain‐binding protein 1 (G3BP1) has the potential to serve as a therapeutic target for ischemic stroke. Procyanidin B3 and its derivatives can be potentially used for developing anti‐ischemic stroke agents by targeting G3BP1.

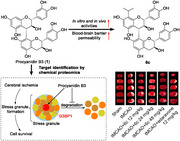

## 1 Introduction

Stroke is an important factor leading to disability and one of the leading causes of death worldwide, posing a serious threat to human health.^[^ ^1^ ^]^ Ischemic stroke (IS) accounts for about 80% of stroke cases and is the most common type of stroke.^[^ ^2^ ^]^ At present, the clinical treatment strategies for IS can achieve benefits from thrombolysis,^[^ ^3^ ^]^ antiplatelet,^[^ ^4^ ^]^ microcirculation improvement,^[^ ^5^ ^]^ and neuroprotection,^[^ ^6^ ^]^ but there is still a lack of specific targeting agents.^[^ ^7^ ^]^ Timely diagnosis ^[^ ^8^ ^]^ and effective intervention ^[^ ^9^ ^]^ of IS are essential for improving prognosis. Thus, exploring new therapeutic targets for IS and developing novel agents for the treatment of IS are of great practical significance and value. Our research group has been actively engaged in advancing this field through systematic investigations of IS therapeutic targets^[^ ^10^ ^]^ and the development of potential treatment agents.^[^ ^11^ ^]^

Stress granules (SGs) are dynamic, reversible cytoplasmic assemblies that form in eukaryotic cells in response to stress, composed of mRNAs, mRNA‐binding proteins, 40S ribosomal subunits, and translation initiation factors.^[^ ^12^ ^]^ In cerebral ischemia, SGs rapidly assemble upon stress onset and subsequently disassemble upon reperfusion.^[^ ^13^ ^]^ During the formation and degradation of SG, Ras GTPase‐activating protein SH3 domain‐binding protein 1 (G3BP1) plays a crucial role as the core of the protein‐protein interaction network of SG.^[^ ^14^, ^15^ ^]^ Accordingly, it is meaningful to investigate how G3BP1 and SG function in ischemic injury. Reducing ischemia‐induced apoptosis in brain tissue is essential to repairing ischemic injury.^[^ ^16^, ^17^ ^]^ While several studies have demonstrated the anti‐apoptotic effects of SGs in ischemia,^[^ ^18^, ^19^ ^]^ the specific role of G3BP1 as the core regulator of SG assembly remains poorly characterized. Targeting G3BP1 may alleviate ischemia‐induced apoptosis by regulating SG dynamics, making it a viable therapeutic strategy for IS.

**Figure 1:**
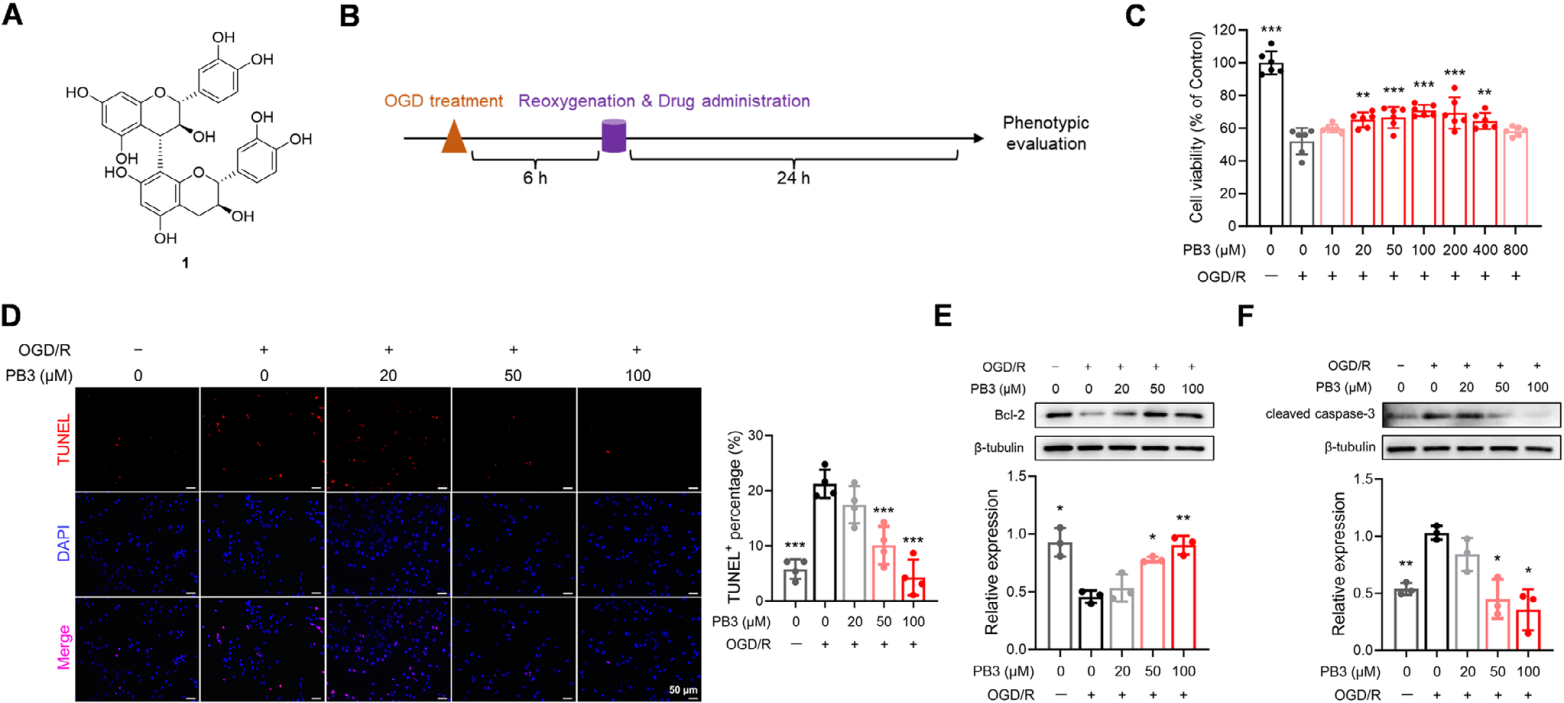
PB3 decreases the HT22 cell mortality and apoptosis level under OGD/R injury. A) Structure of PB3 (1). B) The cell experimental paradigm. C) Neuroprotective effect of PB3 at concentrations ranging from 10 to 800 µm on OGD/R‐treated HT22 cells. Data are expressed as mean ± S.D., ^**^ *p* < 0.01, ^***^ *p* < 0.001 versus PB3 0 µm, OGD/R+, one‐way ANOVA, Dunnett's multiple comparison test (*n* = 6). D) Rate of TUNEL‐positive cells in different experimental groups. Data are expressed as mean ± S.D., ^***^ *p* < 0.001 versus PB3 0 µm, OGD/R+, one‐way ANOVA, Dunnett's multiple comparison test (*n* = 4). Scale bar, 50 µm. E) Immunoblots showing the expression of Bcl‐2 in different experimental groups. Data are expressed as mean ± S.D., ^*^ *p* < 0.05, ^**^ *p* < 0.01 versus PB3 0 µm, OGD/R+, one‐way ANOVA, Dunnett's multiple comparison test (*n* = 3). F) Immunoblots showing the expression of cleaved caspase‐3 in different experimental groups. Data are expressed as mean ± S.D., ^*^ *p* < 0.05, ^**^ *p* < 0.01 versus PB3 0 µm, OGD/R+, one‐way ANOVA, Dunnett's multiple comparison test (*n* = 3).

Oligomeric proanthocyanidins are widely distributed in nature and have garnered significant research interest due to their extensive biological activities.^[^ ^20^ ^]^ Among these compounds, procyanidin B3 (PB3, **1**, **Figure** **1**A) has demonstrated notable neuroprotective properties, including protection of PC12 cells and preservation of dopaminergic neurons in zebrafish models of Parkinson's disease.^[^ ^21^ ^]^ Moreover, PB3 has shown efficacy in mitigating amyloid beta protein‐induced neurotoxicity,^[^ ^22^ ^]^ highlighting its potential as a therapeutic agent for central nervous system diseases. Our previous work revealed that PB3 ameliorates the pathology features of IS, suggesting its potential therapeutic effect on IS.^[^ ^23^ ^]^ These findings position PB3 as a promising chemical probe for investigating the therapeutic targets of IS. In addition, the clinical translation of PB3 may be constrained by limited blood‐brain barrier (BBB) permeability, a likely consequence of its high hydroxyl group content. The structural modifications of PB3 could optimize its pharmacodynamic profile and pharmacokinetic properties.

In this study, we implemented a chemical proteomic strategy to investigate the target of the active compound PB3 in the treatment of IS and identified G3BP1 as one of the key targets of PB3. Liquid chromatography‐tandem mass spectrometry (LC‐MS/MS) analysis coupled with molecular docking predicted the binding of PB3 to the NTF2L domain of G3BP1. Mechanistic studies revealed that by targeting G3BP1, PB3 could inhibit the degradation of SGs, alleviating neuronal injury triggered by oxygen‐glucose deprivation/reoxygenation (OGD/R). To optimize therapeutic potential, we structurally modified PB3 to enhance BBB permeability through increased lipophilicity, resulting in improved efficacy against IS. These findings not only establish G3BP1 as a promising therapeutic target for IS intervention but also provide a new chemical template for the future design and development of IS therapeutic agents.

## 2 Results and Discussion

### 2.1 PB3 Protects the Neuron in Both In Vitro and In Vivo Models of IS

To evaluate the therapeutic potential of PB3, an in vitro ischemia‐reperfusion model was established in which HT22 cells were exposed to OGD/R (Figure 1B). After 6 h of OGD, cells were reoxygenated and treated with different concentrations of PB3. 24 h later, cell viability was assessed by the CCK8 experiment.^[^ ^24^ ^]^ The results showed that PB3 could significantly improve cell viability rate at the concentration of 20 to 400 µm, indicating that PB3 has neuroprotective effects (Figure 1C). Terminal deoxynucleotidyl transferase‐mediated dUTP nick‐end labeling (TUNEL) staining and immunoblotting were utilized to investigate the apoptosis level of HT22 cells in different groups. According to the TUNEL staining results, PB3 dose‐dependently reduced the rate of TUNEL‐positive cells (Figure 1D). Immunoblotting assays were used to detect the protein expression levels of the apoptotic indicators B‐cell lymphoma‐2 (Bcl‐2) and cleaved caspase‐3.^[^ ^25^ ^]^ It was found that compared with the model group, the expression of Bcl‐2 in PB3 groups (50 and 100 µm) was significantly increased (Figure 1E), and the expression of cleaved caspase‐3 was significantly decreased (Figure 1F), which was consistent with the results of the TUNEL assay. These results suggest that PB3 has neuroprotective and anti‐apoptotic effects in vitro and can protect neuron from OGD/R injury.

Next, we constructed a transient middle cerebral artery occlusion (tMCAO) model in mice for in vivo studies. 1.5 h of MCAO followed by 24 h of reperfusion was selected to produce profound and progressive infarcted areas of brain damage (Figure S1A, Supporting Information). The neurological deficit score assessment at 24 h after reperfusion revealed that the treatment with 48 mg kg^−1^ of PB3 by intraperitoneal (*i.p*.) injection improved neurological deficits in tMCAO mice (Figure S1B, Supporting Information). And the infarct volume of tMCAO mice significantly reduced after the administration of PB3 (Figure S1C, Supporting Information). The TUNEL staining results of frozen brain sections showed that the apoptosis level in the PB3 group was significantly reduced compared to the model group (Figure S1D, Supporting Information). The expression levels of the apoptotic indicators in the ischemic cortex of tMCAO mice were analyzed via immunoblotting. In the PB3 group, the expression level of Bcl‐2 was increased (Figure S1E, Supporting Information), and the expression level of cleaved caspase‐3 was decreased (Figure S1F, Supporting Information) compared to the model group. These data indicated that PB3 reduces neuronal damage and apoptosis after tMCAO. Taken together, these results reveal that PB3 could protect the neuron and demonstrate anti‐ischemic stroke effects in both in vitro and in vivo models.

### 2.2 G3BP1 Serves as a Cellular Target of Anti‐Ischemic Stroke Small‐Molecule PB3

To identify the target of the active compound PB3, we performed chemical proteomic analysis based on the compound‐centric chemical proteomic strategy.^[^ ^26^ ^]^ First, we prepared a photoaffinity probe through condensation reactions from the NHS‐activated agarose beads (Figure S2A, Supporting Information), and its preparation was verified by Fourier transform infrared spectroscopy (Figure S2B, Supporting Information). The photoaffinity probe was divided into two parts; one part was quenched by ultraviolet light to gain a control probe, and the other part was ultraviolet‐crosslinked with PB3 to gain a PB3 probe (Figure S2C, Supporting Information). The successful preparation of the PB3 probe was confirmed by quantification of PB3 in eluent via high‐performance liquid chromatography (Figure S2D, Supporting Information) and ferric chloride staining (Figure S2E, Supporting Information). Through scanning electron microscope analysis, most of the PB3 probes were found in intact form (Figure S2F, Supporting Information).

**Figure 2:**
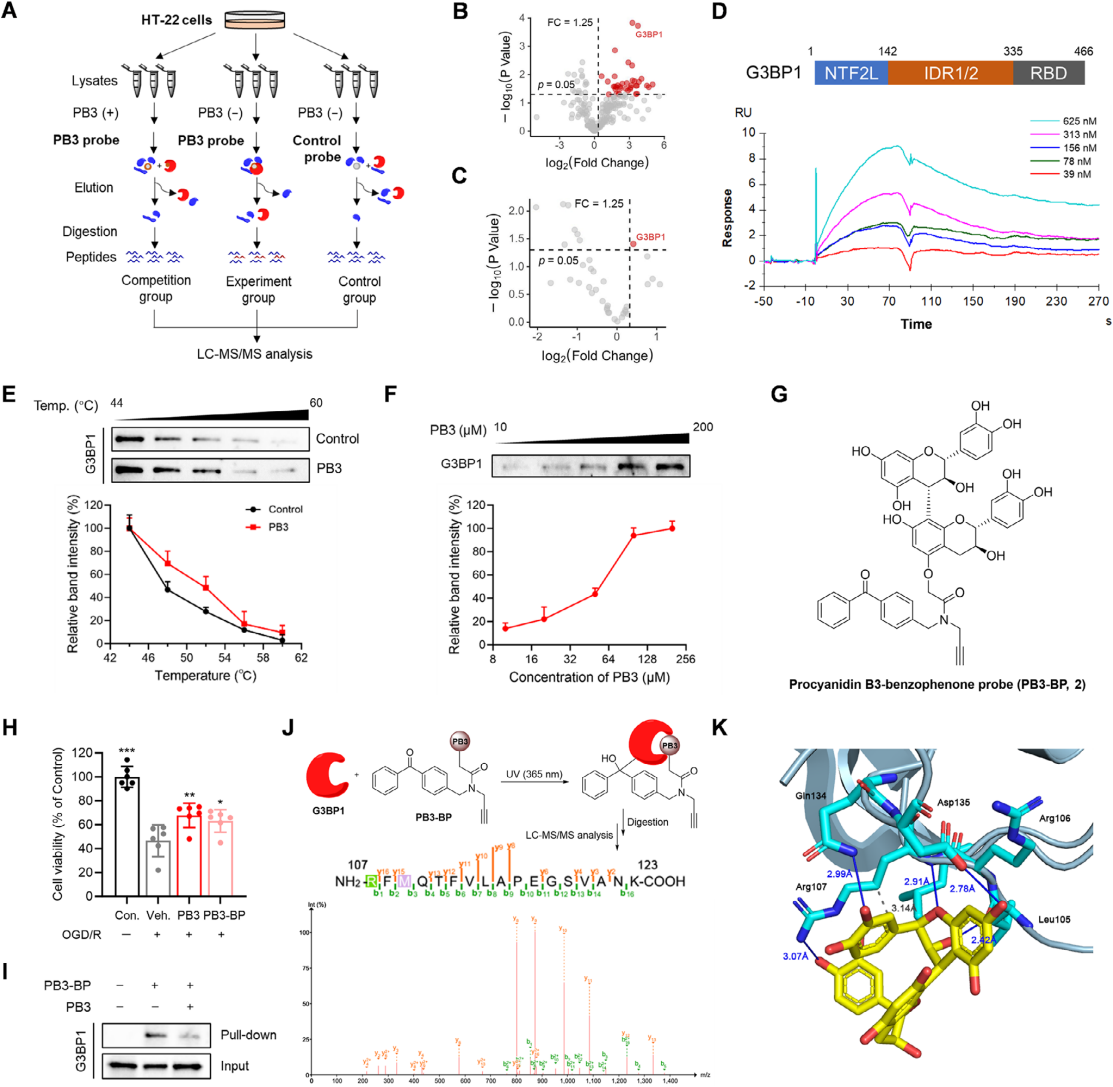
G3BP1 serves as a target of PB3. A) The workflow of chemical proteomics for target identification of PB3. B) Volcano plot of differential analysis between the experiment group and the control group. Red dots represent PB3‐specifically bound proteins. C) In the PB3‐specifically bound proteins, volcano plot of differential analysis between the experiment group and the competition group. D) SPR analysis of PB3 binding to G3BP1. E) 100 µm PB3 improved the thermal stability of G3BP1. Data are expressed as mean ± S.D. (*n* = 3). F) The thermal stability of G3BP1 increases with the increase of PB3 concentration at 52 °C. Data are expressed as mean ± S.D. (*n* = 3). G) Structure of PB3‐BP (2). H) Neuroprotective effect of PB3 and PB3‐BP at 100 µm on OGD/R‐treated HT22 cells. Data are expressed as mean ± S.D., ^*^ *p* < 0.05, ^**^ *p* < 0.01, ^***^ *p* < 0.001 versus Veh., one‐way ANOVA, Dunnett's multiple comparison test (*n* = 6). I) Pull‐down analysis of PB3 binding to G3BP1. J) LC‐MS/MS analysis of the PB3‐BP‐bound fragment derived from G3BP1. The resulting b‐ions (N‐terminal) and y‐ions (C‐terminal) of the modified peptide RFMQTFFVLAPEGSVANK have been labeled (R107 residue, green). M109 is marked in purple to indicate methionine oxidation. K) Docking simulation of PB3 with the NTF2L domain of G3BP1. Hydrogen bonds are labeled as blue lines, and hydrophobic interactions are labeled as grey dashed lines.

Then, HT22 cell lysates were used for target enrichment. Three parallel experiments were carried out. In the experiment group, the PB3 probe was incubated with cell lysates, and the PB3 binding proteins were enriched. In the competition group, the cell lysates were preincubated with free PB3 and then incubated with the PB3 probe. The proteins that could be competed away by free PB3 were considered as potential targets.^[^ ^27^ ^]^ In the control group, the control probe was used to identify non‐specifically bound proteins. After incubation, the enriched proteins in each group were eluted, digested, and analyzed by LC‐MS/MS (**Figure** **2**A). A total of 706 proteins were identified, and 37 proteins were considered as PB3‐specifically bound proteins by comparing the experiment group and the control group (Figure 2B). The list of PB3‐specifically bound proteins is shown in Table S1 (Supporting Information). In the specifically bound proteins, further comparison was made between the experiment group and the competition group, and we found that G3BP1 was competed away by free PB3 (Figure 2C). Thus, we speculated that G3BP1 may serve as a cellular target of PB3 to protect neurons from ischemic damage. Surface plasmon resonance (SPR) analysis indicated that PB3 directly bound to G3BP1 (*K*_D_ = 44 nm, Figure 2D), and a cellular thermal shift assay ^[^ ^28^ ^]^ revealed that PB3 enhanced the thermal stability of G3BP1 (Figure 2E,F). These results demonstrate that PB3 could bind to G3BP1. Immunoblotting analysis confirmed that this interaction did not perturb the protein level of G3BP1 (Figure S3, Supporting Information).

Furthermore, a small‐molecule procyanidin B3‐benzophenone probe (PB3‐BP, **2**, Figure 2G) was synthesized based on a non‐protective condensation strategy (Scheme S1, Supporting Information).^[^ ^29^ ^]^ The stability of compound **2** is poor, and its structure is determined by 2D‐NMR after acetylation (Figure S4, Supporting Information). PB3‐BP retained the neuroprotective activity of PB3 and can be used in activity‐based protein profiling (Figure 2H). We observed that G3BP1 was pulled down by PB3‐BP, which was obviously blocked by free PB3 for competition (Figure 2I). To identify the specific targeting sites of PB3 on G3BP1, the ultraviolet‐crosslinked complex of PB3‐BP/G3BP1 was digested with trypsin and subjected to LC‐MS/MS analysis.^[^ ^30^ ^]^ A 697.1948 Da increase in molecular weight, corresponding to the photoactivated PB3‐BP fragment followed by the retro‐Diels‐Alder reaction (Scheme S2, Supporting Information),^[^ ^31^, ^32^ ^]^ was detected to bind at R107 of the peptide RFMQTFVLAPEGSVANK (Figure 2J), suggesting that the possible binding sites of PB3 may fall into the NTF2L domain of G3BP1. To predict the interactions between PB3 and G3BP1, we docked PB3 into the NTF2L domain of G3BP1 (PDB code: 8V1L) ^[^ ^33^ ^]^ around Arg107, and the results showed that PB3 interacts with residues such as Leu105, Arg107, Gln134, and Asp135 (Figure 2K). To evaluate the affinity between the wild type and four mutants (L105A, R107A, Q134A, D135A) ^[^ ^34^ ^]^ of the G3BP1 NTF2L domain and PB3, molecular dynamics simulations were performed to analyze each complex system based on docking results.^[^ ^35^ ^]^ MMGBSA analysis ^[^ ^36^, ^37^ ^]^ revealed that compared with the wild‐type complex, all mutants have higher binding free energy, suggesting the importance of these key residues for PB3 binding to G3BP1 (Table S2, Supporting Information).

**Figure 3:**
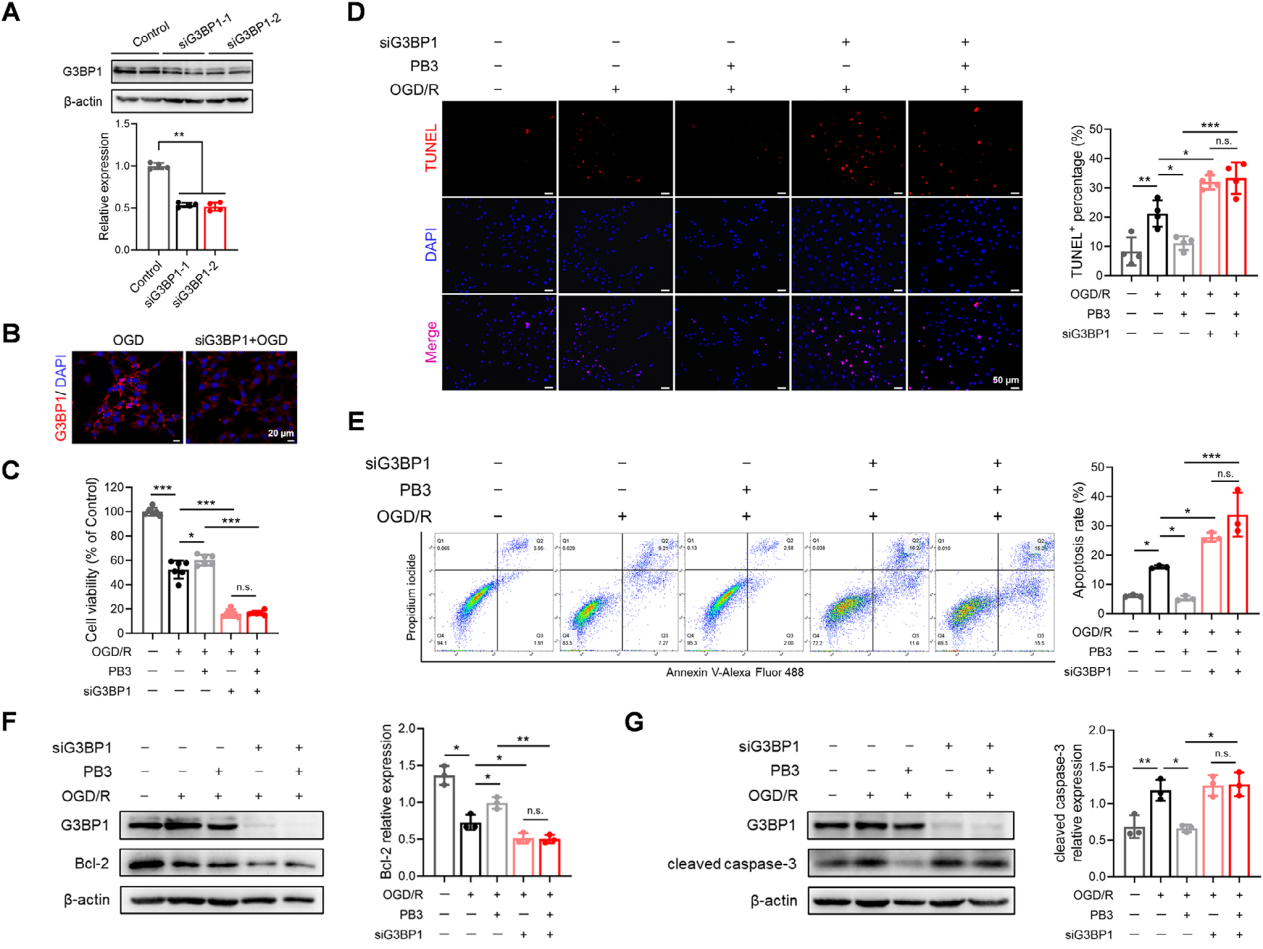
Effects of G3BP1 knockdown on the activity of PB3. A) The inhibition efficiency of siRNA was verified by immunoblotting. Data are expressed as mean ± S.D., ^**^ *p* < 0.01, one‐way ANOVA, Tukey's post hoc test (*n* = 4). B) Immunofluorescence staining of siG3BP1‐treated HT22 cells exposed to OGD. Scale bar, 20 µm. C) Knockdown of G3BP1 inhibited the neuroprotective effects of PB3 (50 µm). Data are expressed as mean ± S.D., ^*^ *p* < 0.05, ^***^ *p* < 0.001, n.s. = no significance, one‐way ANOVA, Tukey's post hoc test (*n* = 6). D–G) Knockdown of G3BP1 inhibited the anti‐apoptotic effects of PB3 (50 µm) detected by TUNEL staining D), flow‐cytometric analysis E), and immunoblotting analysis of apoptosis‐related protein levels F,G). Data are expressed as mean ± S.D., ^*^ *p* < 0.05, ^**^ *p* < 0.01, ^***^ *p* < 0.001, n.s. = no significance, one‐way ANOVA, Tukey's post hoc test (*n* = 3–4). Scale bar, 50 µm.

To test the potential role of G3BP1 in the anti‐ischemic stroke effect of PB3, G3BP1 was knocked down by small interfering RNA (siRNA) transfection in HT22 cells (**Figure** **3**A,B). The results of the cell viability assay showed that G3BP1 knockdown inhibited the neuroprotective effects of PB3 (Figure 3C). The TUNEL staining (Figure 3D) demonstrated that the rate of TUNEL‐positive cells was not significantly changed after the administration of PB3 in G3BP1 knockdown HT22 cells. In addition, the results of flow‐cytometric analysis (Figure 3E) and immunoblotting (Figure 3F,G) demonstrated that knocking down G3BP1 significantly inhibited the anti‐apoptotic effects of PB3. Based on the above results, it is suggested that G3BP1 knockdown inhibited the neuroprotective and anti‐apoptotic effects of PB3, and PB3 possibly protects neurons from ischemic damage through G3BP1.

### 2.3 PB3 Mediates SG Upregulation in Both In Vitro and In Vivo Models of IS

**Figure 4:**
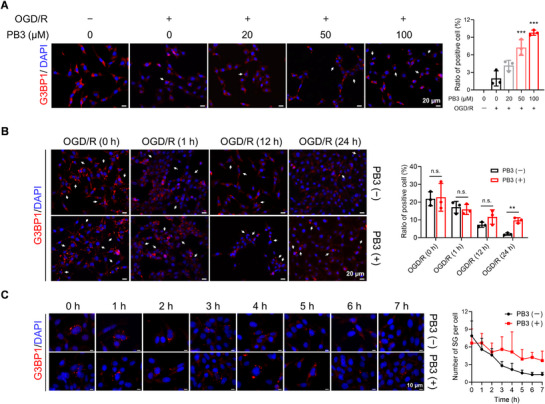
PB3 enhanced SG level in HT22 cells under OGD/R injury. A) Immunofluorescence staining was performed to detect SG level after PB3 administration at different concentrations in HT22 cells under OGD/R injury. Data are expressed as mean ± S.D., ^***^ *p* < 0.001 versus PB3 0 µm, OGD/R+, one‐way ANOVA, Dunnett's multiple comparison test (*n* = 3). Scale bar, 20 µm. B) The effect of PB3 administration (100 µm) on the ratio of SG‐positive cells at 0, 1, 12, and 24 h after reoxygenation. Data are expressed as mean ± S.D., ^**^ *p* < 0.01, n.s. = no significance, Student's *t*‐test (*n* = 3). Scale bar, 20 µm. C) The effect of PB3 administration (50 µm) on the number of SG per cell at 0–7 h after reoxygenation. Data are expressed as mean ± S.D. (*n* ≥ 6). Scale bar, 10 µm.

As a central node of the network of SG protein‐protein interactions, G3BP1 is crucial to the formation and degradation of SG. To investigate whether PB3 regulates SGs after targeting G3BP1, HT22 cells were treated with OGD. After 6 h of OGD, different concentrations of PB3 were administered. 24 h later, we performed the immunofluorescence staining of G3BP1^[^ ^38^ ^]^ to detect the SG formation in HT22 cells (**Figure** **4**A). The results showed that PB3 can enhance SG levels in a dose‐dependent manner. As noted in the preceding text, PB3 could improve OGD/R‐induced apoptosis (Figure 1D–F). These findings indicate that, under PB3 intervention, there is a negative correlation between the apoptosis level and the SG level, and this correlation is consistent with literature reports.^[^ ^39^, ^40^ ^]^ Previous studies have shown that SG formation induced by oxidative stress could inhibit apoptosis.^[^ ^25^ ^]^ Therefore, the role of PB3 in regulating SG level may be critical for inhibiting neuronal apoptosis and alleviating injury.

In order to further explore whether PB3 promotes SG formation or inhibits its degradation, the effect of PB3 administration on the ratio of SG‐positive cells and the number of SG per cell was observed. It was found that after 24 h of reoxygenation, the ratio of SG‐positive cells significantly increased in the group treated with PB3 (Figure 4B). However, shortly after PB3 administration (1 h), the ratio of SG‐positive cells did not change significantly, indicating that PB3 can inhibit SG degradation. The assessment of the number of SG per cell confirmed the effect of PB3 on SG dynamics (Figure 4C). The expression of key factors regulating SG dynamics was detected by reverse transcription‐quantitative polymerase chain reaction (RT‐qPCR) after 6 h of reperfusion following OGD. The expression of eIF2α phosphatases (GADD34 and PPP1R15B/CReP) ^[^ ^41^ ^]^ in HT22 cells of the PB3 treatment group (50 µm of PB3) was significantly lower, while the expression of two eIF2α kinases (PERK and GCN2) ^[^ ^42^ ^]^ was significantly higher with PB3 administration (Figure S5, Supporting Information). Phosphorylation of eIF2α is directly related to the dynamics of SGs,^[^ ^43^ ^]^ so the effect of PB3 on the levels of eIF2α phosphorylation‐related factors during the SG degradation process may stabilize SG, which provides evidence at the molecular level that PB3 regulates SG dynamics. On the other hand, it has been reported that *Cdk6*, *Peg3*, and *Dync1h1* mRNA can be enriched from SGs by isolating G3BP1 cores.^[^ ^44^ ^]^ Based on this, we isolated RNA that binds to G3BP1 after 6 h of reperfusion following OGD via RNA immunoprecipitation (RIP) assay, and the RT‐qPCR analysis was then applied to detect these G3BP1‐bound mRNAs. The results showed that the level of G3BP1‐bound mRNA was higher in the PB3 treatment group (50 µm of PB3), indicating that PB3 may enhance the RNA‐binding ability of G3BP1 and stabilize SG (Figure S6, Supporting Information), providing additional evidence for PB3 in inhibiting SG degradation.

Moreover, PB3 treatment (48 mg kg^−1^, *i.p*.) significantly enhanced SG levels in the ischemic cortex of tMCAO mice (Figure S7, Supporting Information), which aligns with the previous finding that PB3 administration reduces neuronal apoptosis after tMCAO (Figure S1D–F, Supporting Information). This result parallels that observed in the OGD/R model mentioned above and preliminarily explains the anti‐ischemic stroke effect mechanism of PB3 in vivo.

### 2.4 Design, Synthesis, and In Vitro Neuroprotective Effects of PB3 Derivatives

PB3 contains many hydroxyl groups, and its application may be limited by its potentially poor BBB permeability. In CNS drug research, physicochemical properties such as lipophilicity are critical for the BBB permeability.^[^ ^45^, ^46^ ^]^ As a result, it is hypothesized that enhancing the lipophilicity of PB3 can improve its BBB crossing capacity, allowing it to better act on ischemic brain tissue and thereby enhance its therapeutic effect on IS.

**Scheme 1:**
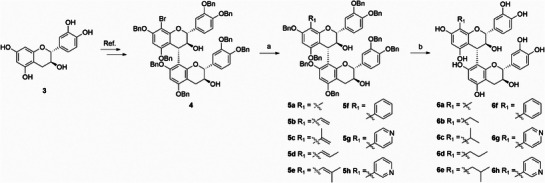
Synthesis of C8‐substituted derivatives of PB3. Reagents and conditions: a) boron reagent, K_2_CO_3_, Pd(PPh_3_)_4_, 1,4‐dioxane/H_2_O (or 1,4‐dioxane/acetonitrile/H_2_O), reflux, 16–42 h, 47%‐96%; b) 20% Pd(OH)_2_/C, H_2_, MeOH, room temperature, 2–6 h, 8%–63%.

**Scheme 2:**
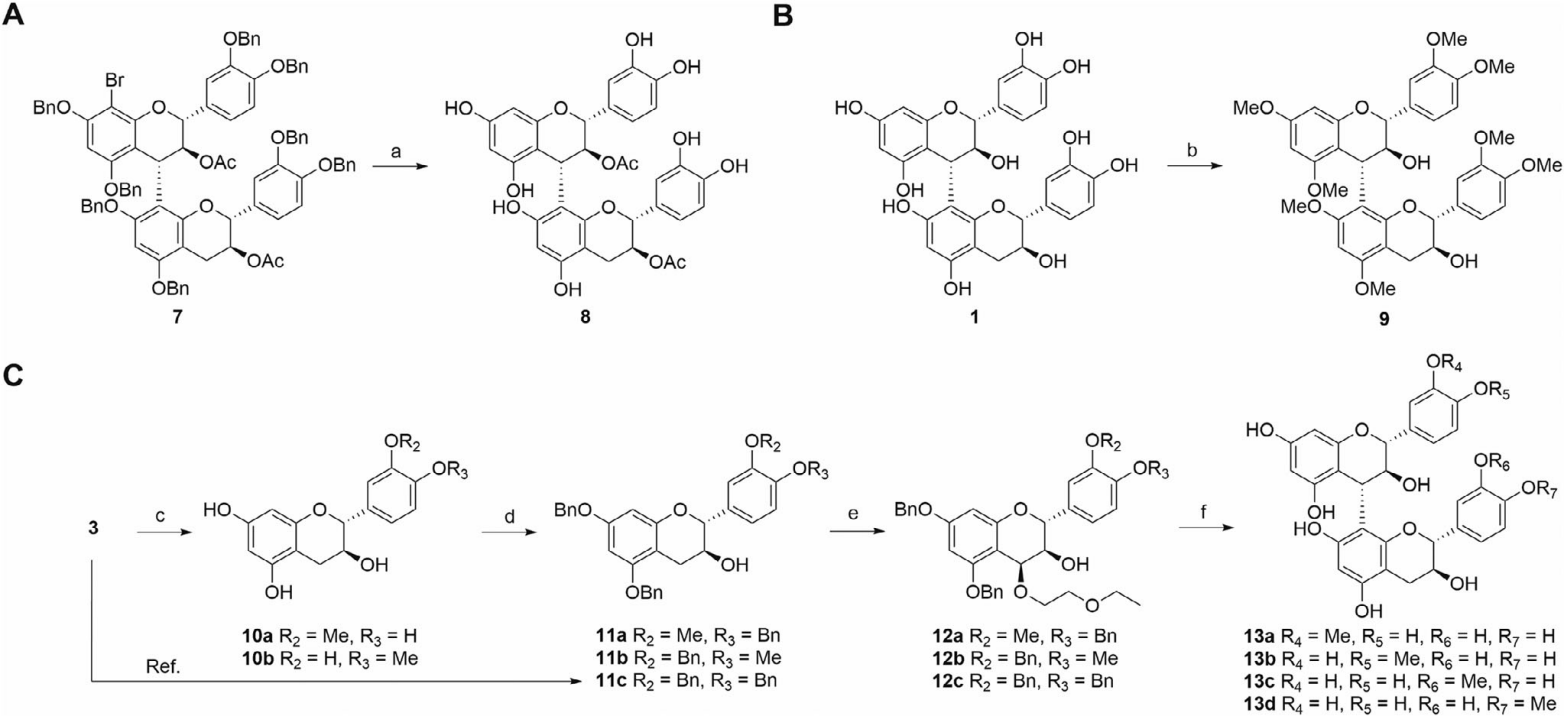
Synthesis of hydroxyl substituted derivatives of PB3. Reagents and conditions: a) 20% Pd(OH)_2_/C, H_2_, NaHCO_3_, MeOH, r.t., 6 h, 77%; b) dimethyl sulfate, cesium carbonate, DMF, 100 °C, 2 h, 44%; c) K_2_CO_3_, CH_3_I, acetone, r.t., 48 h, 8%; d) K_2_CO_3_, BnBr, DMF, r.t., 48 h, 56%–89%; e) 2‐ethoxyethanol, 2,3‐dicyano‐5,6‐dichlorobenzoquinone, DCM, r.t., 3 h, 44%‐53%; f) 1) Ac_2_O, triethylamine, 4‐dimethylaminopyridine, DCM, r.t., 3 h; 2) *N*‐Bromosuccinimide, DCM, r.t., 3 h; 3) 11, trimethylsilyl trifluoromethanesulfonate, DCM, −60 °C, 30 min; 4) NaOH, THF/MeOH, reflux, 4 h; 5) 20% Pd(OH)_2_/C, H_2_, NaHCO_3_, THF/MeOH, r.t., 6 h, 12%‐24%. DMF = *N*,*N*’‐dimethylformamide, r.t. = room temperature, DCM = dichloromethane, THF = tetrahydrofuran.

Increasing lipophilicity can be achieved by introducing substituents. We previously studied the synthetic process of PB3.^[^ ^47^ ^]^ In the synthetic route of PB3, intermediate **4** has an aromatic bromine structure and is prone to chemical reactions. Based on intermediate **4**, different substituents can be introduced through the Suzuki reaction, ^[^ ^48^ ^]^ followed by hydrogenolysis to obtain PB3 derivatives **6a**–**6h** (**Scheme** **1**). Derivatization of hydroxyl groups can also increase lipophilicity. In the derivatization of alcohol hydroxyl groups, we could obtain acetylated product **8** by hydrogenolysis using compound **7** as a starting material, and **7** could be prepared from procedures we reported before (**Scheme** **2**A).^[^ ^47^ ^]^ When dimethyl sulfate was used as a methylating agent, PB3 (**1**) underwent methylation to obtain product **9**, and all phenolic hydroxyl groups of **9** were methylated (Scheme 2B). To achieve selective methylation, we synthesized **10a** and **10b** from (+)‐catechin (**3**) under the conditions described in the literature.^[^ ^49^ ^]^ **10a** and **10b** were subjected to benzylation and C4 activation to give **12a** and **12b**, and then **11c** and **12c** were prepared using the methods we previously described.^[^ ^47^ ^]^ The mono‐substituted derivatives **13a**–**13d** were obtained through a continuous five‐step process (Scheme 2C).^[^ ^47^ ^]^

To assess the pharmacological properties of PB3 and its derivatives, we conducted an evaluation of their neuroprotective effects under OGD/R injury in the HT22 cells, using neuroprotective agent edaravone (EDA) ^[^ ^50^ ^]^ as the positive control (Table 1). The establishment of the OGD/R model is the same as mentioned above. Meanwhile, logD_7.4_ was calculated ^[^ ^51^ ^]^ to evaluate the lipophilicity of these compounds. The results showed that in the C8‐substituted derivatives of PB3, when the substituent is an alkyl group, the neuroprotective effects of PB3 derivatives first increase and then decrease with the increase of the substituent volume. Compound **6c** substituted with an isopropyl group, has the best neuroprotective activity. The substitution of aryl groups has a limited effect; **6** **h** has a slight improvement in activity but is still not as good as **6c**. The effect of alcohol hydroxyl substituted **8** on cell survival is almost the same as **1**. Phenolic hydroxyl groups are important functional groups that exert activity, and when phenolic hydroxyl groups were derivatized, the activity of those derivatives decreased to varying degrees. The activity of PB3 derivatives could be affected by many other factors with the increase of lipophilicity, and it is important to weigh both potency and lipophilicity together when selecting preferred compounds for drug design.^[^ ^52^ ^]^ Here, the most effective compound **6c** was selected for further study. Following that, we have evaluated the transport efficiency of **1** and **6c** using an in vitro BBB model in bEnd.3 cells (Figure S8, Supporting Information).^[^ ^53^ ^]^ The results indicate that the transport efficiency of **6c** is better than that of **1**, which initially validated our hypothesis.

**Table 1:** In vitro neuroprotective effects of PB3 derivatives.

Compound	Cell viability (%)		clogD_7.4_ ^a)^
	2 µM	20 µM	
**1**	63.87 ± 3.36	70.89 ± 6.71^*^	1.518
**6a**	56.72 ± 2.22	70.22 ± 2.92^*^	1.656
**6b**	62.01 ± 9.21	73.56 ± 4.78^**^	1.741
**6c**	66.05 ± 1.97	83.59 ± 4.34^***^	1.969
**6d**	62.14 ± 3.73	78.75 ± 1.43^***^	2.148
**6e**	58.55 ± 8.12	66.98 ± 2.70	2.544
**6f**	59.70 ± 7.43	70.44 ± 7.72^*^	2.252
**6g**	55.88 ± 8.56	71.41 ± 1.74^*^	1.507
**6h**	58.77 ± 7.12	75.29 ± 4.68^**^	1.500
**8**	61.98 ± 5.58	69.29 ± 6.73^*^	1.895
**9**	52.61 ± 7.34	66.76 ± 1.89	3.147
**13a**	59.64 ± 2.82	65.51 ± 4.74	1.778
**13b**	57.52 ± 7.11	70.54 ± 0.41^*^	1.747
**13c**	66.18 ± 10.59	66.08 ± 2.90	1.738
**13d**	63.04 ± 6.27	69.03 ± 8.76	1.696
**EDA**	60.82 ± 7.40	71.53 ± 9.84^*^	–
**OGD/R**	50.88 ± 6.50		–

The data were presented as the percentage of surviving cells relative to control cells and as the mean ± S.D., *n* = 3. ^*^ *p* < 0.05, ^**^ *p* < 0.01, ^***^ *p* < 0.001 versus OGD/R, one‐way ANOVA, Dunnett's multiple comparison test.

^a)^ LogD_7.4_ was calculated by ADMETlab 3.0 program.^[^ ^51^ ^]^

Additionally, we investigated the effects of PB3, **6c**, and EDA on apoptosis at a concentration of 50 µm. The results suggested that compared with the model group, administration of **6c** could reduce the rate of TUNEL‐positive cells (Figure S9A, Supporting Information), increase the expression of Bcl‐2 (Figure S9B, Supporting Information), and decrease the expression of cleaved caspase‐3 (Figure S9C, Supporting Information). Among PB3, **6c**, and EDA, compound **6c** showed relatively better anti‐apoptotic activity. SPR analysis has shown that **6c** interacts with G3BP1 (*K*_D_ = 58 nm, Figure S10A, Supporting Information), and the target affinity of **6c** was not significantly enhanced compared to that of **1**. Therefore, the improvement in target accessibility could be one of the main reasons for the enhanced activity of **6c**. The binding mode of **6c** and the NTF2L domain of G3BP1 was analyzed by molecular docking (Figure S10B, Supporting Information). The results showed that **6c** interacts with residues such as Leu105, Arg107, Gln134, and Asp135, which is consistent with the simulation results of PB3. Compound **6c** can also mediate SG upregulation in the OGD/R model (Figure S11, Supporting Information), and the mechanism by which **6c** exerts its activity is similar to that of PB3. Cytotoxicities of **1** and **6c** were determined by the detection of 50% cytotoxic concentration using the CCK‐8 assay. Primary rat cortical neurons ^[^ ^10^ ^]^ were exposed to 200 µm gradient concentrations of compounds for 24 h. The 50% cytotoxic concentrations of **1** and **6c** on primary neurons were both over 200 µm, which demonstrated that the compounds have good safety.

### 2.5 In Vivo Evaluation of Preferred Compounds

**Figure 5:**
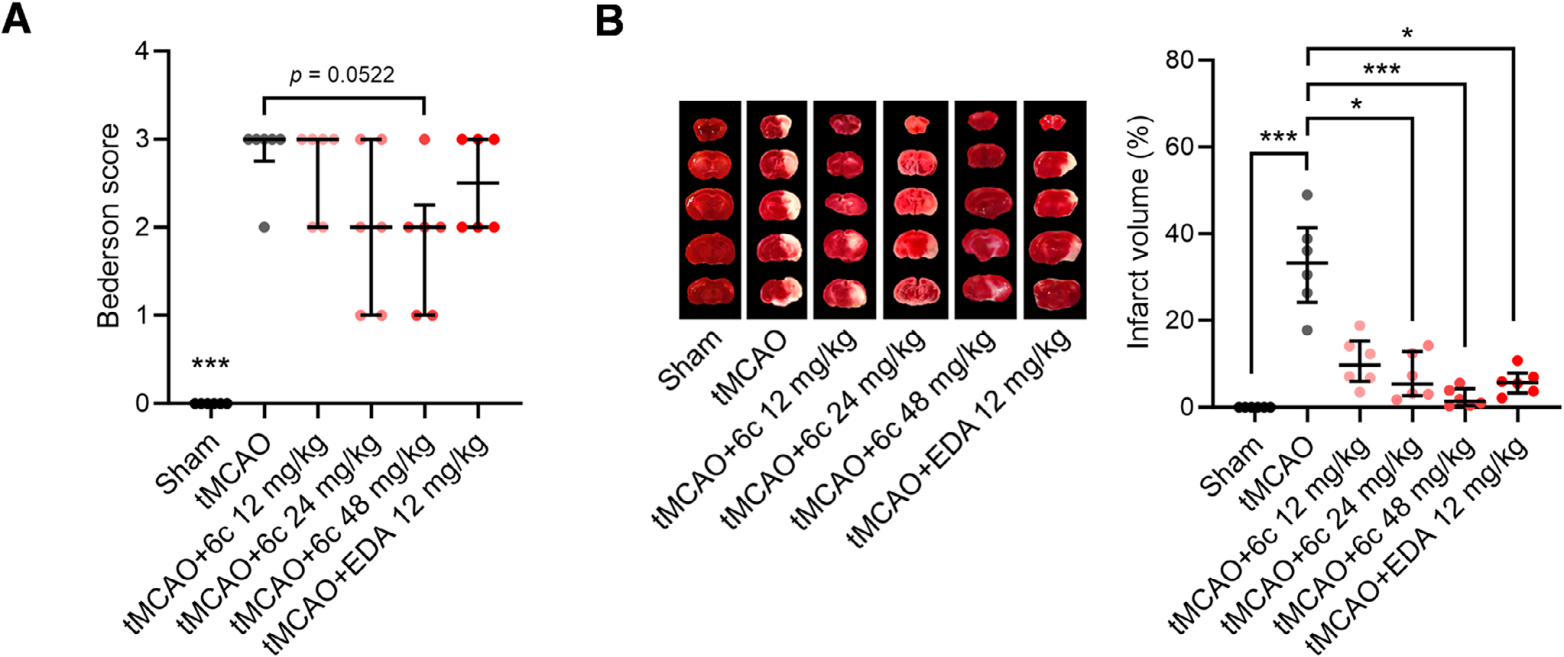
Effect of Compound 6c on infarct area and neurological function after 1.5 h of MCAO and 24 h of reperfusion. A) Neurological deficit score. Data are shown as median (IQR), ^***^ *p* < 0.001 versus tMCAO, Kruskal‐Wallis with Dunn's test (*n* = 6). B) Representative picture of 2,3,5‐triphenyltetrazolium chloride staining and quantification of infarct volumes. Data are shown as median (IQR), ^*^ *p* < 0.05, ^***^ *p* < 0.001, Kruskal‐Wallis with Dunn's test (*n* = 6).

The in vivo anti‐ischemic stroke activities of PB3, **6b,** and **6c** were preliminarily screened. EDA was set as the positive control. Mice were subjected to ischemia for an hour in a tMCAO model, followed by *i.p*. injection of the test compounds after reperfusion. According to the neurological deficit score assessment conducted 24 h after reperfusion, compound **6c** showed a more pronounced trend toward improving neurological deficits in tMCAO mice (Figure S12A, Supporting Information). The treatment with **6c** reduced the infarct volume by ≈30%, which was better compared to PB3 and **6b** (Figure S12B, Supporting Information). These results are consistent with those in the in vitro OGD/R model. The effects of **6c** at different doses on infarct area and neurological function after 1.5 h of MCAO and 24 h of reperfusion are shown in **Figure** **5**. The results suggest that the preferred compound **6c** has a therapeutic effect on ischemic injury in a dose‐dependent manner. Compared with the model group, treatment with 48 mg kg^−1^ of compound **6c** showed the best neuroprotective activity. An additional study evaluated the improvement of **6c** on the long‐term neurological recovery after tMCAO (Figure S13A, Supporting Information).^[^ ^54^ ^]^ The body weight of tMCAO mice decreased rapidly within 4 days, whereas treatment with **6c** daily mitigated the tendency toward weight loss (Figure S13B, Supporting Information). Modified neurological severity scores were established to evaluate the functional recovery of cured tMCAO mice, and **6c**‐treated mice exhibited lower scores than the tMCAO group (Figure S13C, Supporting Information). The rotarod test represents the neuromuscular function and motor coordination recovery. At the end of treatment, **6c**‐treated mice showed improvements compared with the tMCAO group (Figure S13D, Supporting Information). Besides, **6c** (48 mg kg^−1^, *i.p*.) can also mediate SG upregulation in ischemic brain tissue of mice (Figure S14, Supporting Information), confirming **6c**’s mechanism of action in vivo.

Preliminary pharmacokinetic studies suggest that **6c** might be metabolized faster than **1** in mice. After 1.5 h of MCAO followed by reperfusion, each of **1** and **6c** was administered at a dose of 48 mg kg^−1^ intraperitoneally to mice, and the brain and plasma concentrations of each compound were measured at 0.5 h after administration. The ratios of brain to plasma concentrations of **1** and **6c** were 0.37 and 0.57, respectively, confirming that the BBB permeability of compounds benefited from higher lipophilicity (**Table** **2**). The decrease in **6c** concentration in vivo may be due to changes in metabolic routes. Tertiary carbon atoms and terminal carbon atoms are easily metabolized through oxidative pathways,^[^ ^55^ ^]^ so the introduction of isopropyl groups leads to the production of more metabolites, resulting in a decrease in the area under curve (AUC). The metabolites of oligomeric proanthocyanidins and their analogues are quite complex. Although there are a large number of metabolites, most of them have a flavan‐3‐ol core structure and contain many phenolic hydroxyl groups.^[^ ^56^ ^]^ As mentioned above, phenolic hydroxyl groups are important active functional groups to exert neuroprotective effects, so it can be inferred that metabolites of **6c** also have similar activity to **6c**. In future work, radiolabeled pharmacokinetic studies and metabolite studies are needed to further elucidate the metabolism of the preferred compound.^[^ ^57^ ^]^

**Table 2:** Pharmacokinetic and BBB assessment of 1 and 6c.

	1	6c
**T_1/2_ (h)**	4.43 ± 0.34	2.01 ± 0.26
**C_max_ (ng/mL)**	30433 ± 1790	25233 ± 2501
AUC_last_ (h*ng/mL)	22219 ± 1942	10246 ± 1253
AUC_Inf_ (h*ng/mL)	22311 ± 1969	10376 ± 1277
**MRT_Inf_ (h)**	1.42 ± 0.06	0.76 ± 0.03
**C_plasma_ (ng/mL)** ^a)^	13048 ± 757	4955 ± 1591
**C_Brain_ (ng/g)** ^a)^	4786 ± 1613	2806 ± 489
**C_Brain_/C_plasma_** ^a)^	0.37	0.57

Data are expressed as mean ± S.D., *n* = 3.

^a)^ Measurement at 0.5 h after administration.

## 3 Conclusion

IS is a serious disease associated with high rates of mortality and disability. Currently, thrombolysis and thrombectomy are the primary interventions for IS patients. Although the current recanalization rate is impressive, only ≈10% of patients are neurologically normal.^[^ ^58^ ^]^ The goal of providing effective neuroprotection for IS patients is still elusive. In this study, we conducted a chemical proteomic study using PB3, a compound with neuroprotective effects against IS, to search for a novel therapeutic target of IS. Through a series of experiments, G3BP1 was identified as the potential target of PB3. Further mechanism study suggested PB3 exerts neuroprotective activity via inhibiting SG degradation and reducing apoptosis. Additionally, a structural modification study was performed to enhance the potency of PB3. The preferred compound **6c** exhibits significant anti‐ischemic stroke activity and improved BBB penetration capacity in both in vitro and in vivo models, while maintaining a similar mechanism of action to PB3. Taken together, we revealed that PB3 and its derivatives alleviate neuronal injury by targeting G3BP1 and regulating SG dynamics. The pharmacological targeting of G3BP1 offers new opportunities for combining neuroprotection with recanalization therapy for IS. Moreover, the development of PB3 and its derivatives provides new ideas for the discovery of neuroprotective agents in IS treatment.

## 4 Experimental Section

### Chemical Synthesis

All starting materials were commercially available and used without further purification unless otherwise stated. Compounds **4**, **7**, **11c,** and **12c** were synthesized according to previously reported methods.^[^ ^47^ ^]^ TLC analysis was performed using pre‐coated glass plates. Column chromatography was performed using silica gel (100–200 mesh) or octadecylsilyl silica gel (50 µm) for separation and purification. Specific optical rotations were determined using an Antopol IV polarimeter (Rudolph, USA). NMR spectra were recorded on a DRX‐400 spectrometer (Bruker, USA) with CDCl_3_, CD_3_OD, D_2_O or DMSO‐*d_6_* as solvents. Resonances (*δ*) are given in parts per million relatives to tetramethylsilane or a residual solvent peak (CDCl_3_: ^1^H: *δ* = 7.26 ppm, ^13^C: *δ* = 77.16 ppm; CD_3_OD: ^1^H: *δ* = 3.31 ppm, ^13^C: *δ* = 49.00 ppm; D_2_O: ^1^H: *δ* = 4.80 ppm; DMSO‐*d_6_*: ^1^H: *δ* = 2.50 ppm). Data are reported as follows: chemical shift; multiplicity (s = singlet, d = doublet, t = triplet, m = multiple, dd = doublet of doublets); coupling constants (Hz); and integration. UHPLC‐HRMS analyses were performed using a Vanquish UHPLC system (Thermo Scientific, USA) coupled with a Q Exactive Focus mass spectrometer (Thermo Scientific, USA). Analytical HPLC was performed on Waters e2695 (Waters, USA). Analytical HPLC conditions: Column: Waters XBridge Phenyl 5 µm, 4.6 × 250 mm (Waters, USA); Eluent: A: 0.1% FA H_2_O B: 0.1% FA acetonitrile (ACN); Flow rate: 1 mL min^−1^; Volume: 1.0 µL; Wavelength: 280 nm; Elution gradient: 5%–95% mobile phase B for 15 min.

### Synthesis of Phenyl(4‐((prop‐2‐yn‐1‐ylamino)methyl)phenyl)methanone (**S2**)

Propargylamine (620 mg, 11.26 mmol) was dissolved in dichloromethane (DCM)/ACN (1:2, 7.5 mL), then K_2_CO_3_ (2.7 g, 19.55 mmol) and 4‐(bromomethyl)benzophenone (**S1**, 1.3 g, 4.72 mmol) were added. The mixture was stirred overnight at room temperature. After completion of the reaction, the reaction mixture was concentrated. The semisolid was dissolved in EtOAc (10 mL) and washed with water (10 mL), and the organic layer was concentrated under reduced pressure and purified by silica gel column chromatography using petroleum ether/EtOAc (7:1 to 3:1) as eluent to give product **S2** (817 mg, 72%) as a light‐yellow oil. ^1^H‐NMR (400 MHz, CDCl_3_, *δ* ppm): 7.84–7.37 (m, 9H), 3.92 (s, 2H), 3.41 (d, *J* = 2.4 Hz, 2H), 2.31 (t, *J* = 2.4 Hz, 1H). ESI‐MS *m/z*: 250.1 [M+H]^+^.

### Synthesis of N‐(4‐Benzoylbenzyl)‐2‐Bromo‐N‐(prop‐2‐yn‐1‐yl)Acetamide (**S3**)

Compound **S2** (817 mg, 3.28 mmol) was dissolved in DCM (5 mL), and saturated sodium bicarbonate solution (5 mL) was added. Then, 2‐Bromoacetyl bromide (989 mg, 4.90 mmol) was added in batches at −10 °C. The mixture was stirred overnight at room temperature. After completion of the reaction, the organic layer was concentrated under reduced pressure and purified by silica gel column chromatography using petroleum ether/EtOAc (6:1) as eluent to give product **S3** (867 mg, 72%) as a light‐yellow oil. ^1^H‐NMR (400 MHz, CDCl_3_, 3:2 mixture of rotational isomers, *δ* ppm): 7.89–7.33 (m, 9H), 4.82 (s, 0.8H), 4.77 (s, 1.2H), 4.23 (d, *J* = 2.2 Hz, 0.8H), 4.08 (d, *J* = 2.2 Hz, 1.2H), 4.01 (s, 1.2H), 3.88 (s, 0.8H), 2.37 (t, *J* = 2.2 Hz, 0.6H), 2.26 (t, *J* = 2.2 Hz, 0.4H). ^13^C‐NMR (100 MHz, CDCl_3_, 3:2 mixture of rotational isomers, *δ* ppm): 196.24, 195.98, 167.01, 166.80, 140.80, 140.07, 137.50, 137.32, 137.11, 132.71, 132.56, 130.83, 130.60, 130.03, 128.43, 128.37, 127.88, 126.79, 77.68, 77.36, 73.96, 73.18, 50.92, 49.09, 37.59, 35.20, 25.98. HRMS (ESI) Calcd for C_19_H_17_BrNO_2_ [M+H]^+^ 370.0443, found 370.0411.

### Synthesis of 3,5,7,3′,4′‐Penta‐O‐Tert‐Butyldimethylsilyl‐(+)‐Catechin (**S4**)

(+)‐Catechin (**3**, 4.0 g, 13.79 mmol), imidazole (16.6 g, 0.11 mol), and *t*‐butyldimethylchlorosilane (15.0 g, 0.22 mol) were dissolved in *N*,*N*’‐dimethylformamide (DMF, 20 mL). The mixture was stirred for 18 h at room temperature under N_2_. After completion of the reaction, EtOAc (100 mL) was added, and the mixture was washed with water (100 mL × 3). The organic layer was dried over anhydrous Na_2_SO_4_, then concentrated under reduced pressure and purified by silica gel column chromatography using petroleum ether/DCM (5:1) as eluent to give product **S4** (11.5 g, 97%) as a colorless oil. ^1^H‐NMR (400 MHz, CDCl_3_, *δ* ppm): 6.92 (d, *J* = 2.2 Hz, 1H), 6.89 (dd, *J* = 8.2, 2.2 Hz, 1H), 6.85 (d, *J* = 8.2 Hz, 1H), 6.13 (d, *J* = 2.3 Hz, 1H), 5.99 (d, *J* = 2.3 Hz, 1H), 4.55 (d, *J* = 9.0 Hz, 1H), 3.90 (m, 1H), 3.09 (dd, *J* = 16.2, 6.0 Hz, 1H), 2.59 (dd, *J* = 16.2, 9.0 Hz, 1H), 1.05 (s, 9H), 1.03 (s, 9H), 1.02 (s, 9H), 1.00 (s, 9H), 0.80 (s, 9H), 0.28 (s, 3H), 0.27 (s, 3H), 0.25–0.19 (m, 18H), ‐0.10 (s, 3H), ‐0.28 (s, 3H). ESI‐MS *m/z*: 861.5 [M+H]^+^.

### Synthesis of 3,7,3′,4′‐Tetra‐O‐Tert‐Butyldimethylsilyl‐(+)‐Catechin (**S5**)

Compound **S4** (11.5 g, 13.35 mmol) was dissolved in DCM (50 mL), and trifluoroacetic acid (1.6 mL) was added in batches at 0 °C. The mixture was stirred for 18 h at room temperature. After completion of the reaction, the reaction mixture was quenched by the addition of the saturated sodium bicarbonate solution. The organic layer was concentrated under reduced pressure and purified by silica gel column chromatography using petroleum ether/DCM (3:1) as eluent to give product **S5** (954 mg, 10%) as a light‐yellow oil. ^1^H‐NMR (400 MHz, CDCl_3_, *δ* ppm): 6.88 (d, *J* = 2.4 Hz, 1H), 6.85 (dd, *J* = 8.0, 2.4 Hz, 1H), 6.82 (d, *J* = 8.0 Hz, 1H), 6.06 (d, *J* = 2.2 Hz, 1H), 5.96 (d, *J* = 2.2 Hz, 1H), 4.55 (d, *J* = 9.3 Hz, 1H), 3.90 (m, 1H), 2.97 (dd, *J* = 15.7, 5.8 Hz, 1H), 2.59 (dd, *J* = 15.7, 9.3 Hz, 1H), 1.00 (s, 9H), 0.99 (s, 9H), 0.96 (s, 9H), 0.78 (s, 9H), 0.22–0.15 (m, 18H), ‐0.09 (s, 3H), ‐0.37 (s, 3H). ESI‐MS *m/z*: 745.8 [M‐H]^−^.

### Synthesis of N‐(4‐Benzoylbenzyl)‐2‐(((2R,3S)‐2‐(3,4‐dihydroxyphenyl)‐3,7‐dihydroxychroman‐5‐yl)oxy)‐N‐(prop‐2‐yn‐1‐yl)acetamide (**S6**)

Compound S3 (519 mg, 1.41 mmol) was dissolved in acetone (5 mL), and K_2_CO_3_ (442 mg, 3.20 mmol) was added. The mixture was stirred for 10 min, then compound **S5** (954 mg, 1.28 mmol) was added. Subsequently, the mixture was stirred for 18 h at room temperature in the dark. The mixture was filtered, and the filtrate was concentrated under reduced pressure and purified by silica gel column chromatography using petroleum ether/EtOAc (6:1) as eluent to give the intermediate 1. Intermediate 1 was dissolved in tetrahydrofuran (THF, 5 mL) and cooled to 0 °C. Acetic acid (0.26 mL) and 1 m tetrabutylammonium fluoride (2.6 mL) were added. The mixture was stirred for 45 min in the dark. After completion of the reaction, the reaction mixture was concentrated under reduced pressure and purified by octadecylsilyl silica gel column chromatography using water/MeOH (1:9) as eluent to give the intermediate 2. Intermediate 2 was dissolved in THF (19.2 mL), FA (9.6 mL), and water (3.2 mL) were added. The mixture was stirred overnight at room temperature in the dark. After completion of the reaction, the reaction mixture was concentrated under reduced pressure and purified by octadecylsilyl silica gel column chromatography using water/MeOH (3:7) as eluent to give product **S6** (155 mg, 21% yield for 3 steps) as a light‐yellow oil. [α]^25^_D_+3.8° (c 1.00, CH_3_OH). ^1^H‐NMR (400 MHz, CD_3_OD, 3:2 mixture of rotational isomers, δ ppm): 7.82–7.32 (m, 9H), 6.83 (d, *J* = 2.1 Hz, 0.6H), 6.78 (d, *J* = 2.1 Hz, 0.4H), 6.77–6.67 (m, 1.6H), 6.63 (dd, *J* = 8.4, 2.1 Hz, 0.4H), 6.03 (d, *J* = 2.2 Hz, 0.6H), 6.00 (d, *J* = 2.2 Hz, 0.6H), 5.97 (d, *J* = 2.2 Hz, 0.4H), 5.95 (d, *J* = 2.2 Hz, 0.4H), 4.95 (s, 2H), 4.83–4.80 (m, 2H), 4.61 (d, *J* = 7.7 Hz, 0.6H), 4.42 (d, *J* = 7.9 Hz, 0.4H), 4.29–4.24 (m, 2H), 4.00 (m, 0.6H), 3.80 (m, 0.4H), 2.96 (dd, *J* = 16.1, 5.4 Hz, 0.6H), 2.83 (t, *J* = 2.5 Hz, 0.6H), 2.72–2.65 (m, 0.8H), 2.61 (dd, *J* = 16.4, 7.7 Hz, 0.6H), 2.27 (dd, *J* = 16.3, 7.9 Hz, 0.4H). ^13^C‐NMR (100 MHz, CD_3_OD, 3:2 mixture of rotational isomers, δ ppm): 198.20, 198.08, 170.94, 170.81, 158.38, 158.36, 158.23, 156.91, 146.25, 143.01, 142.61, 138.81, 138.72, 138.07, 137.99, 133.81, 132.08, 131.66, 131.44, 131.05, 130.99, 129.54, 128.97, 127.97, 120.12, 119.94, 116.10, 116.07, 115.41, 115.21, 102.26, 97.35, 94.06, 93.82, 82.91, 82.82, 78.96, 78.78, 75.28, 74.03, 68.60, 68.52, 68.43, 67.87, 50.95, 50.10, 37.66, 36.11, 28.62, 28.28. HRMS (ESI) Calcd for C_34_H_30_NO_8_ [M+H]^+^ 580.1971, found 580.1915.

### Synthesis of 4β‐Benzylsuifanyl‐(+)‐Catechin (**S8**)

To a stirred solution of taxifolin (**S7**, 250 mg, 0.82 mmol) in ethanol (10 mL) under N_2_ was added NaBH_4_ (200 mg, 5.29 mmol) at 0 °C. After stirring for 30 min, acetic acid (20 mL) and benzyl mercaptan (0.5 mL) were added. The mixture was stirred overnight at room temperature. After completion of the reaction, EtOAc and water were added, and the organic layer was concentrated under reduced pressure and purified by octadecylsilyl silica gel column chromatography using water/MeOH (2:3) as eluent to give product **S8** (64 mg, 47%) as a light‐yellow oil. ^1^H‐NMR (400 MHz, acetone‐*d*_6_, *δ* ppm): 7.46–7.16 (m, 5H), 6.95 (s, 1H), 6.84–6.82 (m, 2H), 6.03 (d, *J* = 2.3 Hz, 1H), 5.83 (d, *J* = 2.3 Hz, 1H), 4.96 (d, *J* = 9.6 Hz, 1H), 4.39 (d, *J* = 4.2 Hz, 1H), 4.16 (m, 1H), 4.14 (d, *J* = 12.4 Hz, 1H), 4.08 (d, *J* = 12.4 Hz, 1H). ESI‐MS *m/z*: 435.2 [M+Na]^+^.

*Synthesis of N‐(4‐Benzoylbenzyl)‐2‐(((2R,2′R,3S,3′S,4S)‐2,2′‐bis(3,4‐dihydroxyphenyl)‐3,3′,5,7,7′‐pentahydroxy‐[4,8′‐bichroman]‐5′‐yl)oxy)‐N‐(prop‐2‐yn‐1‐yl)acetamide (PB3‐BP, **2**)*

Compound **S8** (22 mg, 0.05 mmol) and **S6** (155 mg, 0.27 mmol) were dissolved in acetone (5 mL) and cooled to 0 °C. AgBF_4_ (26 mg, 0.13 mmol) was added, and the mixture was stirred for 30 min in the dark. After completion of the reaction, the reaction mixture was concentrated under reduced pressure, and EtOAc and water were added. The organic layer was concentrated under reduced pressure and purified by octadecylsilyl silica gel column chromatography using water/MeOH (2:3) as eluent to give product **2** (15 mg, 32%) as a light‐yellow oil. HRMS (ESI) Calcd for C_49_H_42_NO_14_ [M+H]^+^ 868.2605, found 868.2589.

*Synthesis of (2R,2′R,3S,3′S,4S)‐5′‐(2‐((4‐Benzoylbenzyl)(prop‐2‐yn‐1‐yl)amino)‐2‐oxoethoxy)‐2,2′‐bis(3,4‐diacetoxyphenyl)‐[4,8′‐bichromane]‐3,3′,5,7,7′‐pentayl pentaacetate (**S9**)*

Compound **2** (39 mg, 44.97 µmol), 4‐dimethylaminopyridine (DMAP, 3 mg, 24.56 µmol), and Ac_2_O (138 mg, 1.35 mmol) were added in pyridine (1.6 mL). The mixture was stirred for 90 min at room temperature in the dark. After completion of the reaction, EtOAc and water were added, and the organic layer was washed with 1 m HCl. Then, the organic layer was concentrated under reduced pressure and purified by silica gel column chromatography using DCM/MeOH (40:1) as eluent to give product **S9** (29 mg, 52%) as a light‐yellow oil. [α]^25^_D_‐169.0° (c 0.10, CH_2_Cl_2_). ^1^H‐NMR (400 MHz, CDCl_3_, 1:1 mixture of rotational isomers, δ ppm): 7.87–6.63 (m, 15H), 6.53–6.25 (m, 3H), 5.64 (m, 1H), 5.14–4.64 (m, 6.5H), 4.43–4.01 (m, 3.5H), 3.09 (dd, *J* = 17.6, 5.9 Hz, 0.5H), 2.95 (dd, *J* = 17.2, 5.6 Hz, 0.5H), 2.79–2.66 (m, 1H), 2.40–1.81 (m, 28H). ^13^C‐NMR (100 MHz, CDCl_3_, 1:1 mixture of rotational isomers, δ ppm): 202.15, 196.32, 170.29, 169.31, 168.85, 168.74, 168.50, 168.17, 168.06, 167.98, 167.96, 167.80, 156.30, 155.02, 152.91, 152.70, 149.68, 149.15, 148.28, 142.21, 142.13, 142.08, 141.69, 141.66, 141.60, 140.83, 140.38, 137.62, 137.22, 135.58, 135.54, 132.64, 130.93, 130.83, 130.67, 130.13, 130.11, 128.52, 128.45, 128.25, 126.83, 126.61, 125.07, 124.72, 124.58, 123.63, 123.54, 122.80, 122.09, 121.80, 115.94, 113.61, 113.35, 110.04, 108.41, 108.03, 99.29, 99.27, 79.12, 79.08, 77.89, 77.87, 74.18, 72.93, 70.97, 68.63, 68.25, 67.86, 48.46, 48.44, 36.62, 34.75, 34.73, 25.03, 21.21–20.48 (CH_3_Ac). HRMS (ESI) Calcd for C_67_H_60_NO_23_ [M+H]^+^ 1246.3556, found 1246.3553.

### Synthesis of 5,7,3′,4′‐Tetra‐O‐Benzyl‐8‐Methyl‐(+)‐Catechin‐(4α,8)‐[5,7,3′,4′‐Tetra‐O‐Benzyl‐(+)‐Catechin] (**5a**)

To a stirred solution of compound **4** (400 mg, 0.29 mmol), methylboronic acid (26 mg, 0.43 mmol), and K_2_CO_3_ (120 mg, 0.87 mmol) in 1,4‐dioxane/ACN/water (3:3:1, 7 mL) under N_2_ was added Pd(PPh_3_)_4_ (34 mg, 0.03 mmol). The mixture was stirred for 42 h under reflux. After completion of the reaction, the mixture was filtered, and the organic layer of filtrate was concentrated under reduced pressure and purified by silica gel column chromatography using petroleum ether/EtOAc (3:1) as eluent to give product **5a** (281 mg, 74%) as a light‐yellow solid. [α]^25^_D_‐128.5° (*c* 1.00, CH_2_Cl_2_). ^1^H‐NMR (400 MHz, CDCl_3_, 2:1 mixture of rotational isomers, *δ* ppm): 7.52–6.61 (m, 46H), 6.25 (s, 0.67H), 6.19 (s, 0.33H), 6.14 (s, 0.67H), 6.07 (s, 0.33H), 5.28–4.32 (m, 18.33H), 4.22 (m, 0.67H), 4.05 (m, 0.33H), 3.75–3.52 (m, 1.67H), 3.19 (dd, *J* = 16.4, 6.1 Hz, 0.33H), 3.03 (dd, *J* = 16.2, 5.4 Hz, 0.67H), 2.61 (dd, *J* = 16.4, 9.8 Hz, 0.33H), 2.39 (dd, *J* = 16.2, 8.8 Hz, 0.67H), 1.97 (s, 2.01H), 1.85 (s, 0.99H). ^13^C‐NMR (100 MHz, CDCl_3_, 2:1 mixture of rotational isomers, *δ* ppm): 157.05, 155.74, 155.62, 155.51, 155.43, 155.16, 154.96, 154.90, 153.96, 153.02, 149.27, 149.23, 149.13, 149.09, 149.04, 148.96, 148.87, 148.26, 137.87, 137.82, 137.56, 137.54, 137.45, 137.36, 137.33, 137.30, 137.24, 137.23, 132.57, 132.51, 131.91, 130.52, 128.72–127.13 (CHBn), 121.20, 120.67, 120.26, 120.22, 115.03, 114.96, 114.91, 114.71, 114.30, 114.09, 113.85, 113.71, 112.71, 112.66, 109.40, 109.27, 107.75, 103.59, 102.65, 92.46, 92.15, 91.94, 91.80, 81.78, 81.43, 81.36, 80.78, 73.73, 73.59, 71.49–70.00 (CH_2_Bn), 68.96, 68.52, 37.68, 37.62, 28.64, 28.10, 8.53, 8.45. HRMS (ESI) Calcd for C_87_H_77_O_12_ [M+H]^+^ 1313.5415, found 1313.5424.

### Synthesis of 5,7,3′,4′‐Tetra‐O‐Benzyl‐8‐Vinyl‐(+)‐Catechin‐(4α,8)‐[5,7,3′,4′‐Tetra‐O‐Benzyl‐(+)‐Catechin] (**5b**)

To a stirred solution of compound **4** (2240 mg, 1.63 mmol), potassium vinyltrifluoroborate (283 mg, 2.11 mmol), and K_2_CO_3_ (1530 mg, 11.07 mmol) in 1,4‐dioxane/water (4:1, 22.4 mL) under N_2_ was added Pd(PPh_3_)_4_ (226 mg, 0.20 mmol). The mixture was stirred for 24 h under reflux. After completion of the reaction, the mixture was filtered, and the organic layer of the filtrate was concentrated under reduced pressure and purified by silica gel column chromatography using petroleum ether/EtOAc (4:1) as eluent to give product **5b** (1520 mg, 71%) as a light‐yellow solid. [α]^25^_D_‐141.7° (*c* 1.00, CH_2_Cl_2_). ^1^H‐NMR (400 MHz, CDCl_3_, 7:3 mixture of rotational isomers, *δ* ppm): 7.51–6.71 (m, 48H), 6.24 (s, 0.7H), 6.16 (s, 0.3H), 6.15 (s, 0.7H), 6.07 (s, 0.3H), 5.94 (dd, *J* = 18.0, 2.9 Hz, 0.7H), 5.78 (dd, *J* = 18.0, 2.9 Hz, 0.3H), 5.20–4.44 (m, 18H), 4.37 (d, *J* = 8.9 Hz, 0.3H), 4.23 (m, 0.7H), 4.10 (m, 0.3H), 3.75–3.51 (m, 1.7H), 3.18 (dd, *J* = 16.4, 6.0 Hz, 0.3H), 3.03 (dd, *J* = 16.2, 5.6 Hz, 0.7H), 2.62 (dd, *J* = 16.4, 9.7 Hz, 0.3H), 2.38 (dd, *J* = 16.2, 9.2 Hz, 0.7H). ^13^C‐NMR (100 MHz, CDCl_3_, 7:3 mixture of rotational isomers, *δ* ppm): 157.04, 156.36, 156.23, 156.19, 156.13, 155.65, 155.63, 155.56, 155.50, 153.94, 153.09, 149.22, 149.12, 149.06, 149.02, 148.96, 148.84, 148.08, 137.83, 137.50, 137.48, 137.41, 137.37, 137.31, 137.27, 137.25, 137.23, 137.13, 137.10, 136.75, 132.15, 132.03, 131.83, 130.16, 128.72–127.07 (CHBn), 121.20, 120.63, 120.17, 120.14, 116.33, 116.30, 115.42, 114.92, 114.84, 114.58, 114.10, 113.91, 113.59, 113.52, 112.33, 112.15, 109.48, 109.42, 108.97, 103.62, 102.47, 92.25, 92.03, 91.78, 91.52, 82.08, 81.76, 81.46, 80.73, 73.44, 73.22, 71.42–69.97 (CH_2_Bn), 68.93, 68.53, 37.54, 37.47, 29.83, 28.05. HRMS (ESI) Calcd for C_88_H_77_O_12_ [M+H]^+^ 1325.5415, found 1325.5412.

### Synthesis of 5,7,3′,4′‐Tetra‐O‐Benzyl‐8‐(Prop‐1‐en‐2‐yl)‐(+)‐catechin‐(4α,8)‐[5,7,3′,4′‐Tetra‐O‐Benzyl‐(+)‐Catechin] (**5c**)

To a stirred solution of compound **4** (400 mg, 0.29 mmol), isopropenylboronic acid pinacol ester (63 mg, 0.37 mmol), and K_2_CO_3_ (273 mg, 1.98 mmol) in 1,4‐dioxane/water (4:1, 5 mL) under N_2_ was added Pd(PPh_3_)_4_ (40 mg, 0.03 mmol). The mixture was stirred for 40 h under reflux. After completion of the reaction, the mixture was filtered, and the organic layer of filtrate was concentrated under reduced pressure and purified by silica gel column chromatography using petroleum ether/EtOAc (3:1) as eluent to give product **5c** (323 mg, 83%) as a light‐yellow solid. [α]^25^_D_‐139.7° (*c* 1.60, CH_2_Cl_2_). ^1^H‐NMR (400 MHz, CDCl_3_, 2:1 mixture of rotational isomers, *δ* ppm): 7.47–6.57 (m, 46H), 6.18 (s, 0.67H), 6.17 (s, 0.33H), 6.15 (s, 0.67H), 6.09 (s, 0.33H), 5.21–4.43 (m, 20.33H), 4.25 (m, 0.67H), 4.05 (m, 0.33H), 3.76–3.59 (m, 1.67H), 3.09–2.95 (m, 1H), 2.64 (dd, *J* = 16.1, 8.0 Hz, 0.33H), 2.36 (dd, *J* = 16.4, 9.0 Hz, 0.67H), 1.89 (s, 2H), 1.76 (s, 1H). ^13^C‐NMR (100 MHz, CDCl_3_, 2:1 mixture of rotational isomers, *δ* ppm): 157.07, 155.95, 155.88, 155.61, 155.55, 154.59, 154.46, 154.07, 153.97, 153.93, 152.66, 149.23, 149.21, 149.07, 149.05, 148.99, 148.88, 148.85, 148.19, 138.37, 138.10, 137.85, 137.79, 137.75, 137.60, 137.56, 137.54, 137.41, 137.39, 137.30, 137.28, 137.24, 136.97, 132.57, 132.38, 131.90, 131.04, 128.67–126.85 (CHBn), 121.11, 120.59, 120.21, 119.86, 116.46, 115.37, 115.16, 115.03, 114.83, 114.63, 114.21, 114.08, 113.99, 113.74, 112.51, 112.20, 109.78, 109.16, 102.96, 102.62, 93.15, 92.44, 92.08, 91.98, 81.81, 81.38, 81.25, 80.76, 75.07, 73.43, 71.45–70.02 (CH_2_Bn), 68.66, 68.43, 37.77, 37.67, 28.13, 27.32, 24.80, 23.78. HRMS (ESI) Calcd for C_89_H_79_O_12_ [M+H]^+^ 1339.5572, found 1339.5579.

### Synthesis of 5,7,3′,4′‐Tetra‐O‐Benzyl‐8‐((E)‐Prop‐1‐en‐1‐yl)‐(+)‐Catechin‐(4α,8)‐[5,7,3′,4′‐Tetra‐O‐Benzyl‐(+)‐Catechin] (**5d**)

To a stirred solution of compound **4** (400 mg, 0.29 mmol), (E)‐prop‐1‐en‐1‐ylboronic acid (37 mg, 0.43 mmol), and K_2_CO_3_ (120 mg, 0.87 mmol) in 1,4‐dioxane/water (4:1, 5 mL) under N_2_ was added Pd(PPh_3_)_4_ (40 mg, 0.03 mmol). The mixture was stirred for 42 h under reflux. After completion of the reaction, the mixture was filtered, and the organic layer of filtrate was concentrated under reduced pressure and purified by silica gel column chromatography using petroleum ether/EtOAc (3:1) as eluent to give product **5d** (262 mg, 67%) as a light‐yellow solid. [α]^25^_D_‐178.4° (*c* 1.00, CH_2_Cl_2_). ^1^H‐NMR (400 MHz, CDCl_3_, 2:1 mixture of rotational isomers, *δ* ppm): 7.51–6.27 (m, 48H), 6.23 (s, 0.67H), 6.16 (s, 0.33H), 6.14 (s, 0.67H), 6.07 (s, 0.33H), 5.22–4.44 (m, 18H), 4.37 (d, *J* = 9.0 Hz, 0.33H), 4.21 (m, 0.67H), 4.09 (m, 0.33H), 3.72–3.52 (m, 1.67H), 3.18 (dd, *J* = 16.5, 6.3 Hz, 0.33H), 3.03 (dd, *J* = 16.5, 5.7 Hz, 0.67H), 2.62 (dd, *J* = 16.5, 9.9 Hz, 0.33H), 2.37 (dd, *J* = 16.5, 9.1 Hz, 0.67H), 1.81 (d, *J* = 6.5 Hz, 2H), 1.73 (d, *J* = 6.5 Hz, 1H). ^13^C‐NMR (100 MHz, CDCl_3_, 2:1 mixture of rotational isomers, *δ* ppm): 157.05, 155.68, 155.61, 155.56, 155.48, 155.40, 155.37, 155.05, 154.91, 154.01, 153.94, 153.13, 149.39, 149.28, 149.25, 149.22, 149.12, 149.02, 148.99, 148.23, 137.86, 137.82, 137.65, 137.59, 137.54, 137.48, 137.44, 137.36, 137.34, 137.30, 137.25, 137.19, 136.94, 136.82, 132.44, 132.30, 132.01, 131.91, 131.85, 130.31, 128.72–127.18 (CHBn), 121.65, 121.55, 121.41, 121.27, 120.73, 120.23, 115.16, 115.03, 114.70, 114.08, 113.73, 112.55, 112.46, 112.15, 109.74, 109.59, 109.47, 108.80, 103.68, 103.42, 102.64, 102.54, 92.92, 92.51, 91.88, 91.75, 82.01, 81.71, 81.52, 80.77, 73.56, 73.42, 71.49–70.02 (CH_2_Bn), 68.93, 68.54, 37.67, 37.59, 28.17, 28.10, 20.38, 20.34. HRMS (ESI) Calcd for C_89_H_79_O_12_ [M+H]^+^ 1339.5572, found 1339.5582.

### Synthesis of 5,7,3′,4′‐Tetra‐O‐Benzyl‐8‐(2‐Methylprop‐1‐en‐1‐yl)‐(+)‐Catechin‐(4α,8)‐[5,7,3′,4′‐Tetra‐O‐Benzyl‐(+)‐Catechin] (**5e**)

To a stirred solution of compound **4** (400 mg, 0.29 mmol), 2,2‐dimethylethenylboronic acid pinacol ester (69 mg, 0.38 mmol), and K_2_CO_3_ (273 mg, 1.98 mmol) in 1,4‐dioxane/water (4:1, 5 mL) under N_2_ was added Pd(PPh_3_)_4_ (40 mg, 0.03 mmol). The mixture was stirred for 40 h under reflux. After completion of the reaction, the mixture was filtered, and the organic layer of filtrate was concentrated under reduced pressure and purified by silica gel column chromatography using petroleum ether/EtOAc (3:1) as eluent to give product **5e** (377 mg, 96%) as a light‐yellow solid. [α]^25^_D_‐108.3° (c 1.00, CH_2_Cl_2_). ^1^H‐NMR (400 MHz, CDCl_3_, 7:3 mixture of rotational isomers, δ ppm): 7.52–6.61 (m, 46H), 6.20–6.03 (m, 2H), 5.94 (s, 0.7H), 5.81 (s, 0.3H), 5.23–4.41 (m, 18.3H), 4.24 (m, 0.7H), 4.09 (m, 0.3H), 3.76–3.60 (m, 1H), 3.56 (d, *J* = 8.6 Hz, 0.7H), 3.07–2.96 (m, 1H), 2.65 (dd, *J* = 16.8, 8.7 Hz, 0.3H), 2.35 (dd, *J* = 16.2, 9.5 Hz, 0.7H), 1.81 (s, 2.1H), 1.72 (s, 0.9H), 1.44 (s, 2.1H), 1.27 (s, 0.9H). ^13^C‐NMR (100 MHz, CDCl_3_, 7:3 mixture of rotational isomers, δ ppm): 157.04, 155.88, 155.80, 155.56, 155.43, 155.35, 155.21, 154.52, 154.49, 153.90, 152.60, 149.17, 149.14, 149.03, 148.97, 148.89, 148.84, 148.78, 147.98, 137.88, 137.85, 137.73, 137.48, 137.46, 137.37, 137.31, 137.28, 137.24, 136.92, 135.93, 135.74, 132.43, 131.89, 130.88, 128.62–126.82 (CHBn), 121.24, 120.89, 120.20, 119.87, 116.29, 114.93, 114.80, 114.57, 114.41, 113.93, 113.68, 112.44, 112.28, 110.40, 110.26, 109.73, 109.26, 102.88, 102.51, 93.12, 92.45, 91.86, 91.81, 81.84, 81.58, 81.11, 80.69, 73.50, 73.35, 71.50–69.96 (CH_2_Bn), 68.48, 68.39, 37.90, 37.70, 28.16, 27.36, 26.07, 25.97, 20.84, 20.66. HRMS (ESI) Calcd for C_90_H_81_O_12_ [M+H]^+^ 1353.5728, found 1353.5730.

### Synthesis of 5,7,3′,4′‐Tetra‐O‐Benzyl‐8‐Phenyl‐(+)‐Catechin‐(4α,8)‐[5,7,3′,4′‐Tetra‐O‐Benzyl‐(+)‐Catechin] (**5f**)

To a stirred solution of compound **4** (400 mg, 0.29 mmol), phenylboronic acid (53 mg, 0.43 mmol), and K_2_CO_3_ (120 mg, 0.87 mmol) in 1,4‐dioxane/water (4:1, 5 mL) under N_2_ was added Pd(PPh_3_)_4_ (34 mg, 0.03 mmol). The mixture was stirred for 42 h under reflux. After completion of the reaction, the mixture was filtered, and the organic layer of filtrate was concentrated under reduced pressure and purified by silica gel column chromatography using petroleum ether/EtOAc (3:1) as eluent to give product **5f** (336 mg, 64%) as a light‐yellow solid. [α]^25^_D_‐154.6° (*c* 1.00, CH_2_Cl_2_). ^1^H‐NMR (400 MHz, CDCl_3_, 53:47 mixture of rotational isomers, *δ* ppm): 7.55–6.47 (m, 51H), 6.30 (s, 0.47H), 6.25 (s, 0.53H), 6.22 (s, 0.53H), 6.11 (s, 0.47H), 5.23–4.36 (m, 18.47H), 4.20 (m, 0.53H), 4.01 (m, 0.47H), 3.77–3.55 (m, 1.53H), 3.19 (dd, *J* = 16.4, 5.8 Hz, 0.47H), 3.04 (dd, *J* = 16.3, 5.7 Hz, 0.53H), 2.63 (dd, *J* = 16.4, 9.7 Hz, 0.47H), 2.38 (dd, *J* = 16.3, 9.1 Hz, 0.53H). ^13^C‐NMR (100 MHz, CDCl_3_, 53:47 mixture of rotational isomers, *δ* ppm): 157.09, 156.44, 156.42, 155.66, 155.59, 155.53, 154.93, 154.85, 154.08, 153.96, 152.85, 149.64, 149.27, 149.06, 148.89, 148.66, 148.59, 147.85, 137.75, 137.61, 137.53, 137.43, 137.39, 137.35, 137.30, 137.26, 137.15, 136.85, 134.17, 133.88, 132.36, 132.24, 131.93, 131.87, 131.78, 130.38, 128.68–126.73 (CHBn), 126.09, 120.78, 120.31, 120.22, 120.06, 115.36, 115.04, 114.99, 114.74, 114.51, 113.74, 113.64, 113.60, 113.27, 112.31, 112.17, 110.03, 109.49, 103.37, 102.60, 93.49, 92.31, 91.98, 91.86, 81.47, 81.35, 81.11, 80.77, 73.62, 73.49, 71.66–70.01 (CH_2_Bn), 69.00, 68.46, 37.90, 37.76, 28.13. HRMS (ESI) Calcd for C_92_H_79_O_12_ [M+H]^+^ 1375.5572, found 1375.5575.

### Synthesis of 5,7,3′,4′‐Tetra‐O‐Benzyl‐8‐(Pyridin‐4‐yl)‐(+)‐Catechin‐(4α,8)‐[5,7,3′,4′‐Tetra‐O‐Benzyl‐(+)‐Catechin] (**5** **g**)

To a stirred solution of compound **4** (100 mg, 0.07 mmol), pyridine‐4‐boronic acid (11 mg, 0.09 mmol), and K_2_CO_3_ (30 mg, 0.22 mmol) in 1,4‐dioxane/water (4:1, 1 mL) under N_2_ was added Pd(PPh_3_)_4_ (10 mg, 8.65 µmol). The mixture was stirred for 16 h under reflux. After completion of the reaction, the mixture was filtered, and the organic layer of the filtrate was concentrated under reduced pressure and purified by silica gel column chromatography using petroleum ether/EtOAc (2:1) as eluent to give product **5** **g** (58 mg, 58%) as a light‐yellow oil. [α]^25^_D_‐143.0° (*c* 0.20, CH_2_Cl_2_). ^1^H‐NMR (400 MHz, CDCl_3_, 57:43 mixture of rotational isomers, *δ* ppm): 8.43–8.37 (m, 2H), 7.52–6.68 (m, 47.14H), 6.50 (d, *J* = 8.4 Hz, 0.43H), 6.32–6.20 (m, 2H), 6.14 (s, 0.43H), 5.25–4.38 (m, 18.43H), 4.22 (m, 0.57H), 4.04 (m, 0.43H), 3.80–3.68 (m, 1.57H), 3.19 (dd, *J* = 16.6, 5.3 Hz, 0.43H), 3.07 (dd, *J* = 16.2, 5.2 Hz, 0.57H), 2.65 (dd, *J* = 16.6, 9.2 Hz, 0.43H), 2.42 (dd, *J* = 16.2, 9.3 Hz, 0.57H). ^13^C‐NMR (100 MHz, CDCl3, 57:43 mixture of rotational isomers, *δ* ppm): 157.48, 157.37, 157.04, 155.69, 155.61, 155.60, 154.79, 154.75, 154.18, 154.05, 153.97, 152.77, 149.63, 149.20, 149.04, 148.99, 148.85, 148.24, 148.20, 148.03, 142.78, 142.49, 137.70, 137.41, 137.37, 137.32, 137.27, 137.23, 137.16, 137.13, 136.99, 136.95, 136.84, 136.50, 131.84, 131.73, 131.58, 130.51, 128.70–126.74 (CHBn), 120.87, 120.28, 120.20, 120.11, 115.11, 114.88, 114.81, 114.58, 113.89, 113.75, 113.59, 111.91, 110.18, 110.08, 110.05, 109.69, 103.33, 102.66, 92.60, 91.92, 91.88, 91.56, 81.87, 81.57, 81.50, 80.82, 73.59, 73.34, 71.55–70.01 (CH_2_Bn), 68.77, 68.41, 37.67, 37.54, 28.14, 28.06. HRMS (ESI) Calcd for C_91_H_78_NO_12_ [M+H]^+^ 1376.5524, found 1376.5513.

### Synthesis of 5,7,3′,4′‐Tetra‐O‐Benzyl‐8‐(Pyridin‐3‐yl)‐(+)‐Catechin‐(4α,8)‐[5,7,3′,4′‐Tetra‐O‐Benzyl‐(+)‐Catechin] (**5** **h**)

To a stirred solution of compound **4** (100 mg, 0.07 mmol), pyridine‐3‐boronic acid (11 mg, 0.09 mmol), and K_2_CO_3_ (30 mg, 0.22 mmol) in 1,4‐dioxane/water (4:1, 1 mL) under N_2_ was added Pd(PPh_3_)_4_ (10 mg, 8.65 µmol). The mixture was stirred for 16 h under reflux. After completion of the reaction, the mixture was filtered, and the organic layer of filtrate was concentrated under reduced pressure and purified by silica gel column chromatography using petroleum ether/EtOAc (2:1) as eluent to give product **5** **h** (47 mg, 47%) as a light‐yellow oil. [α]^25^_D_‐157.5° (*c* 0.20, CH_2_Cl_2_). ^1^H‐NMR (400 MHz, CDCl_3_, 53:47 mixture of rotational isomers, *δ* ppm): 8.51–8.47 (m, 1H), 8.32 (dd, *J* = 4.6, 1.6 Hz, 0.53H), 8.22 (dd, *J* = 4.8, 1.5 Hz, 0.47H), 7.51–6.69 (m, 47.06H), 6.48 (d, *J* = 8.4 Hz, 0.47H), 6.29 (s, 0.47H), 6.26–6.17 (m, 1.53H), 6.12 (s, 0.47H), 5.22–4.37 (m, 18.47H), 4.21 (m, 0.53H), 4.03 (m, 0.47H), 3.74–3.61 (m, 1.53H), 3.18 (dd, *J* = 16.3, 5.8 Hz, 0.47H), 3.04 (dd, *J* = 16.6, 5.3 Hz, 0.53H), 2.63 (dd, *J* = 16.3, 9.2 Hz, 0.47H), 2.41 (dd, *J* = 16.6, 9.1 Hz, 0.53H). ^13^C‐NMR (100 MHz, CDCl_3_, 53:47 mixture of rotational isomers, *δ* ppm): 157.09, 157.02, 156.99, 155.63, 155.57, 155.54, 154.87, 154.75, 154.29, 154.15, 153.94, 152.74, 152.16, 149.65, 149.17, 149.00, 148.98, 148.85, 148.78, 148.74, 147.91, 146.72, 146.64, 139.10, 139.05, 137.68, 137.43, 137.35, 137.33, 137.31, 137.29, 137.22, 137.20, 137.17, 137.15, 137.14, 137.10, 137.01, 136.92, 136.60, 131.84, 131.71, 130.50, 130.09, 129.89, 128.66–126.84 (CHBn), 122.21, 122.03, 120.81, 120.21, 120.18, 120.11, 115.32, 114.87, 114.75, 114.71, 114.52, 113.84, 113.62, 113.58, 111.99, 109.97, 109.61, 109.35, 109.17, 103.31, 102.60, 92.67, 91.86, 91.84, 91.58, 81.73, 81.46, 81.43, 80.79, 73.66, 73.35, 71.62–69.98 (CH_2_Bn), 68.78, 68.37, 37.71, 37.56, 28.09, 28.04. HRMS (ESI) Calcd for C_91_H_78_NO_12_ [M+H]^+^ 1376.5524, found 1376.5515.

### Synthesis of 8‐Methyl‐(+)‐Catechin‐(4α,8)‐(+)‐Catechin (**6a**)

Compound **5a** (200 mg, 0.15 mmol) was dissolved in MeOH (20 mL), and 20% Pd(OH)_2_/C (120 mg) was added with stirring. The reactor was sealed and purged with N_2_ and then with H_2_. The reactor was pressurized with H_2_ (15 psi), and stirring was started. After stirring for 6 h at room temperature, the reactor was vented and purged with N_2_. The reaction mixture was filtered through a cartridge (Nylon 6, 0.45 µm), the filtrate was concentrated under reduced pressure, and purified by octadecylsilyl silica gel column chromatography using water/MeOH (1:1) as eluent to give product **6a** (7 mg, 8%) as a yellow solid. [α]^25^_D_‐153.0° (*c* 0.10, CH_3_OH). ^1^H‐NMR (400 MHz, D_2_O, *δ* ppm): 6.92–6.83 (m, 2H), 6.79 (d, *J* = 8.2 Hz, 1H), 6.69 (dd, *J* = 8.2, 2.1 Hz, 1H), 6.55 (d, *J* = 2.1 Hz, 1H), 6.41 (dd, *J* = 8.2, 2.1 Hz, 1H), 6.13 (s, 1H), 6.00 (s, 1H), 4.51 (d, *J* = 7.7 Hz, 1H), 4.41–4.28 (m, 2H), 4.21 (m, 1H), 3.91 (m, 1H), 2.86 (dd, *J* = 15.9, 5.1 Hz, 1H), 2.46 (dd, *J* = 15.9, 8.3 Hz, 1H), 1.60 (s, 3H). ^13^C‐NMR (100 MHz, D_2_O, *δ* ppm): 154.97, 153.72, 153.05, 152.63, 151.98, 151.60, 144.24, 143.75, 143.71, 143.52, 131.09, 130.64, 120.28, 119.91, 116.16, 115.78, 114.85, 109.27, 107.07, 104.03, 101.32, 96.16, 95.28, 81.44, 80.38, 72.68, 66.88, 37.08, 27.32, 7.08. HRMS (ESI) Calcd for C_31_H_29_O_12_ [M+H]^+^ 593.1659, found 593.1647.

### Synthesis of 8‐Ethyl‐(+)‐Catechin‐(4α,8)‐(+)‐Catechin (**6b**)

Compound **5b** (485 mg, 0.37 mmol) was dissolved in MeOH (19 mL), and 20% Pd(OH)_2_/C (109 mg) was added with stirring. The reactor was sealed and purged with N_2_ and then with H_2_. The reactor was pressurized with H_2_ (15 psi), and stirring was started. After stirring for 4 h at room temperature, the reactor was vented and purged with N_2_. The reaction mixture was filtered through a cartridge (Nylon 6, 0.45 µm), and the filtrate was concentrated under reduced pressure and purified by octadecylsilyl silica gel column chromatography using water/MeOH (4:1) as eluent to give product **6b** (139 mg, 63%) as a light‐yellow solid. [α]^25^_D_−279.5° (*c* 1.00, CH_3_OH). ^1^H‐NMR (400 MHz, D_2_O, *δ* ppm): 6.93–6.82 (m, 2H), 6.75 (dd, *J* = 8.2, 2.0 Hz, 1H), 6.71 (d, *J* = 8.2 Hz, 1H), 6.48 (d, *J* = 2.0 Hz, 1H), 6.28 (dd, *J* = 8.2, 2.0 Hz, 1H), 5.98 (s, 1H), 4.62 (d, *J* = 7.1 Hz, 1H), 4.40–4.30 (m, 2H), 4.28 (m, 1H), 3.98 (m, 1H), 2.73 (dd, *J* = 16.2, 5.3 Hz, 1H), 2.47 (dd, *J* = 16.2, 7.6 Hz, 1H), 2.20 (m, 1H), 2.05 (m, 1H), 0.64 (t, *J* = 7.4 Hz, 3H). ^13^C‐NMR (100 MHz, D_2_O, *δ* ppm): 155.64, 154.31, 153.50, 153.37, 152.32, 152.22, 144.90, 144.49, 144.21, 131.95, 131.50, 120.98, 120.13, 116.84, 116.43, 116.37, 115.10, 111.53, 109.79, 107.89, 101.65, 82.15, 80.84, 73.32, 67.27, 37.78, 26.85, 16.26, 13.88. HRMS (ESI) Calcd for C_32_H_31_O_12_ [M+H]^+^ 607.1816, found 607.1810.

### Synthesis of 8‐Isopropyl‐(+)‐Catechin‐(4α,8)‐(+)‐Catechin (**6c**)

Compound **5c** (200 mg, 0.15 mmol) was dissolved in MeOH (20 mL), and 20% Pd(OH)_2_/C (120 mg) was added with stirring. The reactor was sealed and purged with N_2_ and then with H_2_. The reactor was pressurized with H_2_ (15 psi), and stirring was started. After stirring for 2 h at room temperature, the reactor was vented and purged with N_2_. The reaction mixture was filtered through a cartridge (Nylon 6, 0.45 µm), and the filtrate was concentrated under reduced pressure and purified by octadecylsilyl silica gel column chromatography using water/MeOH (1:1) as eluent to give product **6c** (28 mg, 30%) as a light‐yellow solid. [α]^25^_D_‐227.0° (*c* 0.10, CH_3_OH). ^1^H‐NMR (400 MHz, D_2_O, *δ* ppm): 6.98 (d, *J* = 2.0 Hz, 1H), 6.89 (d, *J* = 8.2 Hz, 1H), 6.82 (dd, *J* = 8.2, 2.0 Hz, 1H), 6.67 (d, *J* = 8.2 Hz, 1H), 6.44 (d, *J* = 2.0 Hz, 1H), 6.13 (dd, *J* = 8.2, 2.0 Hz, 1H), 5.99 (s, 1H), 4.72 (d, *J* = 7.0 Hz, 1H), 4.46–4.31 (m, 3H), 4.11 (m, 1H), 2.98 (m, 1H), 2.66–2.41 (m, 2H), 0.90 (d, *J* = 7.3 Hz, 3H), 0.80 (d, *J* = 7.3 Hz, 3H). ^13^C‐NMR (100 MHz, D_2_O, *δ* ppm): 156.16, 153.49, 152.80, 152.57, 151.62, 151.40, 144.00, 143.78, 143.73, 143.33, 131.53, 131.11, 120.30, 118.73, 116.00, 115.76, 115.53, 114.61, 113.91, 108.87, 107.68, 100.50, 95.15, 81.50, 79.84, 72.74, 66.34, 37.24, 24.72, 23.75, 20.12, 19.84. HRMS (ESI) Calcd for C_33_H_33_O_12_ [M+H]^+^ 621.1972, found 621.1962.

### Synthesis of 8‐Propyl‐(+)‐Catechin‐(4α,8)‐(+)‐Catechin (**6d**)

Compound **5d** (200 mg, 0.15 mmol) was dissolved in MeOH (20 mL), and 20% Pd(OH)_2_/C (120 mg) was added with stirring. The reactor was sealed and purged with N_2_ and then with H_2_. The reactor was pressurized with H_2_ (15 psi), and stirring was started. After stirring for 2 h at room temperature, the reactor was vented and purged with N_2_. The reaction mixture was filtered through a cartridge (Nylon 6, 0.45 µm), and the filtrate was concentrated under reduced pressure and purified by octadecylsilyl silica gel column chromatography using water/MeOH (1:1) as eluent to give product **6d** (11 mg, 12%) as a yellow solid. [α]^25^_D_‐210.0° (*c* 0.10, CH_3_OH). ^1^H‐NMR (400 MHz, D_2_O, *δ* ppm): 6.91 (d, *J* = 2.1 Hz, 1H), 6.89 (d, *J* = 8.1 Hz, 1H), 6.76 (dd, *J* = 8.2, 2.2 Hz, 1H), 6.71 (d, *J* = 8.2 Hz, 1H), 6.49 (d, *J* = 2.2 Hz, 1H), 6.27 (dd, *J* = 8.1, 2.1 Hz, 1H), 6.13 (s, 1H), 6.00 (s, 1H), 4.66 (d, *J* = 7.0 Hz, 1H), 4.41–4.23 (m, 3H), 4.01 (m, 1H), 2.71 (dd, *J* = 16.3, 5.3 Hz, 1H), 2.48 (dd, *J* = 16.3, 7.4 Hz, 1H), 2.23–1.98 (m, 2H), 1.07 (m, 2H), 0.61 (t, *J* = 7.4 Hz, 3H). ^13^C‐NMR (100 MHz, D_2_O, *δ* ppm): 155.24, 153.64, 152.79, 152.71, 151.88, 151.67, 144.16, 143.76, 143.53, 143.51, 131.29, 130.81, 120.33, 119.37, 116.12, 115.70, 115.68, 114.35, 109.37, 109.06, 107.09, 100.87, 96.33, 81.44, 80.10, 72.66, 66.51, 37.18, 25.93, 24.04, 22.26, 13.07. HRMS (ESI) Calcd for C_33_H_33_O_12_ [M+H]^+^ 621.1972, found 621.1960.

### Synthesis of 8‐Isobutyl‐(+)‐Catechin‐(4α,8)‐(+)‐Catechin (**6e**)

Compound **5e** (200 mg, 0.15 mmol) was dissolved in MeOH (20 mL), and 20% Pd(OH)_2_/C (120 mg) was added with stirring. The reactor was sealed and purged with N_2_ and then with H_2_. The reactor was pressurized with H_2_ (15 psi), and stirring was started. After stirring for 2 h at room temperature, the reactor was vented and purged with N_2_. The reaction mixture was filtered through a cartridge (Nylon 6, 0.45 µm), and the filtrate was concentrated under reduced pressure and purified by octadecylsilyl silica gel column chromatography using water/MeOH (2:3) as eluent to give product **6e** (23 mg, 24%) as a yellow solid. [α]^25^_D_‐221.0° (*c* 0.10, CH_3_OH). ^1^H‐NMR (400 MHz, CD_3_OD, 3:2 mixture of rotational isomers, *δ* ppm): 6.98–6.77 (m, 2H), 6.75 (dd, *J* = 8.1, 1.5 Hz, 0.6H), 6.68 (d, *J* = 8.2 Hz, 0.6H), 6.62–6.55 (m, 1.6H), 6.47 (d, *J* = 2.2 Hz, 0.6H), 6.11 (dd, *J* = 8.2, 2.2 Hz, 0.6H), 6.05 (s, 0.6H), 5.93–5.89 (m, 1H), 5.84 (s, 0.4H), 4.74 (d, *J* = 7.2 Hz, 0.4H), 4.63 (d, *J* = 6.3 Hz, 0.6H), 4.50 (d, *J* = 7.8 Hz, 0.4H), 4.46–4.33 (m, 1.6H), 4.27 (m, 0.4H), 4.20 (m, 0.6H), 4.07 (m, 0.4H), 3.87 (m, 0.6H), 2.79 (dd, *J* = 16.3, 5.5 Hz, 0.4H), 2.66–2.16 (m, 3.6H), 1.79 (m, 0.4H), 1.59 (m, 0.6H), 0.85 (d, *J* = 6.6 Hz, 1.2H), 0.79 (d, *J* = 6.6 Hz, 1.2H), 0.69 (d, *J* = 6.6 Hz, 1.8H), 0.60 (d, *J* = 6.6 Hz, 1.8H). ^13^C‐NMR (100 MHz, CD_3_OD, 3:2 mixture of rotational isomers, *δ* ppm): 156.86, 155.87, 155.85, 155.70, 155.42, 155.22, 155.01, 154.68, 154.60, 154.35, 153.99, 146.11, 146.05, 145.91, 145.76, 145.51, 145.34, 133.14, 132.93, 132.05, 131.90, 120.76, 120.56, 120.11, 119.32, 116.39, 116.16, 116.03, 115.86, 115.79, 115.14, 114.90, 109.61, 109.09, 108.72, 108.20 106.90, 106.62, 101.92, 100.38, 97.59, 97.26, 97.00, 95.86, 83.80, 83.60, 82.94, 81.97, 74.37, 73.86, 68.46, 38.93, 38.74, 32.66, 29.94, 29.91, 28.33, 27.53, 23.11, 23.01, 22.99, 22.76. HRMS (ESI) Calcd for C_34_H_35_O_12_ [M+H]^+^ 635.2129, found 635.2120.

### Synthesis of 8‐Phenyl‐(+)‐Catechin‐(4α,8)‐(+)‐Catechin (**6f**)

Compound **5f** (80 mg, 0.06 mmol) was dissolved in MeOH (20 mL), and 20% Pd(OH)_2_/C (80 mg) was added with stirring. The reactor was sealed and purged with N_2_ and then with H_2_. The reactor was pressurized with H_2_ (15 psi), and stirring was started. After stirring for 4 h at room temperature, the reactor was vented and purged with N_2_. The reaction mixture was filtered through a cartridge (Nylon 6, 0.45 µm), and the filtrate was concentrated under reduced pressure and purified by octadecylsilyl silica gel column chromatography using water/MeOH (2:3) as eluent to give product **6f** (8 mg, 21%) as a yellow solid. [α]^25^_D_‐198.0° (*c* 0.10, CH_3_OH). ^1^H‐NMR (400 MHz, D_2_O, *δ* ppm): 7.26–7.12 (m, 3H), 6.82–6.70 (m, 5H), 6.63 (d, *J* = 8.0 Hz, 1H), 6.60 (d, *J* = 2.0 Hz, 1H), 6.39 (dd, *J* = 8.0, 2.0 Hz, 1H), 6.11 (s, 1H), 4.62 (d, *J* = 7.2 Hz, 1H), 4.47–4.26 (m, 3H), 4.01 (m, 1H), 2.78 (dd, *J* = 16.0, 5.4 Hz, 1H), 2.48 (dd, *J* = 16.0, 7.9 Hz, 1H). ^13^C‐NMR (100 MHz, D_2_O, *δ* ppm): 153.77, 153.64, 152.92, 152.87, 151.69, 151.50, 144.31, 143.85, 143.67, 143.30, 133.31, 131.04, 130.88, 130.64, 127.81, 127.16, 126.72, 119.97, 119.39, 115.91, 115.85, 115.36, 114.79, 109.78, 108.78, 106.94, 100.82, 81.53, 80.32, 72.36, 66.82, 37.03, 26.26. HRMS (ESI) Calcd for C_36_H_31_O_12_ [M+H]^+^ 655.1816, found 655.1804.

### Synthesis of 8‐(Pyridin‐4‐yl)‐(+)‐Catechin‐(4α,8)‐(+)‐Catechin (**6** **g**)

Compound **5** **g** (73 mg, 0.05 mmol) was dissolved in MeOH (10 mL), and 20% Pd(OH)_2_/C (17 mg) was added with stirring. The reactor was sealed and purged with N_2_ and then with H_2_. The reactor was pressurized with H_2_ (15 psi), and stirring was started. After stirring for 4 h at room temperature, the reactor was vented and purged with N_2_. The reaction mixture was filtered through a cartridge (Nylon 6, 0.45 µm), and the filtrate was concentrated under reduced pressure and purified by octadecylsilyl silica gel column chromatography using water/MeOH (4:1) as eluent to give product **6** **g** (16 mg, 46%) as a yellow solid. [α]^25^_D_‐130.0° (*c* 0.01, CH_3_OH). ^1^H‐NMR (400 MHz, D_2_O, *δ* ppm): 8.05 (m, 2H), 6.75–6.61 (m, 4H), 6.57 (dd, *J* = 8.2, 2.1 Hz, 1H), 6.51 (d, *J* = 8.2 Hz, 1H), 6.48 (d, *J* = 2.1 Hz, 1H), 6.34 (dd, *J* = 8.2, 2.1 Hz, 1H), 4.45 (d, *J* = 7.8 Hz, 1H), 4.32–4.19 (m, 2H), 4.09 (m, 1H), 3.83 (m, 1H), 2.74 (dd, *J* = 15.8, 5.2 Hz, 1H), 2.36 (dd, *J* = 15.8, 7.3 Hz, 1H). ^13^C‐NMR (100 MHz, D_2_O, *δ* ppm): 156.27, 156.00, 155.53, 154.77, 153.98, 151.69, 145.00, 144.31, 144.22, 143.77, 133.30, 132.89, 132.02, 131.63, 123.06, 120.11, 119.39, 116.98, 116.86, 116.55, 115.07, 110.42, 107.52, 106.62, 102.11, 82.71, 81.15, 72.91, 67.07, 37.08, 26.05. HRMS (ESI) Calcd for C_35_H_30_NO_12_ [M+H]^+^ 656.1768, found 656.1755.

### Synthesis of 8‐(Pyridin‐3‐yl)‐(+)‐Catechin‐(4α,8)‐(+)‐Catechin (**6** **h**)

Compound **5** **h** (92 mg, 0.07 mmol) was dissolved in MeOH (10 mL), and 20% Pd(OH)_2_/C (21 mg) was added with stirring. The reactor was sealed and purged with N_2_ and then with H_2_. The reactor was pressurized with H_2_ (15 psi), and stirring was started. After stirring for 4 h at room temperature, the reactor was vented and purged with N_2_. The reaction mixture was filtered through a cartridge (Nylon 6, 0.45 µm), and the filtrate was concentrated under reduced pressure and purified by octadecylsilyl silica gel column chromatography using water/MeOH (4:1) as eluent to give product **6** **h** (19 mg, 43%) as a yellow solid. [α]^25^_D_‐150.0° (*c* 0.01, CH_3_OH). ^1^H‐NMR (400 MHz, D_2_O, *δ* ppm): 8.03 (m, 1H), 7.67 (m, 1H), 7.14–7.00 (m, 2H), 6.70–6.62 (m, 2H), 6.57 (dd, *J* = 8.4, 2.0 Hz, 1H), 6.50 (d, *J* = 8.4 Hz, 1H), 6.43 (d, *J* = 2.0 Hz, 1H), 6.31 (dd, *J* = 8.4, 2.0 Hz, 1H), 4.46 (d, *J* = 7.3 Hz, 1H), 4.34–4.22 (m, 2H), 4.09 (m, 1H), 3.82 (m, 1H), 2.71 (dd, *J* = 16.1, 5.3 Hz, 1H), 2.35 (dd, *J* = 16.1, 8.4 Hz, 1H).^13^C‐NMR (100 MHz, D_2_O, *δ* ppm): 155.96, 154.65, 153.90, 152.95, 152.71, 151.93, 149.86, 149.83, 145.33, 144.37, 144.14, 143.73, 133.48, 132.92, 132.00, 131.67, 123.41, 120.08, 119.77, 115.97, 115.87, 115.24, 114.81, 108.89, 107.09, 105.60, 100.98, 81.49, 80.45, 73.09, 67.19, 37.04, 26.67. HRMS (ESI) Calcd for C_35_H_30_NO_12_ [M+H]^+^ 656.1768, found 656.1756.

### Synthesis of 3‐O‐Acetyl‐(+)‐Catechin‐(4α,8)‐[3‐O‐Acetyl‐(+)‐Catechin] (**8**)

Compound **7** (20 mg, 13.69 µmol) was dissolved in THF/MeOH (1:2, 3 mL), NaHCO_3_ (3.5 mg, 41.66 µmol), and 20% Pd(OH)_2_/C (4 mg) were added with stirring. The reactor was sealed and purged with N_2_ and then with H_2_. The reactor was pressurized with H_2_ (15 psi), and stirring was started. After stirring for 6 h at room temperature, the reactor was vented and purged with N_2_. AcOH (1.7 mg, 28.31 µmol) was added and followed by stirring for 10 min. The reaction mixture was filtered through a cartridge (Nylon 6, 0.45 µm), the filtrate was concentrated under reduced pressure, and purified by octadecylsilyl silica gel column chromatography using water/MeOH (2:3) as eluent to give product **8** (7 mg, 77%) as a yellow solid. [α]^25^_D_‐204.0° (*c* 0.60, CH_3_OH). ^1^H‐NMR (400 MHz, D_2_O, *δ* ppm): 6.88–6.70 (m, 3H), 6.65–6.52 (m, 2H), 6.29 (dd, *J* = 7.3, 2.1 Hz, 1H), 6.10 (s, 1H), 6.04 (d, *J* = 2.6 Hz, 1H), 5.80 (d, *J* = 2.6 Hz, 1H), 5.69 (m, 1H), 5.15 (m, 1H), 4.98 (d, *J* = 6.5 Hz, 1H), 4.57 (d, *J* = 9.9 Hz, 1H), 4.53 (d, *J* = 8.3 Hz, 1H), 2.73 (dd, *J* = 16.5, 5.4 Hz, 1H), 2.63 (dd, *J* = 16.5, 6.7 Hz, 1H), 1.95 (s, 3H), 1.73 (s, 3H). ^13^C‐NMR (100 MHz, D_2_O, *δ* ppm): 173.36, 172.56, 156.32, 155.06, 153.49, 153.09, 152.65, 144.50, 143.79, 143.65, 130.00, 129.40, 120.00, 118.97, 115.94, 115.60, 115.40, 114.11, 107.73, 106.17, 100.14, 97.00, 95.44, 95.30, 79.21, 77.18, 73.97, 69.60, 34.53, 23.31, 20.36, 19.83. HRMS (ESI) Calcd for C_34_H_31_O_14_ [M+H]^+^ 663.1714, found 663.1704.

### Synthesis of 5,7,3′,4′‐Tetra‐O‐Methyl‐(+)‐Catechin‐(4α, 8)‐[5,7,3′,4′‐Tetra‐O‐Methyl‐(+)‐Catechin] (**9**)

Procyanidin B3 (**1**, 100 mg, 0.17 mmol) was dissolved in DMF (2 mL), and Cs_2_CO_3_ (900 mg, 2.76 mmol) and dimethyl sulfate (262 mg, 2.07 mmol) were added. The mixture was stirred for 2 h at 100 °C. After completion of the reaction, EtOAc and water were added, and the organic layer was concentrated under reduced pressure and purified by silica gel column chromatography using petroleum ether/EtOAc (1:2) as eluent to give product **9** (53 mg, 44%) as a white solid. ^1^H‐NMR (400 MHz, CDCl_3_, 2:1 mixture of rotational isomers, *δ* ppm): 7.08–6.98 (m, 1.33H), 6.92– 6.69 (m, 3.33H), 6.59–6.47 (m, 1.34H), 6.22 (s, 0.67H), 6.14 (d, *J* = 2.2 Hz, 0.33H), 6.06 (s, 0.33H), 6.03 (d, *J* = 2.2 Hz, 0.67H), 5.98 (d, *J* = 2.2 Hz, 0.33H), 5.95 (d, *J* = 2.2 Hz, 0.67H), 4.83 (d, *J* = 7.1 Hz, 0.33H), 4.68–4.61 (m, 1H), 4.55–4.42 (m, 1.67H), 4.36 (m, 0.33H), 4.19 (m, 0.67H), 4.03 (m, 0.33H), 3.95–3.39 (m, 24.67H), 3.05 (dd, *J* = 16.5, 6.1 Hz, 0.67H), 2.95 (dd, *J* = 16.5, 5.7 Hz, 0.33H), 2.67 (dd, *J* = 16.5, 8.8 Hz, 0.33H), 2.53 (dd, *J* = 16.5, 9.7 Hz, 0.67H). HRMS (ESI) Calcd for C_38_H_43_O_12_ [M+H]^+^ 691.2755, found 691.2741.

### Synthesis of 3′‐O‐Methyl‐(+)‐Catechin (**10a**) and 4′‐O‐Methyl‐(+)‐Catechin (**10b**)

(+)‐Catechin (**3**, 40.0 g, 0.14 mol) was dissolved in acetone (2 L), K_2_CO_3_ (115.2 g, 0.83 mol), 4A molecular sieve (40.0 g) and CH_3_I (364.8 g, 2.57 mol) were added under N_2_. The mixture was stirred for 48 h at room temperature in the dark. After completion of the reaction, the mixture was filtered, and the filtrate was concentrated under reduced pressure and purified by silica gel column chromatography using DCM/MeOH (30:1 to 10:1) as eluent to give the crude product. This crude product was further purified by octadecylsilyl silica gel column chromatography using MeOH/0.1% FA aqueous solution (3:7) as eluent to give product **10a** (3329 mg, 8%) as an off‐white solid and **10b** (3460 mg, 8%) as an off‐white solid. **10a**: ^1^H‐NMR (400 MHz, DMSO‐*d*_6_, *δ* ppm): 6.90 (d, *J* = 1.5 Hz, 1H), 6.78–6.69 (m, 2H), 5.89 (d, *J* = 2.0 Hz, 1H), 5.69 (d, *J* = 2.0 Hz, 1H), 4.51 (d, *J* = 7.9 Hz, 1H), 3.89 (m, 1H), 3.75 (s, 3H), 2.72 (dd, *J* = 16.0, 5.4 Hz, 1H), 2.35 (dd, *J* = 16.0, 8.5 Hz, 1H). HRMS (ESI) Calcd for C_16_H_17_O_6_ [M+H]^+^ 305.1025, found 305.1015. **10b**: ^1^H‐NMR (400 MHz, DMSO‐*d*_6_, *δ* ppm): 6.87 (d, *J* = 8.2 Hz, 1H), 6.77–6.71 (m, 2H), 5.89 (d, *J* = 1.8 Hz, 1H), 5.69 (d, *J* = 1.8 Hz, 1H), 4.52 (d, *J* = 7.4 Hz, 1H), 3.82 (m, 1H), 3.74 (s, 3H), 2.65 (dd, *J* = 16.0, 4.8 Hz, 1H), 2.35 (dd, *J* = 16.0, 7.9 Hz, 1H). HRMS (ESI) Calcd for C_16_H_17_O_6_ [M+H]^+^ 305.1025, found 305.1017.

### Synthesis of 5,7,4′‐Tri‐O‐Benzyl‐3′‐O‐Methyl‐(+)‐Catechin (**11a**)

To a stirred solution of compound **10a** (875 mg, 2.87 mmol) in DMF (8.8 mL), K_2_CO_3_ (2765 mg, 20.14 mmol) and BnBr (2214 mg, 12.95 mmol) were slowly added. The mixture was stirred for 48 h at room temperature. After completion of the reaction, DCM and water were added, and the organic layer was concentrated under reduced pressure and purified by silica gel column chromatography using petroleum ether/EtOAc (5:1 to 2:1) as eluent to give product **11a** (1477 mg, 89%) as an off‐white solid. ^1^H‐NMR (400 MHz, CDCl_3_, *δ* ppm): 7.49–7.29 (m, 15H), 7.03–6.90 (m, 3H), 6.28 (d, *J* = 1.9 Hz, 1H), 6.23 (d, *J* = 1.9 Hz, 1H), 5.18 (s, 2H), 5.04 (s, 2H), 4.99 (s, 2H), 4.65 (d, *J* = 8.1 Hz, 1H), 4.10 (m, 1H), 3.90 (s, 3H), 3.19 (dd, *J* = 16.3, 5.6 Hz, 1H), 2.67 (dd, *J* = 16.3, 8.1 Hz, 1H). HRMS (ESI) Calcd for C_37_H_35_O_6_ [M+H]^+^ 575.2434, found 575.2424.

### Synthesis of 5,7,3′‐Tri‐O‐Benzyl‐4′‐O‐Methyl‐(+)‐Catechin (**11b**)

To a stirred solution of compound **10b** (300 mg, 0.98 mmol) in DMF (3 mL), K_2_CO_3_ (948 mg, 6.86 mmol) and BnBr (754 mg, 4.41 mmol) were slowly added. The mixture was stirred for 48 h at room temperature. After completion of the reaction, DCM and water were added, and the organic layer was concentrated under reduced pressure and purified by silica gel column chromatography using petroleum ether/EtOAc (5:1 to 2:1) as eluent to give product **11b** (315 mg, 56%) as an off‐white solid. ^1^H‐NMR (400 MHz, CDCl_3_, *δ* ppm): 7.49–7.29 (m, 15H), 7.05–6.88 (m, 3H), 6.28 (d, *J* = 1.8 Hz, 1H), 6.21 (d, *J* = 1.8 Hz, 1H), 5.16 (s, 2H), 5.03 (s, 2H), 5.00 (s, 2H), 4.63 (d, *J* = 8.2 Hz, 1H), 3.99 (m, 1H), 3.90 (s, 3H), 3.11 (dd, *J* = 16.1, 5.6 Hz, 1H), 2.65 (dd, *J* = 16.1, 8.9 Hz, 1H). HRMS (ESI) Calcd for C_37_H_35_O_6_ [M+H]^+^ 575.2434, found 575.2425.

### Synthesis of 5,7,4′‐Tri‐O‐Benzyl‐4β‐(2‐Ethoxyethoxy)‐3′‐O‐Methyl‐(+)‐Catechin (**12a**)

To a stirred solution of compound **11a** (600 mg, 1.04 mmol) and 2‐ethoxyethanol (1131 mg, 12.55 mmol) in DCM (6 mL) under N_2_ was added DDQ (474 mg, 2.09 mmol) slowly. After completion of the addition, the reaction mixture was stirred for 3 h at room temperature. After completion of the reaction, DMAP (357 mg, 2.92 mmol) was added and further stirred for 10 min. The mixture was filtered through a pad of Celite, and the filtrate was washed with water. The organic layer was concentrated under reduced pressure and purified by silica gel column chromatography using petroleum ether/EtOAc (3:1) as eluent to give product **12a** (307 mg, 44%) as a light‐yellow solid. [α]^25^_D_+61.0° (*c* 0.10, CH_2_Cl_2_). ^1^H‐NMR (400 MHz, CDCl_3_, *δ* ppm): 7.46–7.31 (m, 15H), 7.02 (d, *J* = 1.8 Hz, 1H), 6.96 (dd, *J* = 8.3, 1.8 Hz, 1H), 6.90 (d, *J* = 8.3 Hz, 1H), 6.26 (d, *J* = 2.0 Hz, 1H), 6.16 (d, *J* = 2.0 Hz, 1H), 5.17 (s, 2H), 5.04 (m, 2H), 4.98 (s, 2H), 4.91 (d, *J* = 10.6 Hz, 1H), 4.78 (d, *J* = 3.7 Hz, 1H), 4.00–3.79 (m, 6H), 3.57–3.43 (m, 4H), 1.17 (t, *J* = 6.9 Hz, 3H). ^13^C‐NMR (100 MHz, CDCl_3_, *δ* ppm): 160.98, 158.76, 156.26, 149.80, 148.58, 137.44, 136.79, 136.71, 131.19, 128.90–127.37 (CHBn), 120.79, 113.92, 111.52, 104.48, 94.48, 93.79, 71.62, 71.31, 71.17, 70.98, 70.65, 70.56, 70.23, 70.17, 66.71, 56.15, 15.18. HRMS (ESI) Calcd for C_41_H_43_O_8_ [M+H]^+^ 663.2958, found 663.2961.

### Synthesis of 5,7,3′‐Tri‐O‐Benzyl‐4β‐(2‐Ethoxyethoxy)‐4′‐O‐Methyl‐(+)‐Catechin (**12b**)

To a stirred solution of compound **11b** (200 mg, 0.35 mmol) and 2‐ethoxyethanol (377 mg, 4.18 mmol) in DCM (2 mL) under N_2_ was added DDQ (158 mg, 0.70 mmol) slowly. After completion of the addition, the reaction mixture was stirred for 3 h at room temperature. After completion of the reaction, DMAP (119 mg, 0.97 mmol) was added and further stirred for 10 min. The mixture was filtered through a pad of Celite, and the filtrate was washed with water. The organic layer was concentrated under reduced pressure and purified by silica gel column chromatography using petroleum ether/EtOAc (3:1) as eluent to give product **12b** (123 mg, 53%) as a light‐yellow solid. [α]^25^_D_‐34.3° (*c* 1.00, CH_2_Cl_2_). ^1^H‐NMR (400 MHz, CDCl_3_, *δ* ppm): 7.47–7.30 (m, 15H), 7.10–7.03 (m, 2H), 6.93 (d, *J* = 8.0 Hz, 1H), 6.26 (d, *J* = 1.9 Hz, 1H), 6.15 (d, *J* = 1.9 Hz, 1H), 5.13 (s, 2H), 5.05 (m, 2H), 4.98 (s, 2H), 4.89 (d, *J* = 10.3 Hz, 1H), 4.77 (d, *J* = 3.0 Hz, 1H), 3.97–3.82 (m, 6H), 3.60–3.44 (m, 4H), 1.17 (t, *J* = 7.0 Hz, 3H). ^13^C‐NMR (100 MHz, CDCl_3_, *δ* ppm): 160.91, 158.69, 156.20, 149.99, 148.45, 137.25, 136.75, 136.66, 131.21, 128.69–127.59 (CHBn), 121.36, 113.65, 111.82, 104.40, 94.41, 93.70, 71.68, 71.59, 71.28, 71.22, 70.93, 70.48, 70.16, 70.12, 66.65, 56.18, 15.15. HRMS (ESI) Calcd for C_41_H_43_O_8_ [M+H]^+^ 663.2958, found 663.2956.

### Synthesis of 3′‐O‐Methyl‐(+)‐Catechin‐(4α,8)‐(+)‐Catechin (**13a**)

Compound **12a** (200 mg, 0.30 mmol), DMAP (1.8 mg, 0.01 mmol), and triethylamine (TEA, 76 mg, 0.75 mmol) were dissolved in DCM (2 mL), and Ac_2_O (37 mg, 0.36 mmol) was added with stirring at 5 °C–10 °C. After completion of the addition, the reaction mixture was stirred for 3 h at room temperature. After completion of the reaction, the mixture was washed with water and adjusted to pH 6 with 10% aqueous HCl. The organic layer was washed with saturated aqueous NaCl, dried over anhydrous Na_2_SO_4,_ and concentrated to give the crude intermediate 1. Then, intermediate 1 was dissolved in DCM (2 mL), and NBS (55 mg, 0.31 mmol) was added at 0 °C under N_2_. After stirring for 3 h at room temperature, the mixture was washed with 3% aqueous Na_2_S_2_O_3_. The organic layer was dried over anhydrous Na_2_SO_4_ and concentrated to give the crude intermediate 2. The intermediate 2 and compound **11c** (195 mg, 0.30 mmol) were dissolved in DCM (2.4 mL), and TMSOTf (67 mg, 0.30 mmol) was added with stirring at −60 °C. The reaction mixture was stirred for 0.5 h. After completion of the reaction, the mixture was warmed to 0 °C, and saturated aqueous NaHCO_3_ (1.2 mL) was added, followed by stirring for 10 min. The mixture was washed with saturated aqueous NaCl (1.2 mL), and the organic layer was concentrated under reduced pressure to give the crude intermediate 3. The intermediate 3 was dissolved in THF/MeOH (2:3, 4 mL), and NaOH (48 mg, 1.20 mmol) was slowly added. After stirring for 4 h under reflux, the mixture was cooled and concentrated to give a semisolid. The semisolid was dissolved in EtOAc (2 mL) and washed with saturated aqueous NaCl (2 mL), and the organic layer was concentrated to give crude intermediate 4. The intermediate 4 (390 mg in theoretical amount) was dissolved in THF/MeOH (1:2, 3 mL). Aqueous NaHCO_3_ (76 mg, 0.90 mmol) and 20% Pd(OH)_2_/C (78 mg) were added with stirring. The reactor was sealed and purged with N_2_ and then with H_2_. The reactor was pressurized with H_2_ (15 psi), and stirring was started. After stirring for 6 h at room temperature, the reactor was vented and purged with N_2_. AcOH (38 mg, 0.63 mmol) was added and followed by stirring for 10 min. The reaction mixture was filtered through a cartridge (Nylon 6, 0.45 µm), the filtrate was concentrated under reduced pressure, and purified by octadecylsilyl silica gel column chromatography using water/MeOH (3:2) as eluent to give product **13a** (43 mg, 24% yield for 5 steps) as a yellow solid. [α]^25^_D_‐156.0° (*c* 0.10, CH_3_OH). ^1^H‐NMR (400 MHz, D_2_O, *δ* ppm): 6.87–6.76 (m, 2H), 6.72–6.66 (m, 2H), 6.58 (d, *J* = 1.8 Hz, 1H), 6.44 (dd, *J* = 8.0, 1.8 Hz, 1H), 4.51 (d, *J* = 8.4 Hz, 1H), 4.47–4.21 (m, 3H), 3.79 (m, 1H), 3.72 (s, 3H), 2.88 (dd, *J* = 15.7, 5.7 Hz, 1H), 2.47 (dd, *J* = 15.7, 9.2 Hz, 1H). ^13^C‐NMR (100 MHz, D_2_O, *δ* ppm): 156.56, 155.15, 154.56, 154.26, 153.13, 152.99, 147.45, 145.38, 143.96, 143.72, 130.61, 130.34, 121.35, 119.76, 115.58, 115.29, 114.95, 110.95, 108.81, 107.02, 101.38, 82.03, 80.51, 72.43, 67.53, 55.94, 36.89, 27.89. HRMS (ESI) Calcd for C_31_H_29_O_12_ [M+H]^+^ 593.1659, found 593.1649.

### Synthesis of 4′‐O‐Methyl‐(+)‐Catechin‐(4α,8)‐(+)‐Catechin (**13b**)

Compound **12b** (200 mg, 0.30 mmol), DMAP (1.8 mg, 0.01 mmol), and TEA (76 mg, 0.75 mmol) were dissolved in DCM (2 mL), and Ac_2_O (37 mg, 0.36 mmol) was added with stirring at 5 °C–10 °C. After completion of the addition, the reaction mixture was stirred for 3 h at room temperature. After completion of the reaction, the mixture was washed with water and adjusted to pH 6 with 10% aqueous HCl. The organic layer was washed with saturated aqueous NaCl, dried over anhydrous Na_2_SO_4,_ and concentrated to give the crude intermediate 1. Then, intermediate 1 was dissolved in DCM (2 mL), and NBS (55 mg, 0.31 mmol) was added at 0 °C under N_2_. After stirring for 3 h at room temperature, the mixture was washed with 3% aqueous Na_2_S_2_O_3_. The organic layer was dried over anhydrous Na_2_SO_4_ and concentrated to give the crude intermediate 2. The intermediate 2 and compound **11c** (195 mg, 0.30 mmol) were dissolved in DCM (2.4 mL), and TMSOTf (67 mg, 0.30 mmol) was added with stirring at −60 °C. The reaction mixture was stirred for 0.5 h. After completion of the reaction, the mixture was warmed to 0 °C, and saturated aqueous NaHCO_3_ (1.2 mL) was added, followed by stirring for 10 min. The mixture was washed with saturated aqueous NaCl (1.2 mL), and the organic layer was concentrated under reduced pressure to give the crude intermediate 3. The intermediate 3 was dissolved in THF/MeOH (2:3, 4 mL), and NaOH (48 mg, 1.20 mmol) was slowly added. After stirring for 4 h under reflux, the mixture was cooled and concentrated to give a semisolid. The semisolid was dissolved in EtOAc (2 mL) and washed with saturated aqueous NaCl (2 mL), and the organic layer was concentrated to give crude intermediate 4. The intermediate 4 (390 mg in theoretical amount) was dissolved in THF/MeOH (1:2, 3 mL). Aqueous NaHCO_3_ (76 mg, 0.90 mmol) and 20% Pd(OH)_2_/C (78 mg) were added with stirring. The reactor was sealed and purged with N_2_ and then with H_2_. The reactor was pressurized with H_2_ (15 psi), and stirring was started. After stirring for 6 h at room temperature, the reactor was vented and purged with N_2_. AcOH (38 mg, 0.63 mmol) was added and followed by stirring for 10 min. The reaction mixture was filtered through a cartridge (Nylon 6, 0.45 µm), the filtrate was concentrated under reduced pressure, and purified by octadecylsilyl silica gel column chromatography using water/MeOH (3:2) as eluent to give product **13b** (25 mg, 14% yield for 5 steps) as a yellow solid. [α]^25^_D_‐208.0° (*c* 0.10, CH_3_OH). ^1^H‐NMR (400 MHz, D_2_O, *δ* ppm): 6.87–6.75 (m, 2H), 6.72 (d, *J* = 1.8 Hz, 1H), 6.64–6.54 (m, 2H), 6.41 (dd, *J* = 7.9, 1.8 Hz, 1H), 4.48 (d, *J* = 7.9 Hz, 1H), 4.36–4.22 (m, 3H), 3.83–3.70 (m, 4H), 2.78 (dd, *J* = 15.9, 5.7 Hz, 1H), 2.41 (dd, *J* = 15.9, 8.7 Hz, 1H). ^13^C‐NMR (100 MHz, D_2_O, *δ* ppm): 156.55, 154.99, 154.43, 154.01, 152.98, 152.89, 147.69, 144.68, 143.96, 143.74, 130.98, 130.55, 120.22, 119.93, 115.57, 114.99, 114.82, 112.23, 108.86, 106.95, 101.24, 95.17, 81.77, 80.39, 72.62, 67.03, 55.81, 36.84, 27.33. HRMS (ESI) Calcd for C_31_H_29_O_12_ [M+H]^+^ 593.1659, found 593.1648.

### Synthesis of (+)‐Catechin‐(4α,8)‐[3′‐O‐Methyl‐(+)‐Catechin] (**13c**)

Compound **12c** (200 mg, 0.27 mmol), DMAP (1.7 mg, 0.01 mmol), and TEA (69 mg, 0.68 mmol) were dissolved in DCM (2 mL), and Ac_2_O (33 mg, 0.32 mmol) was added with stirring at 5 °C–10 °C. After completion of the addition, the reaction mixture was stirred for 3 h at room temperature. After completion of the reaction, the mixture was washed with water and adjusted to pH 6 with 10% aqueous HCl. The organic layer was washed with saturated aqueous NaCl, dried over anhydrous Na_2_SO_4_ and concentrated to give the crude intermediate 1. Then, intermediate 1 was dissolved in DCM (2 mL), and NBS (50 mg, 0.28 mmol) was added at 0 °C under N_2_. After stirring for 3 h at room temperature, the mixture was washed with 3% aqueous Na_2_S_2_O_3_. The organic layer was dried over anhydrous Na_2_SO_4_ and concentrated to give the crude intermediate 2. The intermediate 2 and compound **11a** (155 mg, 0.27 mmol) were dissolved in DCM (2.4 mL), and TMSOTf (60 mg, 0.27 mmol) was added with stirring at −60 °C. The reaction mixture was stirred for 0.5 h. After completion of the reaction, the mixture was warmed to 0 °C, and saturated aqueous NaHCO_3_ (1.2 mL) was added, and followed by stirring for 10 min. The mixture was washed with saturated aqueous NaCl (1.2 mL), and the organic layer was concentrated under reduced pressure to give the crude intermediate 3. The intermediate 3 was dissolved in THF/MeOH (2:3, 4 mL), and NaOH (43 mg, 1.08 mmol) was slowly added. After stirring for 4 h under reflux, the mixture was cooled and concentrated to give a semisolid. The semisolid was dissolved in EtOAc (2 mL) and washed with saturated aqueous NaCl (2 mL), and the organic layer was concentrated to give crude intermediate 4. The intermediate 4 (352 mg in theoretical amount) was dissolved in THF/MeOH (1:2, 3 mL). Aqueous NaHCO_3_ (68 mg, 0.81 mmol) and 20% Pd(OH)_2_/C (70 mg) were added with stirring. The reactor was sealed and purged with N_2_ and then with H_2_. The reactor was pressurized with H_2_ (15 psi), and stirring was started. After stirring for 6 h at room temperature, the reactor was vented and purged with N_2_. AcOH (34 mg, 0.57 mmol) was added and followed by stirring for 10 min. The reaction mixture was filtered through a cartridge (Nylon 6, 0.45 µm), the filtrate was concentrated under reduced pressure, and purified by octadecylsilyl silica gel column chromatography using water/MeOH (3:2) as eluent to give product **13c** (32 mg, 20% yield for 5 steps) as a yellow solid. [α]^25^_D_‐238.0° (*c* 0.10, CH_3_OH). ^1^H‐NMR (400 MHz, D_2_O, *δ* ppm): 6.85–6.79 (m, 2H), 6.76 (d, *J* = 1.7 Hz, 1H), 6.68 (d, *J* = 1.7 Hz, 1H), 6.61–6.51 (m, 2H), 5.94 (s, 1H), 5.59 (s, 1H), 4.55 (d, *J* = 8.0 Hz, 1H), 4.37–4.20 (m, 3H), 3.91 (m, 1H), 3.74 (s, 3H), 2.84 (dd, *J* = 15.9, 5.8 Hz, 1H), 2.45 (dd, *J* = 15.9, 8.9 Hz, 1H). ^13^C‐NMR (100 MHz, D_2_O, *δ* ppm): 156.62, 155.01, 154.38, 153.81, 153.09, 152.76, 147.18, 144.79, 144.48, 143.79, 130.56, 130.52, 121.00, 120.39, 116.13, 115.84, 114.90, 111.17, 109.04, 106.99, 101.48, 95.23, 81.83, 80.73, 72.61, 66.99, 55.83, 36.86, 27.58. HRMS (ESI) Calcd for C_31_H_29_O_12_ [M+H]^+^ 593.1659, found 593.1650.

### Synthesis of (+)‐Catechin‐(4α,8)‐[4′‐O‐Methyl‐(+)‐Catechin] (**13d**)

Compound **12c** (200 mg, 0.27 mmol), DMAP (1.7 mg, 0.01 mmol), and TEA (69 mg, 0.68 mmol) were dissolved in DCM (2 mL), and Ac_2_O (33 mg, 0.32 mmol) was added with stirring at 5 °C–10 °C. After completion of the addition, the reaction mixture was stirred for 3 h at room temperature. After completion of the reaction, the mixture was washed with water and adjusted to pH 6 with 10% aqueous HCl. The organic layer was washed with saturated aqueous NaCl, dried over anhydrous Na_2_SO_4,_ and concentrated to give the crude intermediate 1. Then, intermediate 1 was dissolved in DCM (2 mL), and NBS (50 mg, 0.28 mmol) was added at 0 °C under N_2_. After stirring for 3 h at room temperature, the mixture was washed with 3% aqueous Na_2_S_2_O_3_. The organic layer was dried over anhydrous Na_2_SO_4_ and concentrated to give the crude intermediate 2. The intermediate 2 and compound **11b** (155 mg, 0.27 mmol) were dissolved in DCM (2.4 mL), and TMSOTf (60 mg, 0.27 mmol) was added with stirring at −60 °C. The reaction mixture was stirred for 0.5 h. After completion of the reaction, the mixture was warmed to 0 °C, and saturated aqueous NaHCO_3_ (1.2 mL) was added, followed by stirring for 10 min. The mixture was washed with saturated aqueous NaCl (1.2 mL), and the organic layer was concentrated under reduced pressure to give the crude intermediate 3. The intermediate 3 was dissolved in THF/MeOH (2:3, 4 mL), and NaOH (43 mg, 1.08 mmol) was slowly added. After stirring for 4 h under reflux, the mixture was cooled and concentrated to give a semisolid. The semisolid was dissolved in EtOAc (2 mL) and washed with saturated aqueous NaCl (2 mL), and the organic layer was concentrated to give crude intermediate 4. The intermediate 4 (352 mg in theoretical amount) was dissolved in THF/MeOH (1:2, 3 mL). Aqueous NaHCO_3_ (68 mg, 0.81 mmol) and 20% Pd(OH)_2_/C (70 mg) were added with stirring. The reactor was sealed and purged with N_2_ and then with H_2_. The reactor was pressurized with H_2_ (15 psi), and stirring was started. After stirring for 6 h at room temperature, the reactor was vented and purged with N_2_. AcOH (34 mg, 0.57 mmol) was added and followed by stirring for 10 min. The reaction mixture was filtered through a cartridge (Nylon 6, 0.45 µm), the filtrate was concentrated under reduced pressure, and purified by octadecylsilyl silica gel column chromatography using water/MeOH (3:2) as eluent to give product **13d** (20 mg, 12% yield for 5 steps) as a yellow solid. [α]^25^_D_‐266.0° (*c* 0.10, CH_3_OH). ^1^H‐NMR (400 MHz, D_2_O, *δ* ppm): 6.82 (d, *J* = 8.4 Hz, 1H), 6.77 (d, *J* = 8.2 Hz, 1H), 6.71 (d, *J* = 2.1 Hz, 1H), 6.54 (d, *J* = 2.1 Hz, 1H), 6.53–6.44 (m, 2H), 6.10 (s, 1H), 5.96 (d, *J* = 2.3 Hz, 1H), 5.65 (d, *J* = 2.3 Hz, 1H), 4.49 (d, *J* = 8.0 Hz, 1H), 4.33–4.20 (m, 3H), 3.75–3.67 (m, 4H), 2.73 (dd, *J* = 15.9, 5.6 Hz, 1H), 2.40 (dd, *J* = 15.9, 8.6 Hz, 1H). ^13^C‐NMR (100 MHz, D_2_O, *δ* ppm): 157.24, 155.56, 155.02, 154.37, 153.60, 153.38, 147.89, 145.00, 144.98, 144.33, 131.76, 131.21, 120.97, 120.55, 116.76, 116.40, 114.91, 112.60, 109.59, 107.61, 101.98, 95.79, 82.45, 80.96, 73.22, 67.62, 56.55, 37.45, 27.87. HRMS (ESI) Calcd for C_31_H_29_O_12_ [M+H]^+^ 593.1659, found 593.1646.

### Cell Culture

HT22 cells (Thermo Scientific, USA) were cultured at 37 °C in Dulbecco's modified Eagle medium (DMEM)/F‐12 (Gibco, USA) supplied with 10% fetal bovine serum (FBS, Sigma‐Aldrich, USA) and 1% penicillin/streptomycin (BasalMedia, China) in a humidified atmosphere in the presence of 5% CO_2_. When 80%–90% confluence was reached, cells were subcultured after treatment with a trypsin‐EDTA mixture (Gibco, USA).

### OGD/R Model In Vitro

HT22 cells were washed with phosphate buffered saline (PBS), then DMEM sugar‐free medium (Gibco, USA) was added and placed in the anaerobic incubator of 5% CO_2_, 1% O_2_, and 37 °C for 6 h. PB3 or other compounds were previously dissolved in DMEM/F‐12 with 10% FBS and 1% penicillin/streptomycin. After undergoing OGD for 6 h, the medium was replaced by DMEM/F‐12 with 10% FBS and 1% penicillin/streptomycin containing PB3 or other compounds and kept in a humidified 5% CO_2_, 37 °C incubator for 24 h.

### Animals

Male C57BL/6 mice (6–8 weeks) were procured from Shanghai Lingchang Biotechnology Co., Ltd. (China). The mice were domiciled within a controlled environment maintained under specific pathogen‐free conditions. All animal procedures were performed in accordance with the Animal Welfare Act Guide for Use and Care of Laboratory Animals and were approved by the Institutional Animal Care and Use Committee (IACUC), School of Pharmacy, Fudan University (2022‐02‐BXZ‐0l).

### tMCAO Model In Vivo

Mice were kept anesthetized during surgery with 1% pentobarbital sodium (50 mg kg^−1^, Sigma‐Aldrich, USA) by *i.p*. injection. Transient ischemia was induced by using the suture occlusion technique according to our previous studies.^[^ ^10^ ^]^ Expose the common carotid artery area, insert a 6–0 sized silicon rubber‐coated filament into the left internal carotid artery until it reaches the approximate branch of the left middle cerebral artery, and leave it for 1–1.5 h to block blood flow. Then, the suture and the filament were removed, and the wounds were sutured. 24 h after the operation, a series of phenotypic evaluations was conducted on each mouse. The Bederson score was applied according to our previous studies.^[^ ^10^ ^]^

### Cell Viability Detection

The CCK‐8 assay kit (Yeasen, China) was used to assess cell viability. All operations were performed following instructions. HT22 cells were seeded into the 96‐well plate (3000 cells per well). The cells were cultured at 37 °C with 5% CO_2_ in a humidified incubator overnight. After 6 h of OGD, the medium was replaced by DMEM/F‐12 with 10% FBS and 1% penicillin/streptomycin containing PB3 or other compounds and kept in a humidified 5% CO_2_, 37 °C incubator for 24 h. Then, the cells were washed with PBS, the medium was replaced with DMEM/F‐12 with 10% FBS and 1% penicillin/streptomycin containing 10% CCK‐8 solution, and incubated for 1 h at 37 °C. The absorbance was assessed by a Synergy H1 multi‐mode microplate reader (BioTek, USA) at 450 nm.

### Apoptosis Assay

Apoptosis level was evaluated by TUNEL staining and flow cytometry. For TUNEL staining, HT22 cells were treated with 4% paraformaldehyde for 15 min. Then the fixed brain tissue slices and HT22 cells were permeabilized for 5 min using 1% Triton X‐100 (Sigma‐Aldrich, USA) dissolved in PBS. TUNEL staining was executed by following the instructions, using a one‐step TUNEL apoptosis assay kit (Meilunbio, China). Red fluorescence was utilized to identify apoptotic cells, while 4′, 6‐diamidino‐2‐phenylindole (DAPI) was used to counterstain the nuclei. The fluorescence microscope (Carl Zeiss, Germany) was used to obtain images. For flow cytometry, apoptotic cells were determined with the Annexin V‐Alexa Fluor 488/PI apoptosis detection kit (Yeasen, China) by following the instructions. EDTA‐free trypsin (Gibco, USA) was added to HT22 cells (2.5 × 10^5^ cells). The cells were collected and washed with pre‐cooling PBS 2 times, then suspended in 250 µL 1× binding buffer. 100 µL cell suspension was dyed with 5 µL Annexin V‐Alexa Fluor 488 and 10 µL PI and plunged into darkness at room temperature for 15 min. Then the cell suspension was mixed with 400 µL of 1× binding buffer. Cell apoptosis was immediately determined by Cytek Aurora (Cytek Biosciences, USA).

### Immunoblotting Analysis

Cells and tissues were homogenized in RIPA buffer (Beyotime, China) containing protease inhibitors (Biovision, USA) on ice for 30 min. After centrifugation (13000 r min^−1^, 4 °C) for 10 min, the resulting supernatant was collected. The protein concentrations were determined by a BCA protein assay kit (Thermo Scientific, USA). After separating a 10% sodium dodecyl sulfate (SDS)‐polyacrylamide gel, protein on the gel was then transferred onto nitrocellulose membranes (Merck, USA), which were blocked with 5% bovine serum albumin for 1 h to prevent non‐specific binding. Then, the anti‐caspase 3 antibody (catalog no. 9662, Cell Signaling Technology, USA), anti‐Bcl‐2 antibody (catalog no. GTX100064, GeneTex, USA), anti‐G3BP1 antibody (catalog no. 66486‐1‐Ig, Proteintech, China), anti‐β‐tubulin antibody (catalog no. 66240‐1‐Ig, Proteintech, China), and anti‐β‐actin antibody (catalog no. 66009‐1‐Ig, Proteintech, China) were used for treating the membrane overnight at 4 °C. Bound antibodies were detected with the species‐appropriate horseradish peroxidase‐labeled secondary antibody (Jackson ImmunoResearch, USA) using ECL reagents (ShareBio, China). Protein‐specific signals were detected using a Bio‐Rad Imager (Bio‐Rad, USA), and the bands were quantified by densitometric analysis software (ImageJ). β‐tubulin or β‐actin was used as a loading control.

### Assessment of Cerebral Infarction Volume

Cerebral infarction volume was evaluated by Nissl staining or 2,3,5‐triphenyltetrazolium chloride staining. The infarct volume was computed using image‐processing software (Image J), and the percentage of the infarct volume was calculated according to our previous studies.^[^ ^10^ ^]^ For Nissl staining, the fixed brain tissue slices were washed twice with ultrapure water for 2 min each time. Next, they were stained with Nissl staining solution (Beyotime, China) at 37 °C for 10 min, washed twice with ultrapure water, washed once with 95% ethanol, and washed twice with 70% ethanol. Then, they were dehydrated twice with 95% ethanol for 2 min each time. The slices were placed in xylene for another 5 min and then coverslipped. For 2,3,5‐triphenyltetrazolium chloride staining, brains were frozen for 30 min at −20 °C and coronally cut into 5 slices. A 1% 2,3,5‐triphenyltetrazolium chloride (Sigma‐Aldrich, USA) solution was used to stain the brain slices for 30 min at 37 °C. The pale region of the hemisphere represented the infarcted part.

### Immunofluorescence Analysis

Immunofluorescence analysis of the stress granule (SG) core component G3BP1 was utilized to investigate the SG formation in HT22 cells and the cortex. HT22 cells were treated with 4% paraformaldehyde for 15 min. Then the fixed brain tissue slices and HT22 cells were permeabilized for 5 min using 1% Triton X‐100 (Sigma‐Aldrich, USA) dissolved in PBS and blocked with a PBS solution containing 10% goat serum for 1 h, followed by incubation with anti‐G3BP1 antibody (catalog no. 66486‐1‐Ig, Proteintech, China) at 4 °C overnight. After being washed with PBS 3 times for 5 min each, samples were incubated with secondary antibody (Alexa Fluor 568 Goat anti‐mouse IgG, Invitrogen, USA) in the dark for 2 h at room temperature. The nucleus was stained with DAPI. Fluorescent pictures were obtained with a fluorescence microscope (Carl Zeiss, Germany).

### Preparation and Identification of the PB3 Probe

NHS‐activated agarose beads (10 mL, Cytiva, USA) were pre‐activated with 1 mm HCl and washed five times with PBS. 20 mL of 1 m 2,2′‐(ethylenedioxy)bis(ethylamine) solution was added and incubated for 4 h at room temperature. Then, the beads were washed five times with PBS, five times with 10 mm HCl, five times with ultrapure water, three times with ethanol, and once time with ACN, obtaining the linker‐beads. 1‐(3‐Dimethylaminopropyl)‐3‐ethylcarbodiimide hydrochloride (259 mg, 1.36 mmol), *N*‐hydroxysuccinimide (156 mg, 1.36 mmol), and 4‐[3‐(trifluoromethyl)‐3*H*‐diazirin‐3‐yl] benzoic acid (68 mg, 340 µmol) were dissolved in 3 mL ACN and incubated at room temperature for 2 h in the dark. Then, the reaction mixture and 300 µL of 1 m triethylammonium bicarbonate were added to the linker‐beads and incubated overnight at room temperature, protected from light. The beads were washed two times with ACN, three times with ethanol, four times with ultrapure water, and four times with ethanol, obtaining the photoaffinity probe. Two‐thirds of the photoaffinity probe was mixed with an ethanol solution of PB3 and concentrated under reduced pressure at room temperature in the dark (0.05 mmol of PB3 for every 1 mL of beads). The other one‐third of the photoaffinity probe was also concentrated under reduced pressure in the dark at room temperature. The dried beads were irradiated at 365 nm (4 J cm^−2^) for 30 min by a VOSHIN07‐II ultraviolet crosslinker (Voshin, China). The beads were washed six times with ethanol, four times with ultrapure water, and four times with ethanol, obtaining the PB3 probe and control probe. The beads were further analyzed by a Nicolet iS5 Fourier transform infrared spectrometer (Thermo Scientific, USA) and a Sigma 300 field‐emission scanning electron microscope (Carl Zeiss, Germany). The preparation of the PB3 probe was also confirmed by quantification of PB3 in eluent using a Waters e2695 system (Waters, USA).

### Enrichment of the Target Proteins of PB3

HT22 cells were lysed in NETN (3% 5 m NaCl + 0.1% 0.5 m EDTA (pH 8.0) + 2% 1 m Tris HCl (pH 8.0) + 0.5% NP‐40 0.5 mL + 94.4% ultrapure water, containing protease inhibitors). The extracted proteins (1.8 mg) were preincubated in the presence or absence of PB3 (100 µm) at 4 °C overnight, and then the PB3 probe or control probe (20 µL each) was added to each sample and incubated at 4 °C for 6 h. The beads were washed with NETN (1 mL) three times, eluted with a mixture of 18 µL 2% SDS and 6 µL 4× loading buffer (Thermo Scientific, USA), and then boiled for 5 min at 98 °C. The eluted proteins were analyzed according to the digestion in gel method and further analyzed by LC‐MS/MS detection system with label‐free quantification.

### LC‐MS/MS Detection

The LC‐MS/MS detection system consisted of an EASY nLC 1200 high‐performance liquid chromatography (HPLC) instrument (Thermo Scientific, USA) coupled to an Orbitrap Exploris 480 mass spectrometer (Thermo Scientific, USA) with a nanoelectrospray ion source (Thermo Scientific, USA). Digested peptide samples re‐dissolved in buffer A (0.1% formic acid, FA) were loaded into a 2 cm self‐packed trap column (100‐µm inner diameter, ReproSil‐Pur C18‐AQ, pore size 120 Å, particle size 3 µm, Dr. Maisch, Germany) and then separated with a gradient of 6%‐100% mobile phase B (80% ACN and 0.1% FA) at a flow rate of 600 nL min^−1^ for 150 min by a 30 cm self‐packed silica microcolumn (150 µm inner diameter, ReproSil‐Pur C18‐AQ, pore size 120 Å, particle size 1.9 µm, Dr. Maisch, Germany). The LC‐MS/MS‐based proteomic experiment was conducted with Field Asymmetric Ion Mobility Spectrometry (FAIMS). FAIMS voltages were set to −46 and −65 V. The Orbitrap Exploris 480 was set to the data‐dependent acquisition mode, and other parameters were consistent and set as follows: protein quantification consisting of an MS1 scan at a resolution of 12 0000, max injection time of 80 ms, and scan range of 300–1400 *m/z*, MS2 scans with higher‐energy collision dissociation detected in the ion trap first (resolution = 7500, isolation window = 1.6 *m/z*, max injection time = 22 ms, normalized collision energy = 30%).

### Protein Identification by MaxQuant‐Based Database Searching

The tandem mass spectra were searched against the mouse UniProt database (version 20240404, 17207 sequences) using MaxQuant (version 2.4.2.0).^[^ ^59^ ^]^ Trypsin was selected as the proteolytic enzyme, and two missed cleavage sites were allowed. Carbamidomethyl (C) was set as the fixed modification. The oxidation of methionine (M) and acetylation of the protein N‐terminal were set as the variable modifications. The first search mass tolerance was 20 ppm, and the main search peptide tolerance was 4.5 ppm. The label‐free quantification min ratio count was 2. The false discovery rates of the peptide‐spectrum matches and proteins were set to less than 0.01.

### SPR Analysis

The SPR analyses were carried out using a Biacore T200 instrument (GE Healthcare, USA) with the Series Sensor Chip CM5 (GE Healthcare, USA). The recombinant human G3BP1 protein was purchased from Hubei Ipodix Biotechnology Co., Ltd. (China). The protein was immobilized on the chip using an amine coupling kit (GE Healthcare, USA). The blank channel served as the negative control. The binding experiments were performed at a flow rate of 30 µL min^−1^. For the measurement of the kinetic parameters of PB3 and **6c**, gradient dilutions of PB3 and **6c** were injected into the immobilized protein. The running buffer was PBS. All biosensor data were processed and analyzed with Biacore T200 Evaluation software version 3.2 (GE Healthcare, USA) and fitted by a 1:1 binding model.

### Cellular Thermal Shift Assay

HT22 cells were grown to 90% confluency and harvested by trypsin and resuspended in 1 mL PBS. The samples were subjected to three freeze–thaw cycles in liquid nitrogen. Each cell lysate was centrifuged (12000 g for 30 min at 4 °C), and the supernatant was collected. The supernatants were treated with PB3 (or PBS control) for 20 min. Subsequently, they were aliquoted into Eppendorf tubes and heated for 3 min to 44, 48, 52, 56, or 60 °C. The soluble fractions were analyzed by SDS‐polyacrylamide gel electrophoresis and immunoblotted with anti‐G3BP1 antibody.

### Pull‐Down Analysis

HT22 cells were grown to 90% confluency and harvested by trypsin and resuspended in 1 mL PBS (containing protease inhibitors and 0.1% Triton X‐100). The samples were subjected to three freeze–thaw cycles in liquid nitrogen. Each cell lysate was centrifuged (12000 g for 30 min at 4 °C), and the supernatant was collected. The protein concentrations were determined by a BCA protein assay kit (Thermo Scientific, USA). The lysates were adjusted to 2 mg mL^−1^ and treated with 200 µm PB3 or DMSO, labeled with 200 µm of PB3‐BP or DMSO, and then put on ice under UV radiation at 365 nm for 1 h. The labeled proteins were precipitated with chloroform‐methanol. Then the precipitations were resuspended with 1.2% SDS/PBS and combined with 300 µm biotin‐PEG3‐azide, 100 µm tris(benzyltriazolylmethyl)amine, 1 mm tris(2‐carboxyethyl)phosphine hydrochloride, and 1 mm CuSO_4_ for 1 h. After this reaction, the proteomes were extracted again with chloroform‐methanol. The proteins were solubilized with 1.2% SDS/PBS and diluted 5x with PBS. The solubilized proteins were incubated with streptavidin beads (100 µL of slurry, Thermo Scientific, USA) at room temperature for 3 h with rotation. The beads were then washed with 1 mL of PBS three times and 1 mL of water one time before being eluted with a mixture of 24 µL of 2% SDS and 8 µL of 4× loading buffer (Thermo Scientific, USA) and then boiled for 5 min at 98 °C. The eluted proteins were analyzed by SDS‐polyacrylamide gel electrophoresis and immunoblotted with anti‐G3BP1 antibody.

### Molecular Docking

The Crystal structure of the NTF2L domain of G3BP1 (PDB code: 8V1L) ^[^ ^33^ ^]^ was retrieved from the Protein Data Bank (www.rcsb.org). The AutoDock 4.2 software with the Lamarckian genetic algorithm was used for the molecular docking to predict the binding modes. The ADT and grid calculations were carried out using grid box dimensions of 60 × 60 × 60 Å with 0.375 Å spacing, followed by the space creations around R107. Pretreatment of the compounds and the receptor structure for docking was carried out with the AutoDockTools program suite (mgltools.scripps.edu). Default parameters were used, and one hundred independent docking runs were conducted for each compound. The interactions between biological macromolecules and ligands were analyzed using the PLIP 2.3.0 program.^[^ ^60^ ^]^

### Molecular Dynamics Simulation

Gromacs2022.3 software was used for molecular dynamics simulation.^[^ ^61^, ^62^ ^]^ For small molecule preprocessing, AmberTools22 is used to add the GAFF force field to small molecules, while Gaussian 16 W is used to hydrogenate small molecules and calculate RESP potential. Potential data will be added to the topology file of the molecular dynamics system. The simulation conditions were carried out at a static temperature of 310 K and atmospheric pressure (1 Bar). Amber99sb‐ildn was used as a force field, water molecules were used as a solvent (Tip3p water model), and the total charge of the simulation system was neutralized by adding an appropriate number of Na^+^ ions. The simulation system adopts the steepest descent method to minimize the energy and then carries out the isothermal isovolumic ensemble (NVT) equilibrium and isothermal isobaric ensemble (NPT) equilibrium, respectively, with a duration of 100 ps. Finally, the free molecular dynamics simulation was performed, and the total duration was 100 ns. After the simulation was completed, the built‐in tool of the software was used to analyze the MMGBSA free energy.

### siRNA Transfection In Vitro

Mouse G3BP1 and control siRNA were obtained from Shanghai GenePharma Co., Ltd. (China). HT22 cells at 50%–60% confluence were added to the prepared Lipofectamine RNA iMAX (Thermo Scientific, USA), Opti‐MEM medium (Gibco, USA), and siRNA complexes. After incubating for 24 h, the medium was removed. siRNA sequences used in this study are listed in Table S3 (Supporting Information).

### RT‐qPCR

Total RNA from HT22 cells was extracted with Trizol. The concentration and purity of RNA were determined by Nanodrop (Thermo Scientific, USA), and RNA was reverse transcribed by PrimeScript Reverse Transcription Master Mix (TaKaRa, Japan). Then RT‐qPCR was performed using Hieff qPCR SYBR Green Master Mix (Yeasen, China). The expression level of the transcripts was normalized to the constitutive expression level of β‐actin. The primers used for amplification are listed in Table S4 (Supporting Information).

### RIP Assay

RIP assay was performed with the BeyoRIP RIP Assay Kit (Beyotime, China) using the manufacturer's protocol. HT22 cells were washed with PBS and centrifuged, then the cell was resuspended in complete lysis buffer. 3–5 µg antibody was pre‐bound to protein A/G agarose beads in NT2 wash buffer for 0.5 h and then incubated with 270 µL cell lysates for 4 h at 4 °C with rotation, and RNA was eluted from the beads by incubating with 100 µL elution buffer for 0.5 h at 55 °C. The eluted RNA was dissolved with RNase‐free water. Enrichment of certain fragments was determined by RT‐qPCR.

### In Vitro Assessment of BBB Permeability

The Transwell filters (0.4 µm pore) were used to evaluate the crossing capacity of the BBB of selected compounds. The basolateral side of the wells was filled by DMEM (Gibco, USA) with 10% FBS. Then, bEnd.3 cells were seeded on the apical side of the semi‐permeable filter in a humidified atmosphere. The culture medium was changed on alternate days. To verify if the cell monolayer formed junctions, the resistance was daily monitored by determining the trans‐endothelial electrical resistance. The resistance value of an empty filter was subtracted from each measurement. After reaching the confluence, bEnd.3 monolayer cells were washed three times with PBS, and the basolateral compartment was filled with Hanks' Balanced Salt Solution (HBSS). The permeability experiments across the cell monolayer were performed in the apical‐to‐basolateral direction. Transport experiments were assessed by placing the compounds, prepared in HBSS, on the upper side of the monolayers (100 µm/300 µL). At different time points (30, 60, and 120 min), 500 µL samples were taken from the basolateral side, and to replace the withdrawn volume, the same volume of preheated HBSS was added. The amount of cellular drug uptake was measured using a Waters e2695 system (Waters, USA).

### Assessment of Long‐Term Neurological Recovery After tMCAO

The modified neurological severity score was applied according to the previous report.^[^ ^63^ ^]^ The range of the neurological function score extends from 0 to 18 points. A higher score represents more severe neurological deficits. In the rotarod test, according to the previous report,^[^ ^64^ ^]^ mice were tested under the accelerating rotor mode with acceleration from 5 to 20 rpm. The final score for statistical analysis was expressed as the mean time that mice could remain on the rod.

### Pharmacokinetic Study of PB3 and **6c**

Pharmacokinetic analyses were performed using a LC‐40D XS HPLC system (Shimadzu, Japan) coupled with a Sciex Triple Quad 5500 mass spectrometer (AB SCIEX, USA). Tested compounds PB3 and **6c** were administered by *i.p*. injection (48 mg kg^−1^, formulation solvent: normal saline). Blood samples were drawn from the retro‐orbital vein at 0.083, 0.25, 0.5, 1, 2, 4, 8, and 24 h post‐injection with EDTA dipotassium salt as an anticoagulant. Subsequently, the plasma fractions were collected in Eppendorf tubes and centrifuged at 4000 g for 5 min at 4 °C and stored at −80 °C for analysis. Brain tissue was mixed with four‐fold normal saline for homogenization (10 s per time with a 10 s interval for 3–5 times). Then, the mixture was centrifuged at 4000 g for 5 min at 4 °C, and the supernatant was stored at −80 °C for analysis. For sample preparation, the desired serial concentrations of working solutions were achieved by diluting a stock solution of analyte with 50% methanol in water solution. 2 µL of working solutions were added to 10 µL of the blank C57BL/6 mouse plasma/brain homogenate to achieve calibration standards of 10–10 000 ng mL^−1^ (10, 20, 100, 200, 1000, 2000, 4000, 10 000 ng mL^−1^) in a total volume of 12 µL. Three quality control samples at 20 ng mL^−1^, 1600 ng mL^−1^, and 8000 ng mL^−1^ for plasma were prepared independently of those used for the calibration curves. These QC samples were prepared on the day of analysis in the same way as calibration standards.

### Statistical Analysis

For normal data, comparisons between two groups were performed using a Student's *t*‐test, while comparisons between multiple groups were performed using ANOVA when assumptions were met. In the case where data were not normal or groups had unequal variance, comparisons between two groups were performed using a Mann‐Whitney test, while comparisons between multiple groups were performed using a Kruskal‐Wallis test. All analyses were made using the GraphPad Prism statistical software, and *p* < 0.05 was considered to indicate statistical significance. All experiments were performed at least three independent times.

## Conflict of Interest

The authors declare no conflict of interest.

## Supporting information



Supporting Information

## Data Availability

The data that support the findings of this study are available from the corresponding author upon reasonable request.
