# Supplementary material for: Procyanidin B3 and Its Derivatives Alleviate Neuronal Injury by Targeting G3BP1 for Ischemic Stroke Therapy
Source: Adv Sci (Weinh). 2025 Sep 26;12(45):e09781. doi: 10.1002/advs.202509781 (PMC12677652; doi:10.1002/advs.202509781)
Supplement: Supplementary file 1 — Supporting Information [file ADVS-12-e09781-s001.pdf]

Supporting Information

Procyanidin B3 and Its Derivatives Alleviate Neuronal Injury by Targeting G3BP1 for Ischemic Stroke Therapy

Heyanhao Zhang,[#,[a]] Yuyu Zhang,[#,[a]] Yibo Chen,[#,[a]] Wen Zhong,[#,[a]] Subei Tan,[a] Jinghuan Wang,[a] Huanren Yan,[a] Ning Yan,[a] Mengjia Lin,[a] Xinhua Liu,[*,[a]] and Jun Chang[*,[a]]

[a] H. Zhang, Y. Zhang, Y. Chen, W. Zhong, S. Tan, J. Wang, H. Yan, N. Yan, M. Lin, X. Liu, and J. Chang
Phenome Research Center of TCM, Department of Traditional Chinese Medicine, Shanghai Pudong Hospital; Pharmacophenomics Laboratory, Human Phenome Institute, Fudan University, Shanghai, China
E-mail: liuxinhua@fudan.edu.cn; jchang@fudan.edu.cn

[#] These authors contributed equally to this work.

Table of Contents

Copies of NMR Spectra……S3

Copies of HPLC Spectra……S31

Figure S1. PB3 alleviates the neuronal damage and reduces the apoptosis level in the ischemic cortex of tMCAO mice……S33

Figure S2. Preparation and identification of the PB3 probe……S34

Figure S3. Immunoblots showing the expression of G3BP1 under different treatment……S35

Figure S4. Key COSY, HMBC, and ROESY correlations for acetylated PB3-BP……S36

Figure S5. RT-qPCR analysis of the expression of key factors regulating SG dynamics during the degradation of SG……S37

Figure S6. RT-qPCR analysis of G3BP1-bound mRNAs……S38

Figure S7. PB3 enhanced SG level in the ischemic cortex of tMCAO mice……S39

Figure S8. Transport efficiency of 1 and 6c at 100 μM in bEnd.3 cells……S40

Figure S9. The anti-apoptotic effect of 6c……S41

Figure S10. The interaction of 6c and G3BP1……S42

Figure S11. 6c enhanced SG level in HT22 cells under OGD/R injury……S43

Figure S12. Effect of PB3, 6b, and 6c on infarct area and neurological function after 1 h of MCAO and 24 h of reperfusion……S44

Figure S13. Effect of 6c on the long-term neurological recovery after tMCAO……S45

Figure S14. 6c enhanced SG level in the ischemic cortex of tMCAO mice……S46

Table S1. The list of PB3-specific binding proteins……S47

Table S2. MMGBSA analysis of PB3 binding to wild type and mutants of the G3BP1 NTF2L domain……S49

Table S3. siRNA used for transfection……S50

Table S4. Primers used for RT-qPCR analysis……S51

Scheme S1. Synthesis of PB3-BP……S52

Scheme S2. Fragmentation pathway of PB3-BP modified peptides……S53

Copies of NMR Spectra

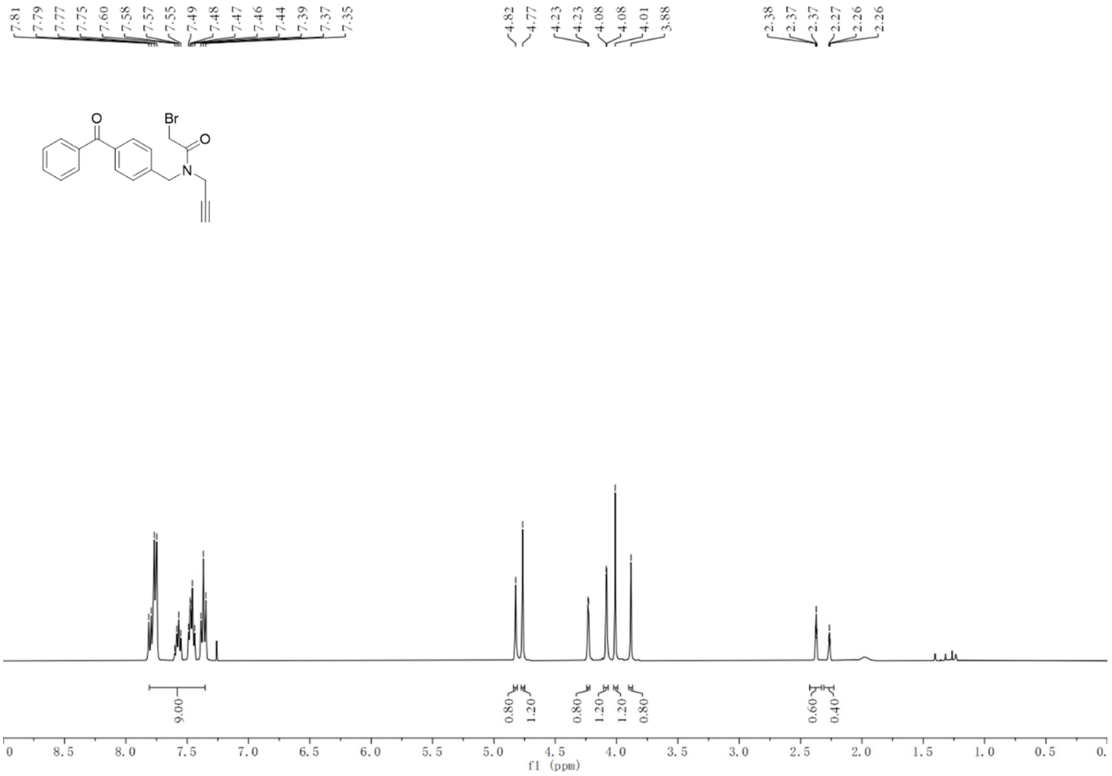

^{1}H-NMR spectrum of compound **S3** (400 MHz, $CDCl_3$)

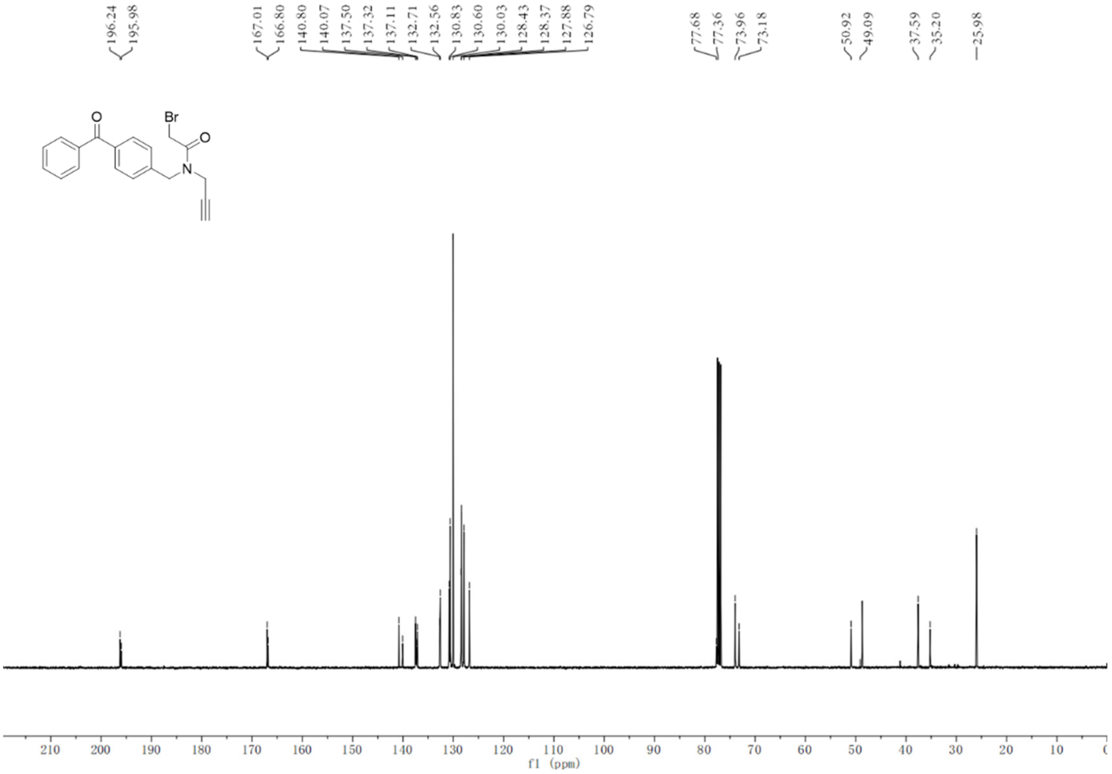

^{13}C-NMR spectrum of compound **S3** (100 MHz, $CDCl_3$)

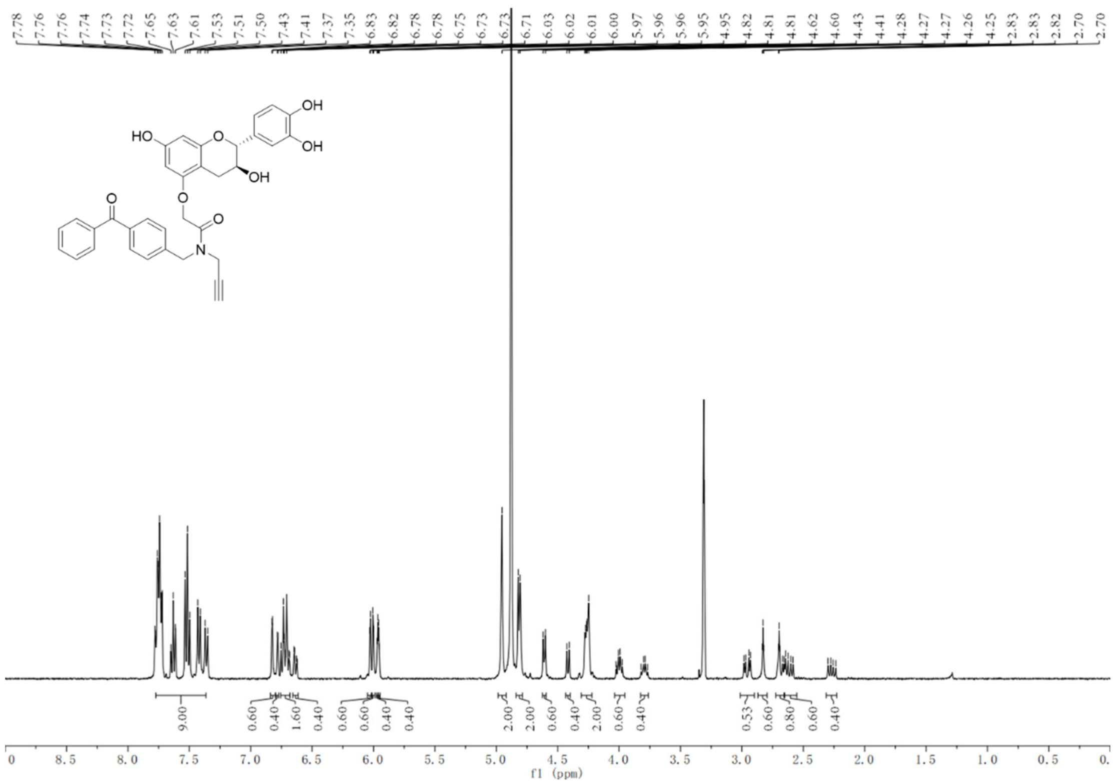

^{1}H-NMR spectrum of compound **S6** (400 MHz, CD_3OD)

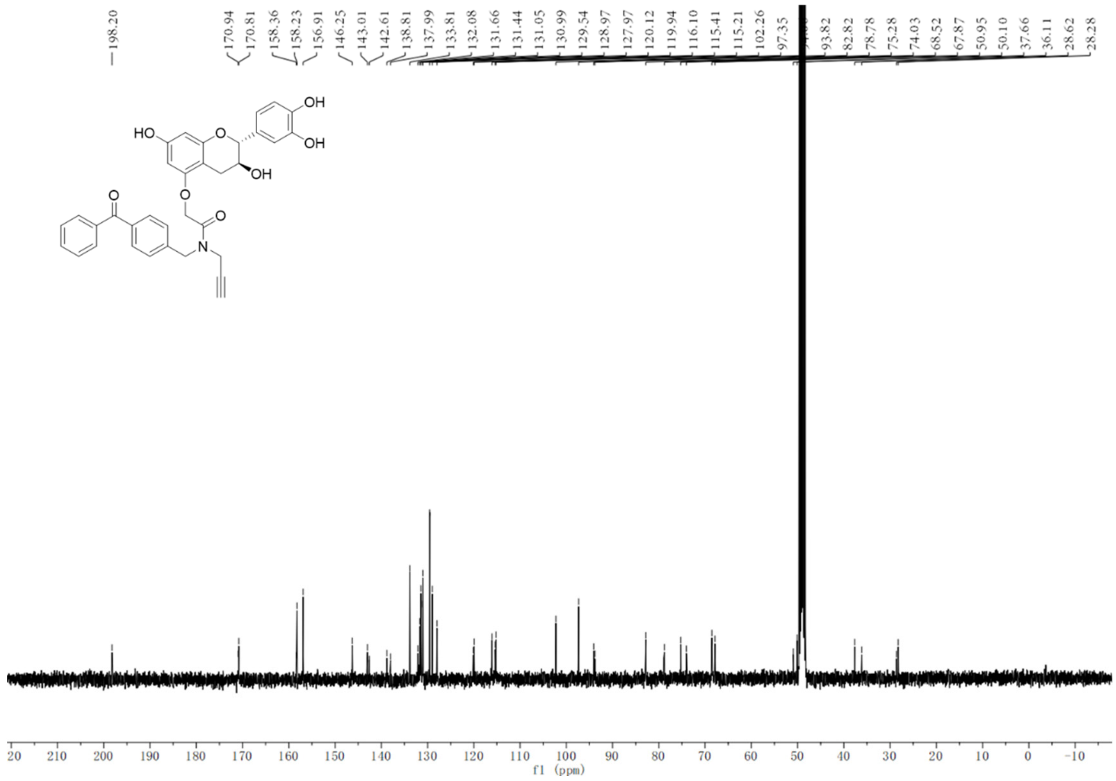

^{13}C-NMR spectrum of compound **S6** (100 MHz, CD_3OD)

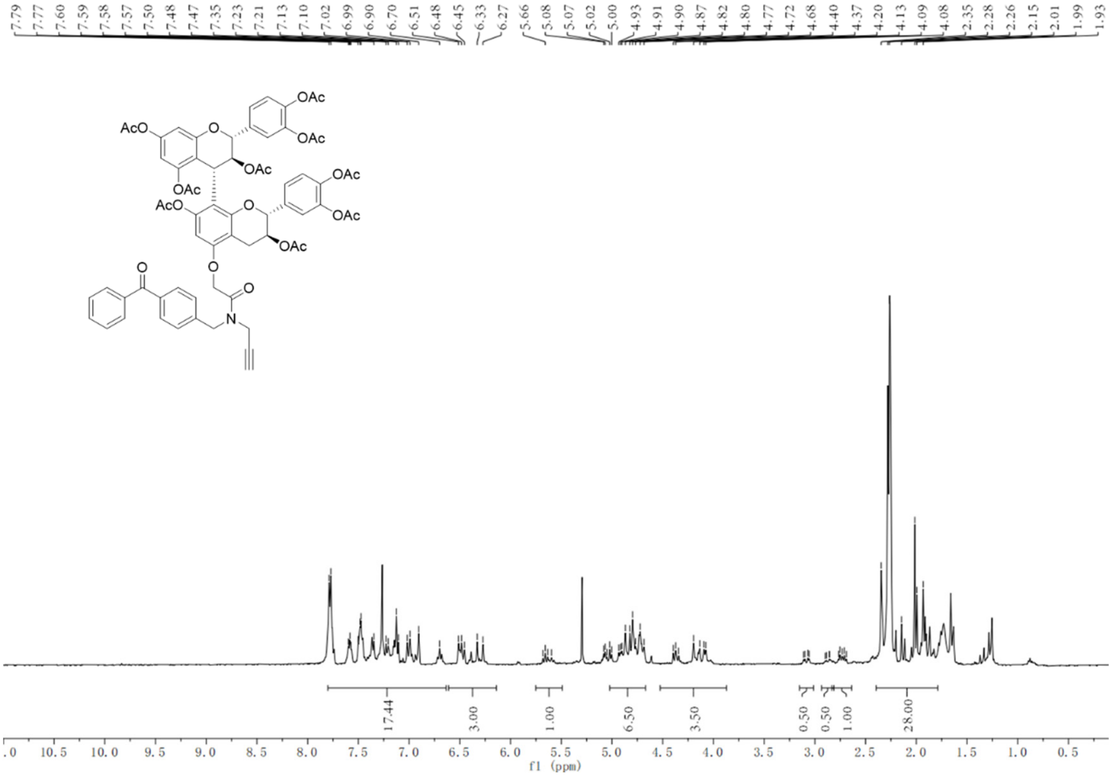

^{1}H-NMR spectrum of compound **S9** (400 MHz, $CDCl_3$)

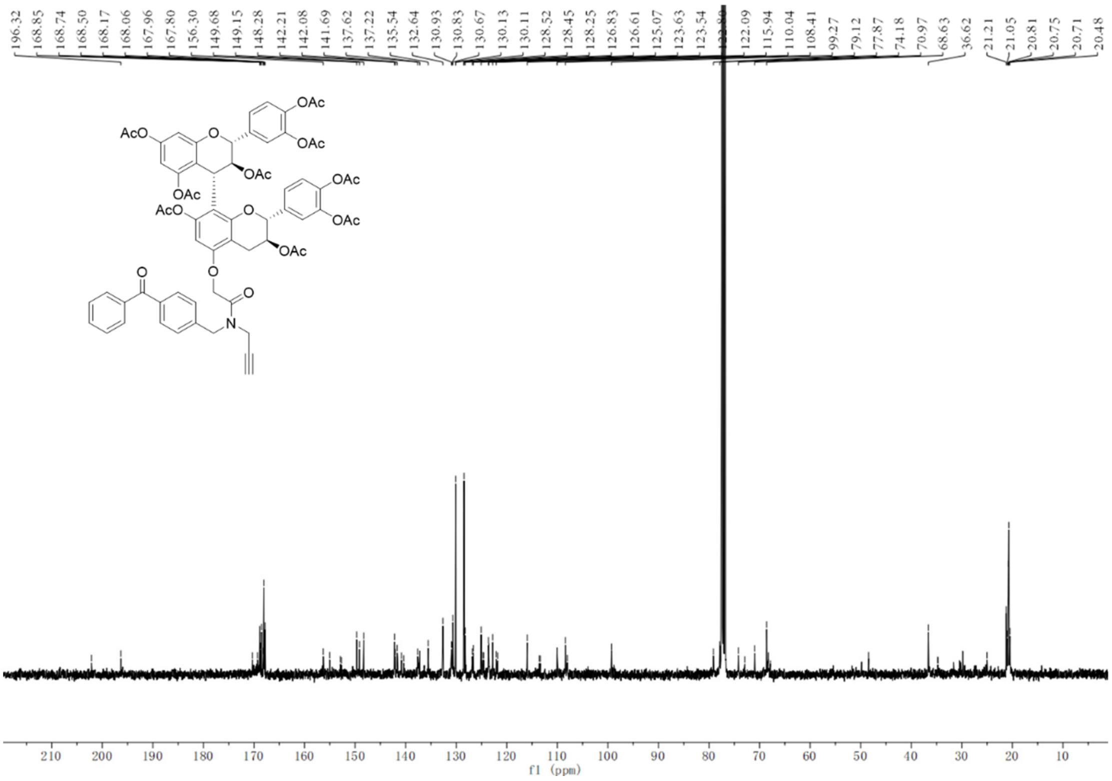

^{13}C-NMR spectrum of compound **S9** (100 MHz, $CDCl_3$)

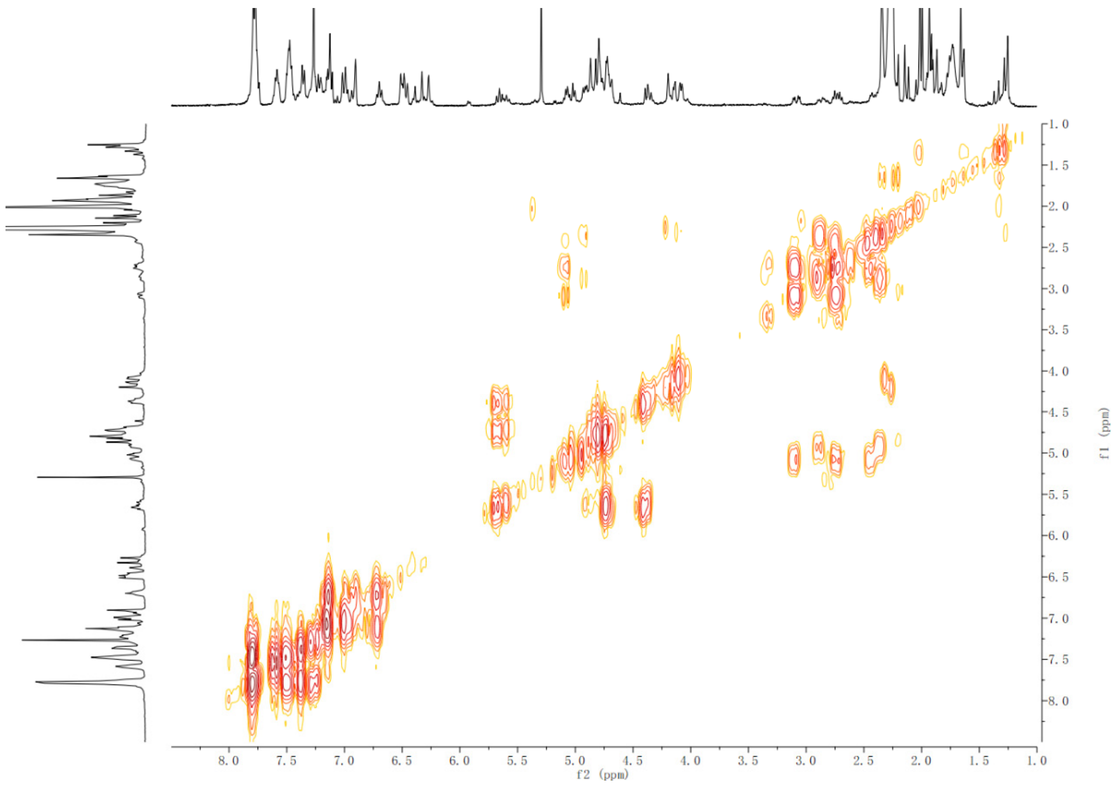

^{1}H-^{1}H COSY spectrum of compound **S9** ($CDCl_3$)

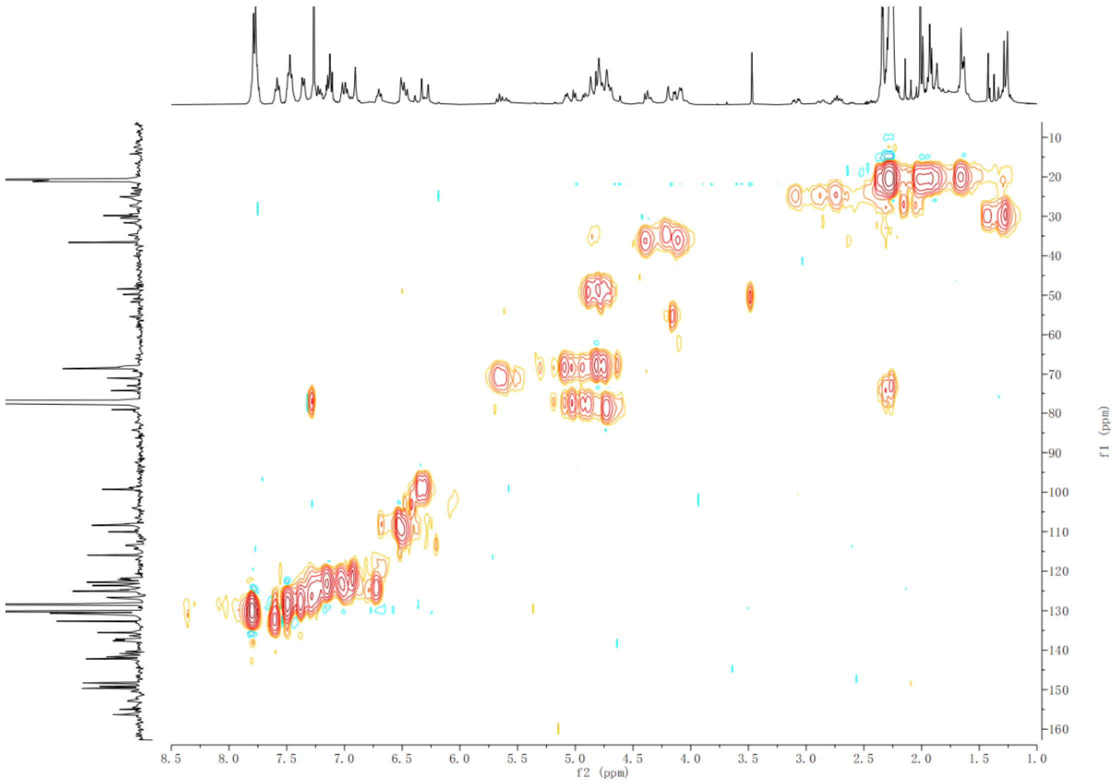

HSQC spectrum of compound **S9** ($CDCl_3$)

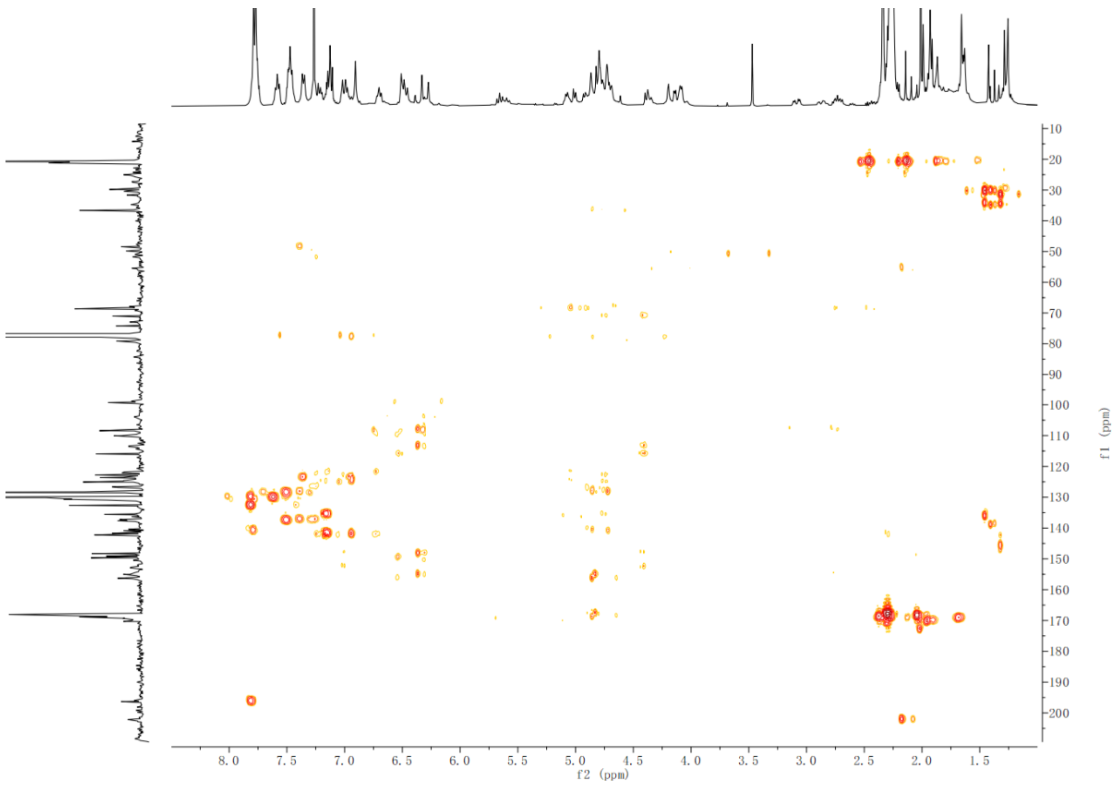

HMBC spectrum of compound **S9** ($CDCl_3$)

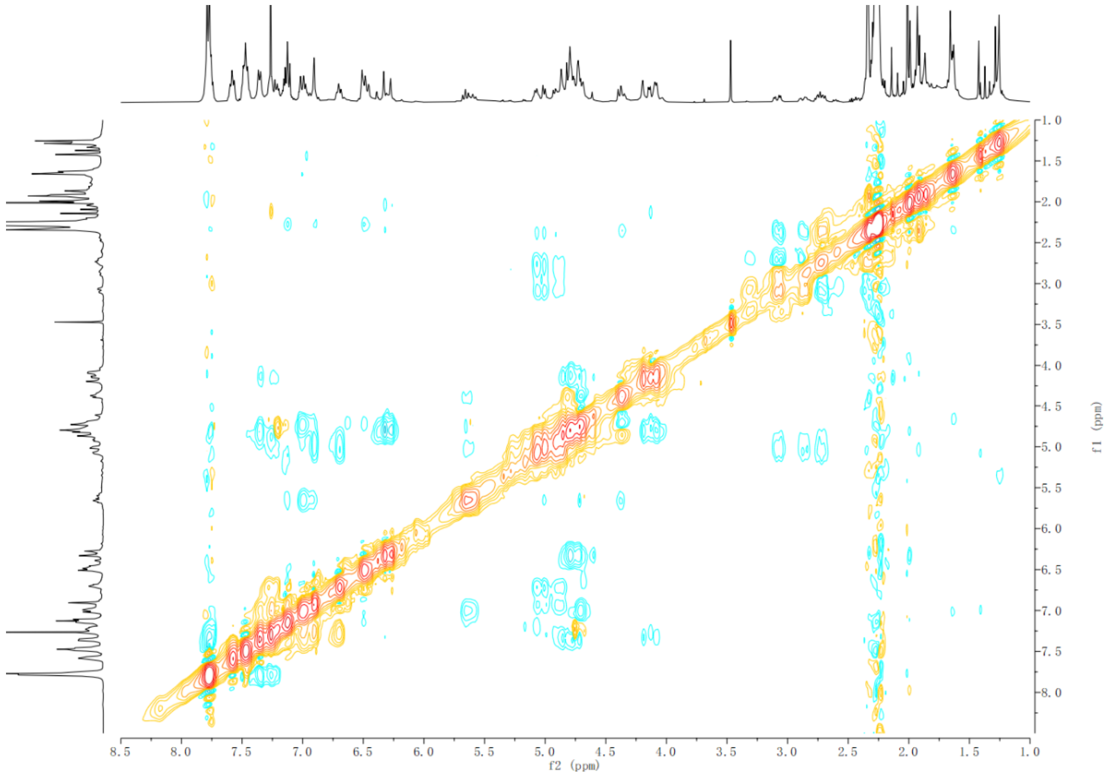

ROESY spectrum of compound **S9** ($CDCl_3$)

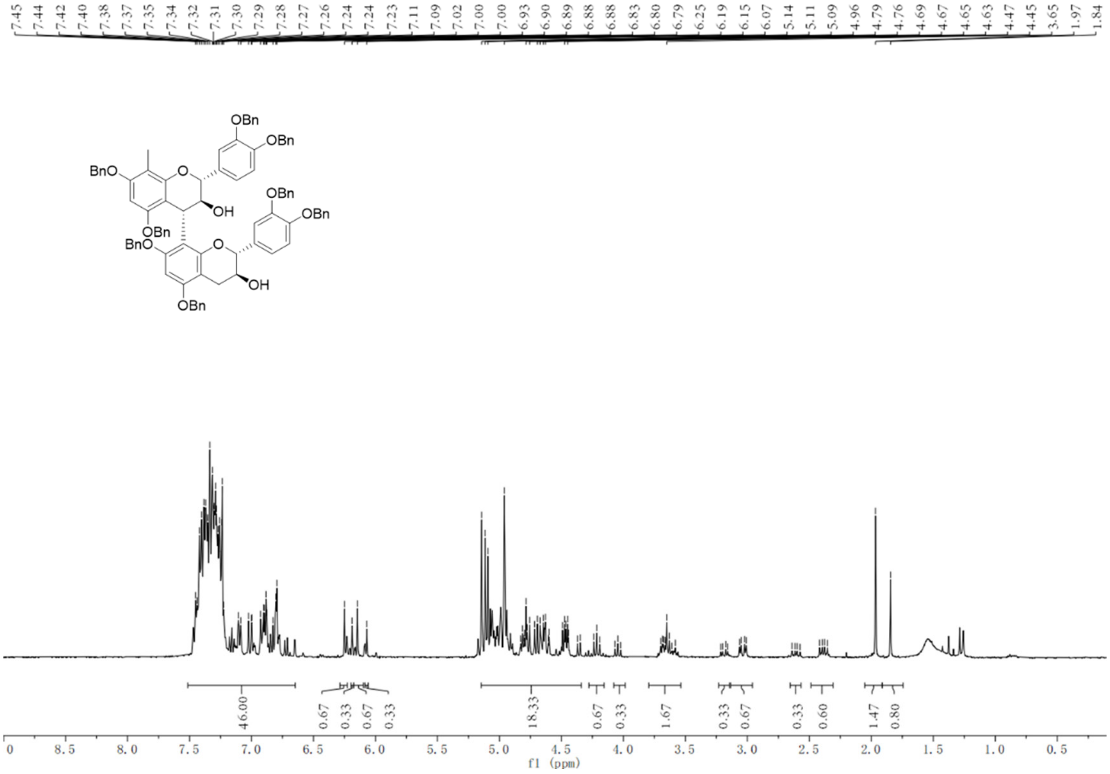

^{1}H-NMR spectrum of compound **5a** (400 MHz, $CDCl_3$)

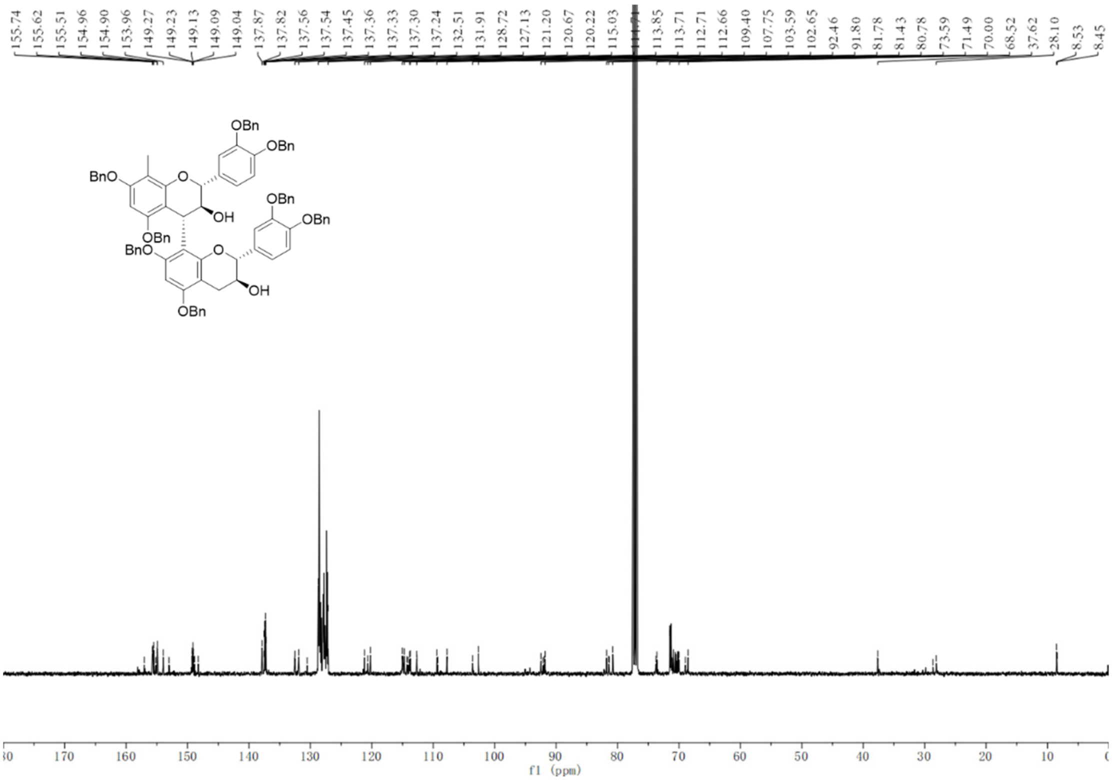

^{13}C-NMR spectrum of compound **5a** (100 MHz, $CDCl_3$)

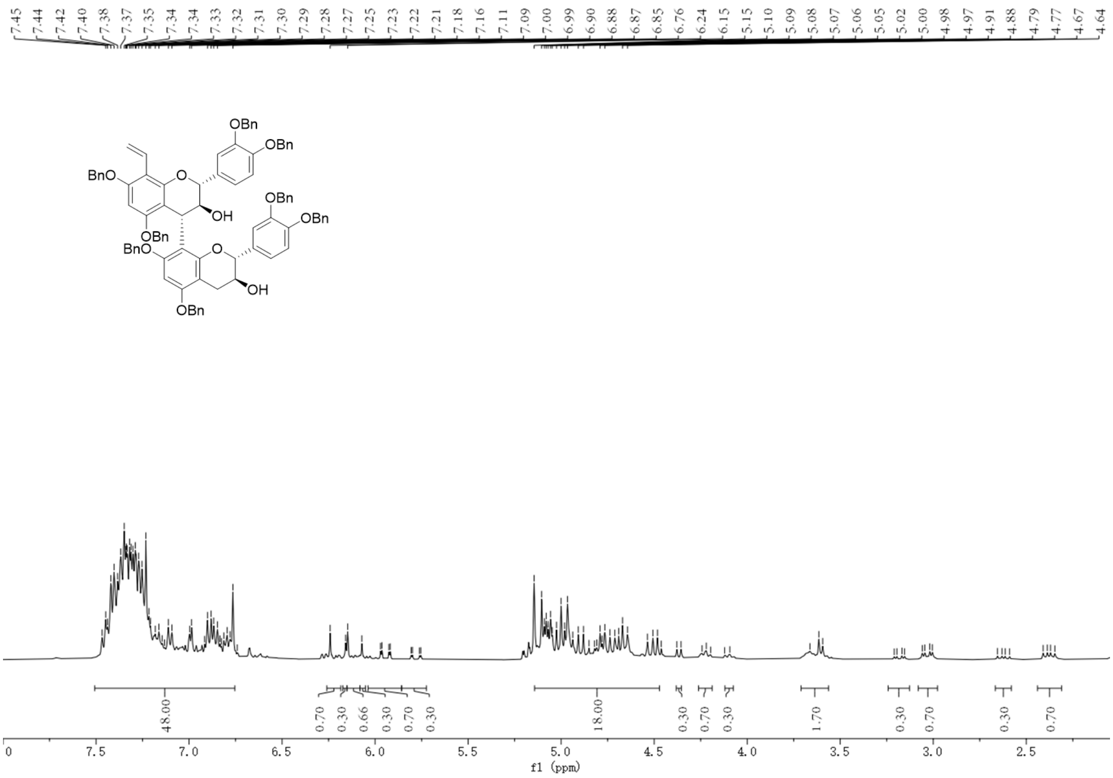

^{1}H-NMR spectrum of compound **5b** (400 MHz, $CDCl_3$)

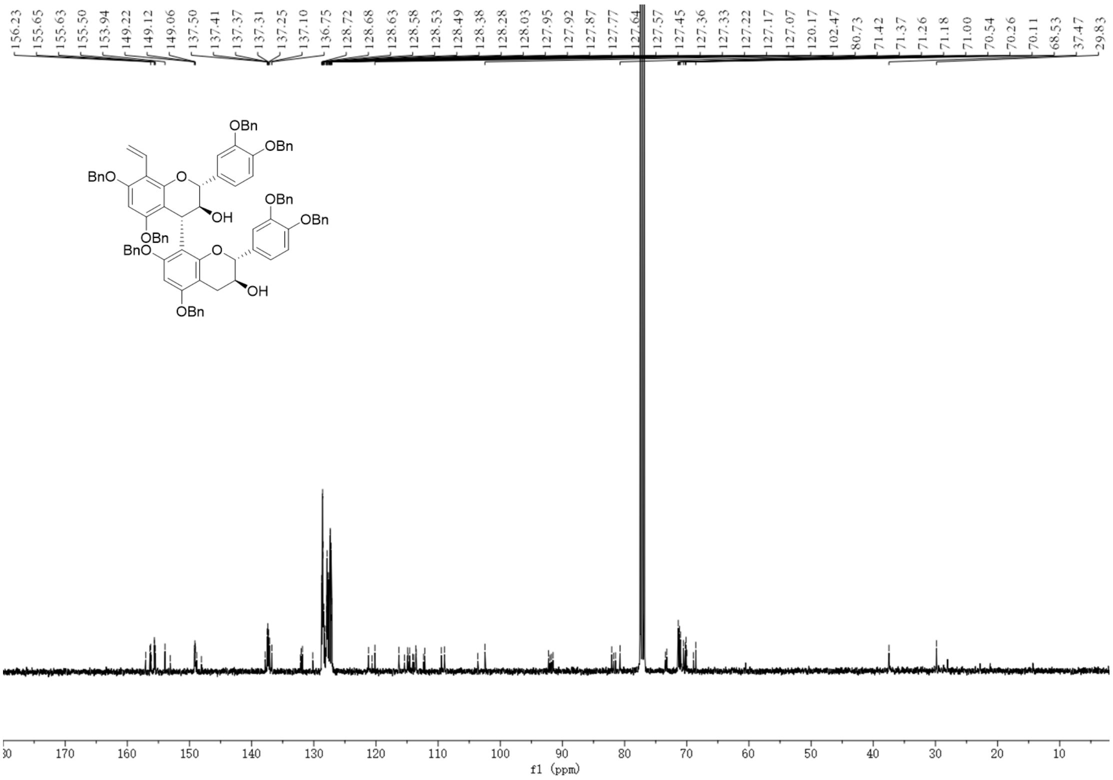

^{13}C-NMR spectrum of compound **5b** (100 MHz, $CDCl_3$)

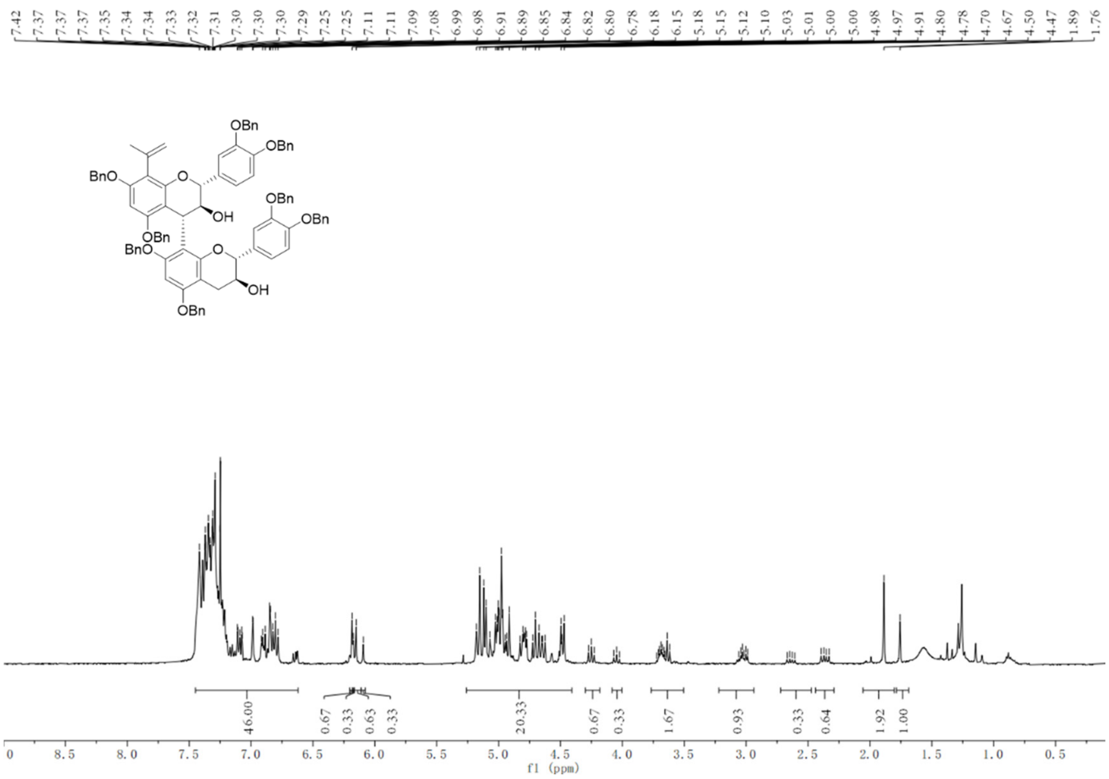

^{1}H-NMR spectrum of compound **5c** (400 MHz, $CDCl_3$)

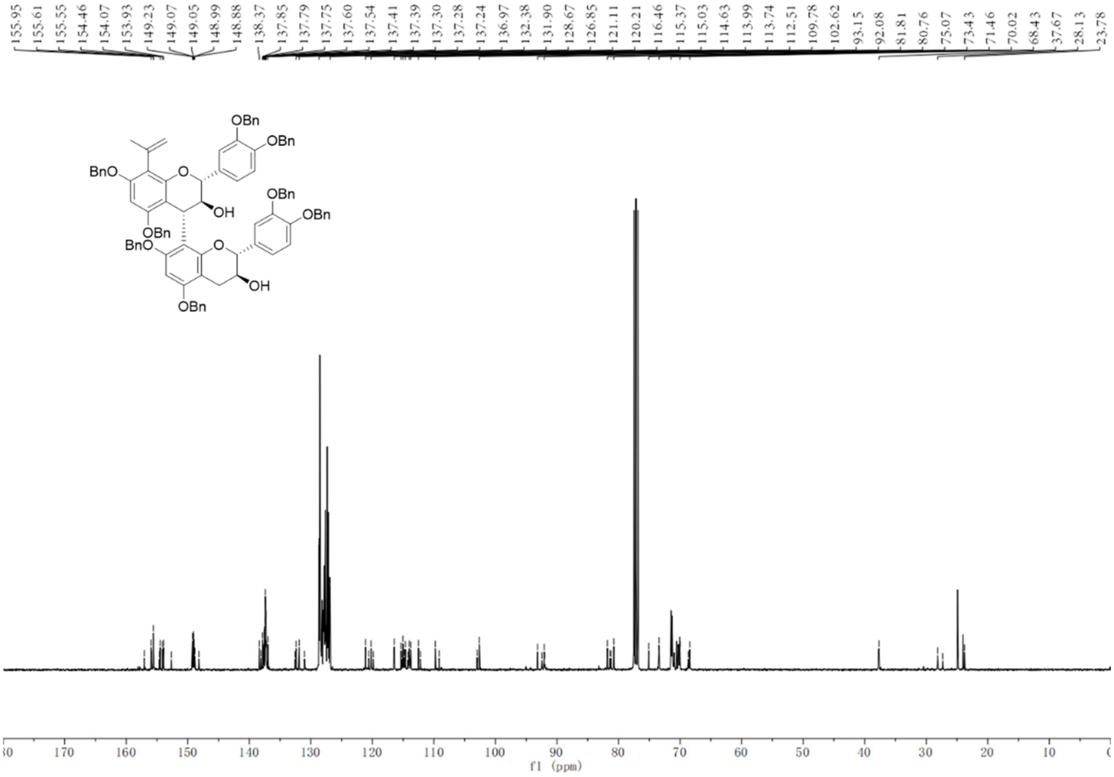

^{13}C-NMR spectrum of compound **5c** (100 MHz, $CDCl_3$)

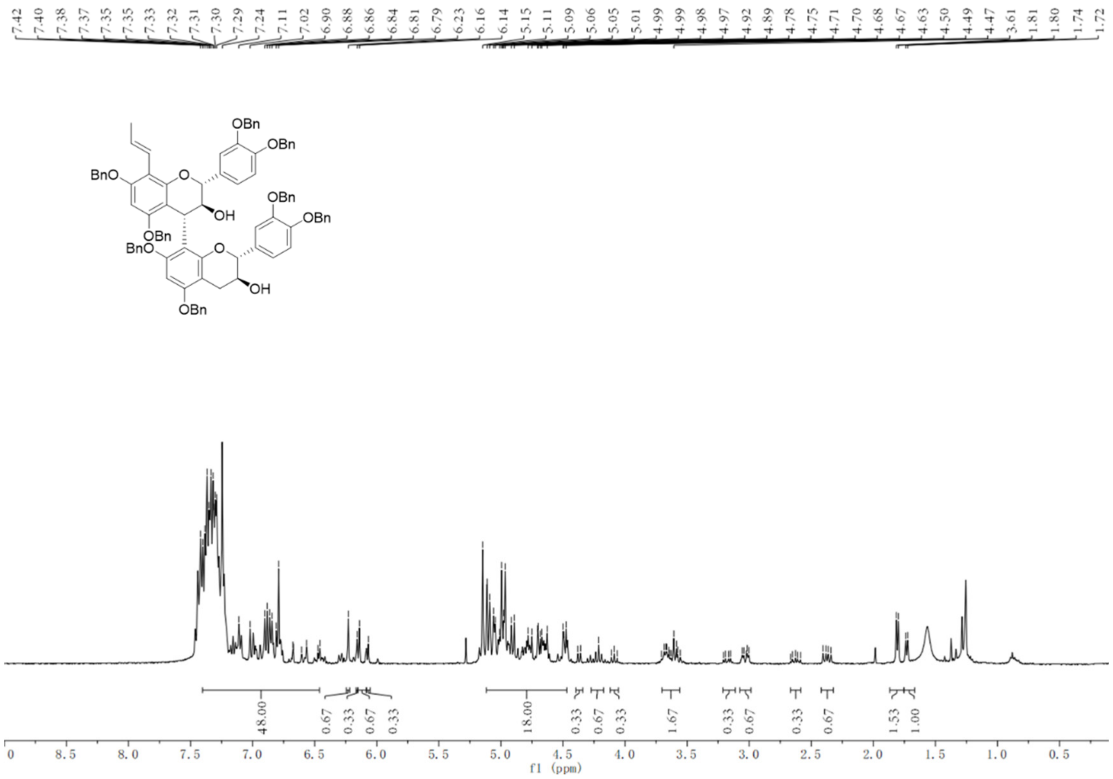

^{1}H-NMR spectrum of compound **5d** (400 MHz, $CDCl_3$)

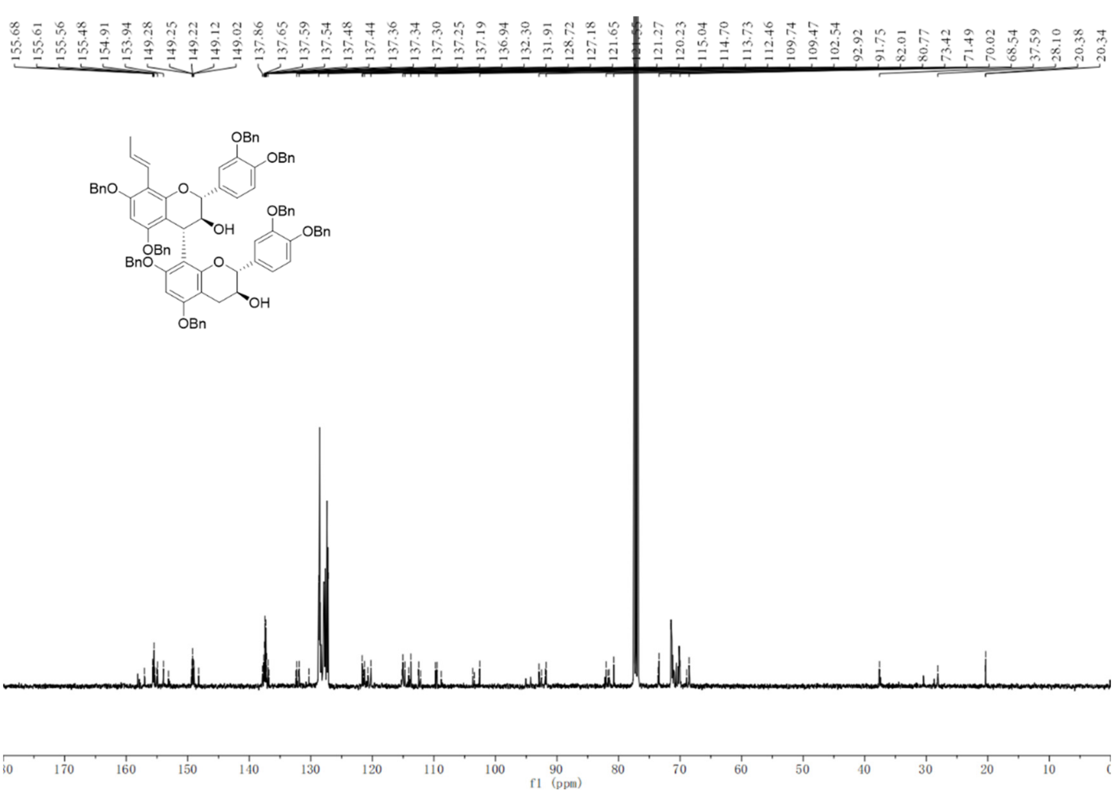

^{13}C-NMR spectrum of compound **5d** (100 MHz, $CDCl_3$)

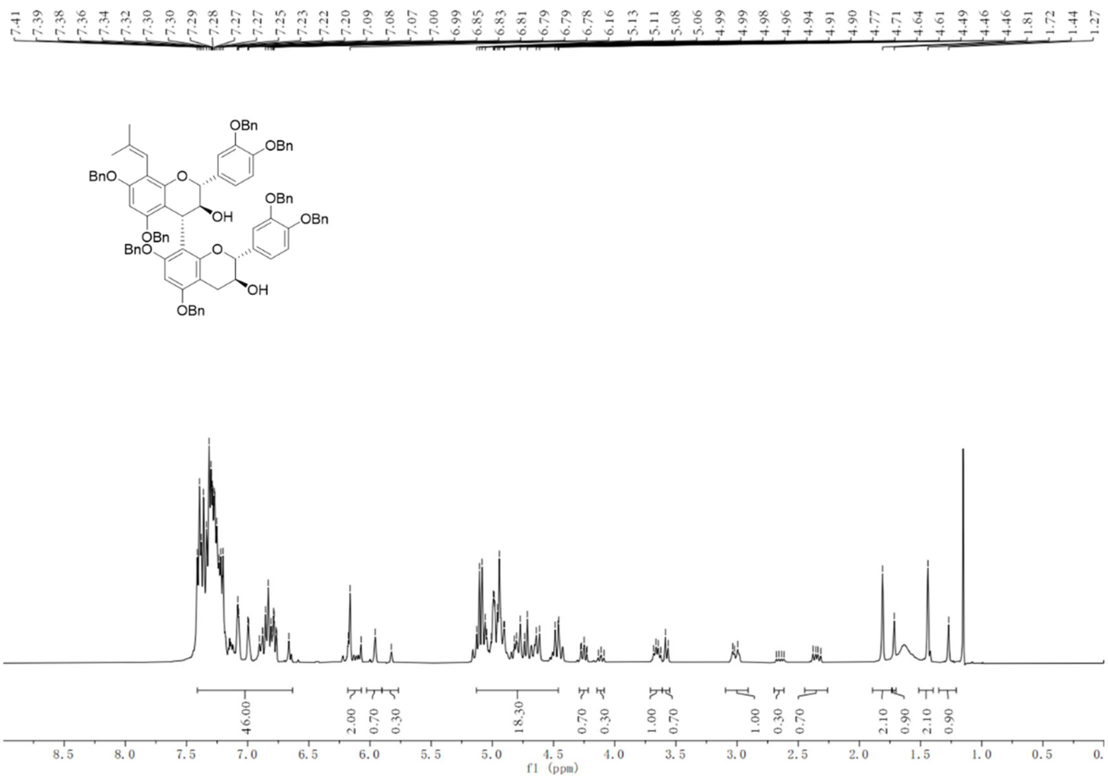

^{1}H-NMR spectrum of compound **5e** (400 MHz, $CDCl_3$)

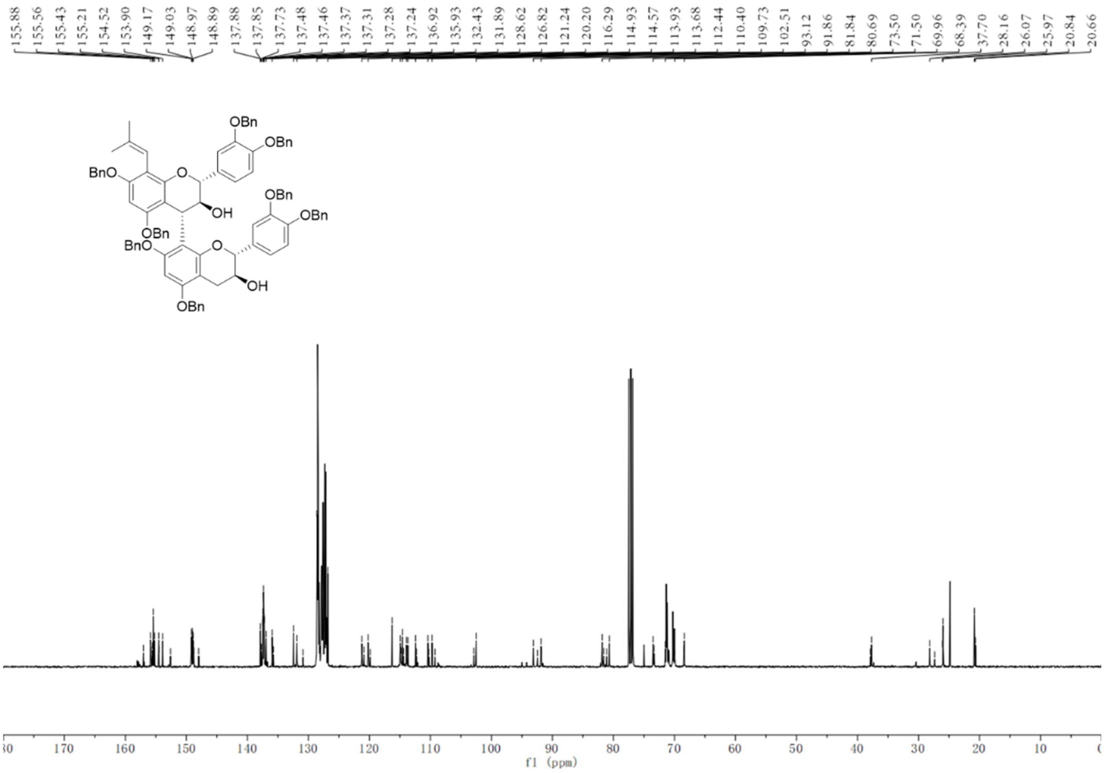

^{13}C-NMR spectrum of compound **5e** (100 MHz, $CDCl_3$)

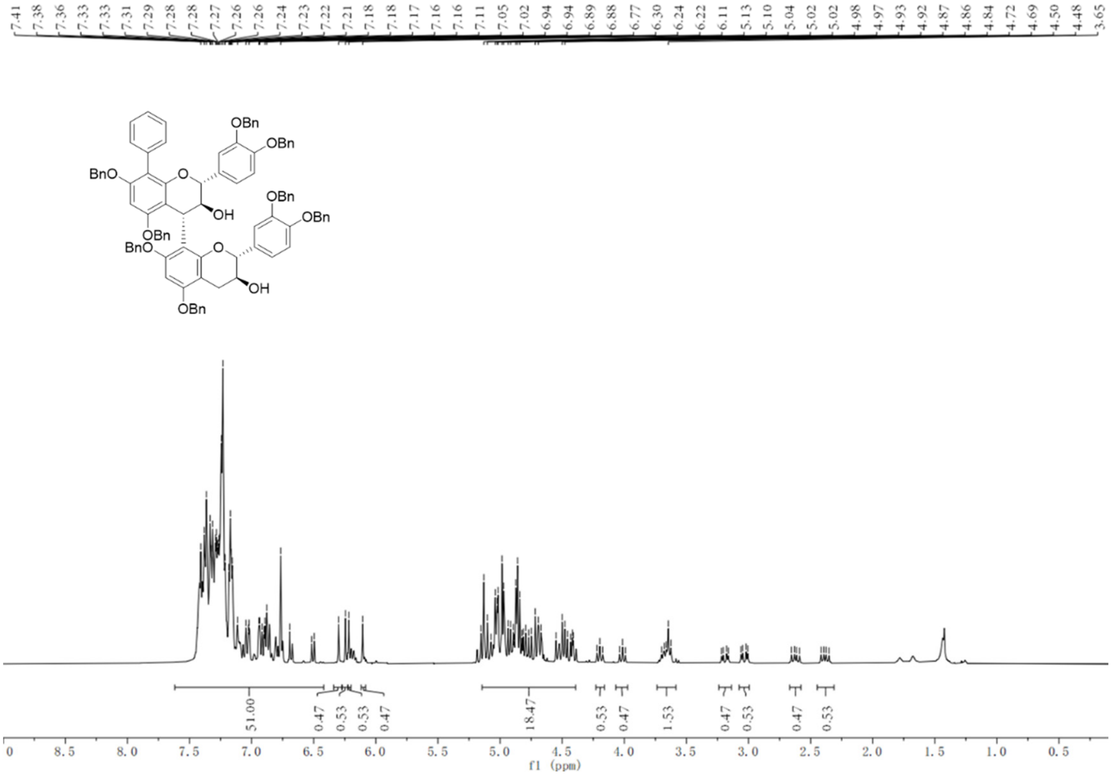

^{1}H-NMR spectrum of compound **5f** (400 MHz, $CDCl_3$)

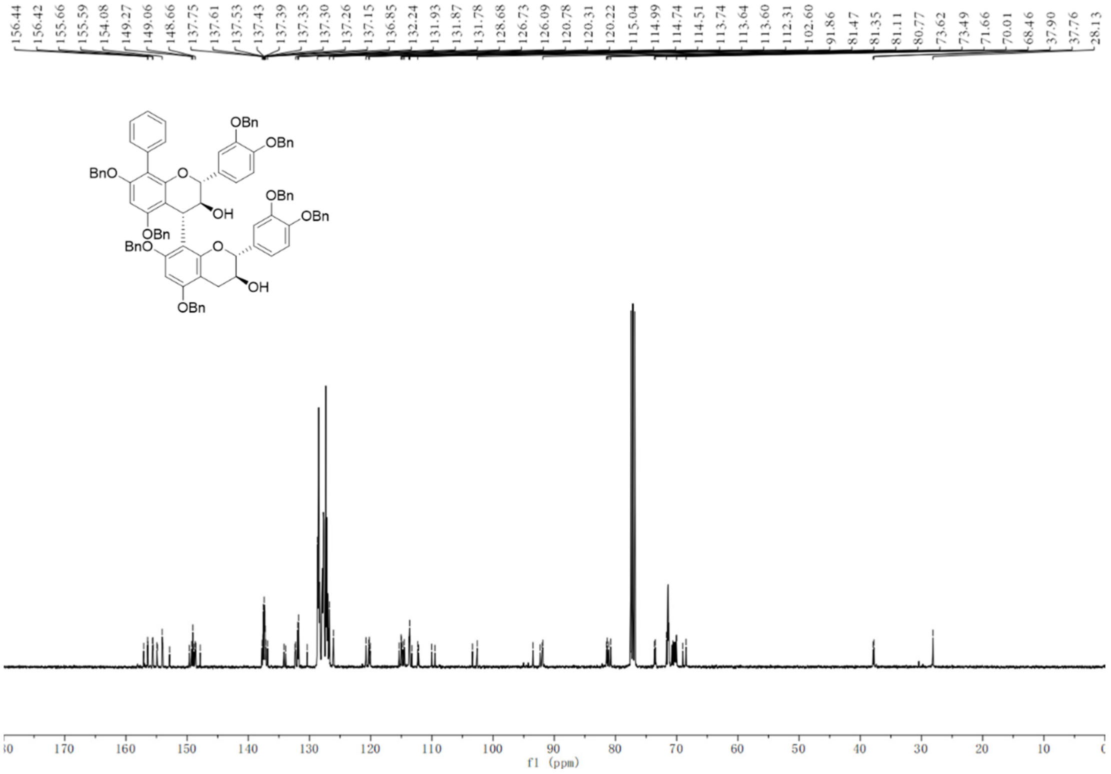

^{13}C-NMR spectrum of compound **5f** (100 MHz, $CDCl_3$)

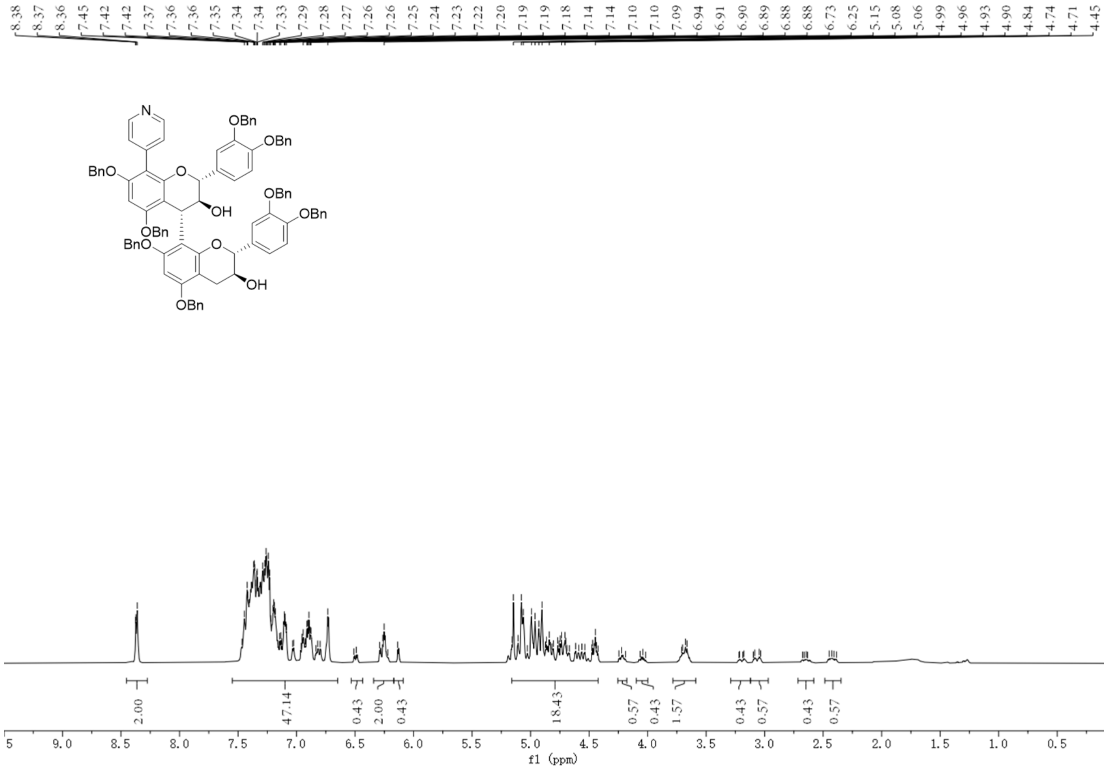

^{1}H-NMR spectrum of compound **5g** (400 MHz, $CDCl_3$)

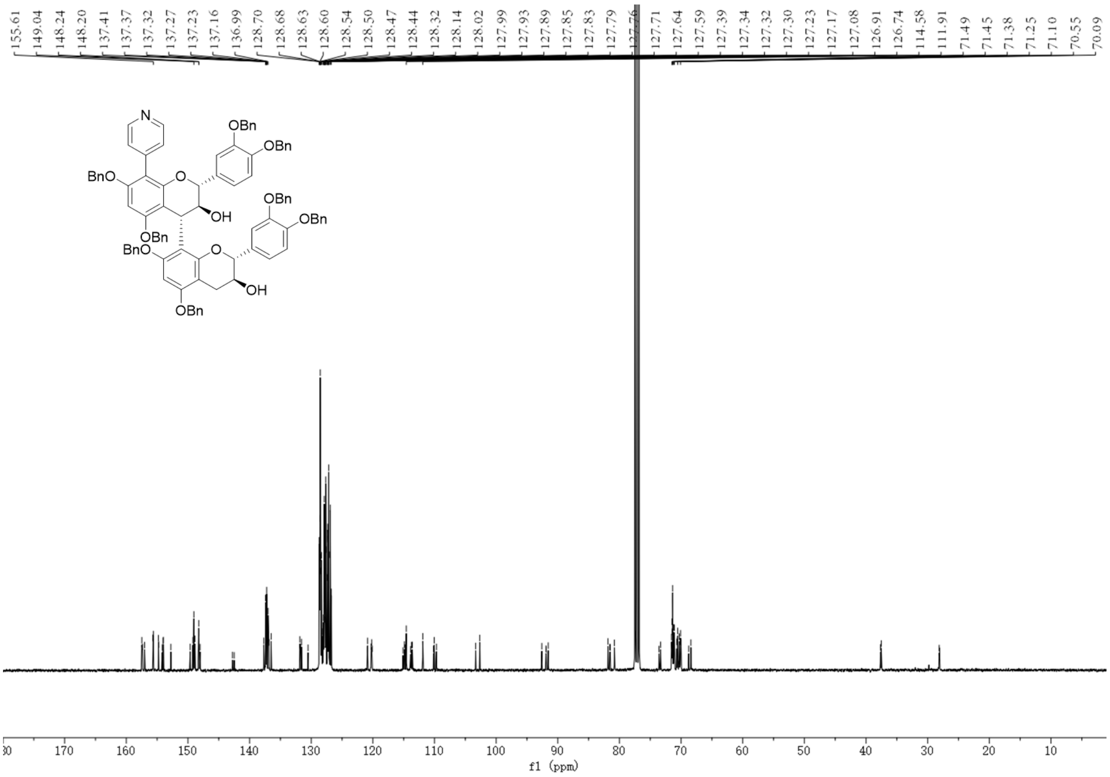

^{13}C-NMR spectrum of compound **5g** (100 MHz, $CDCl_3$)

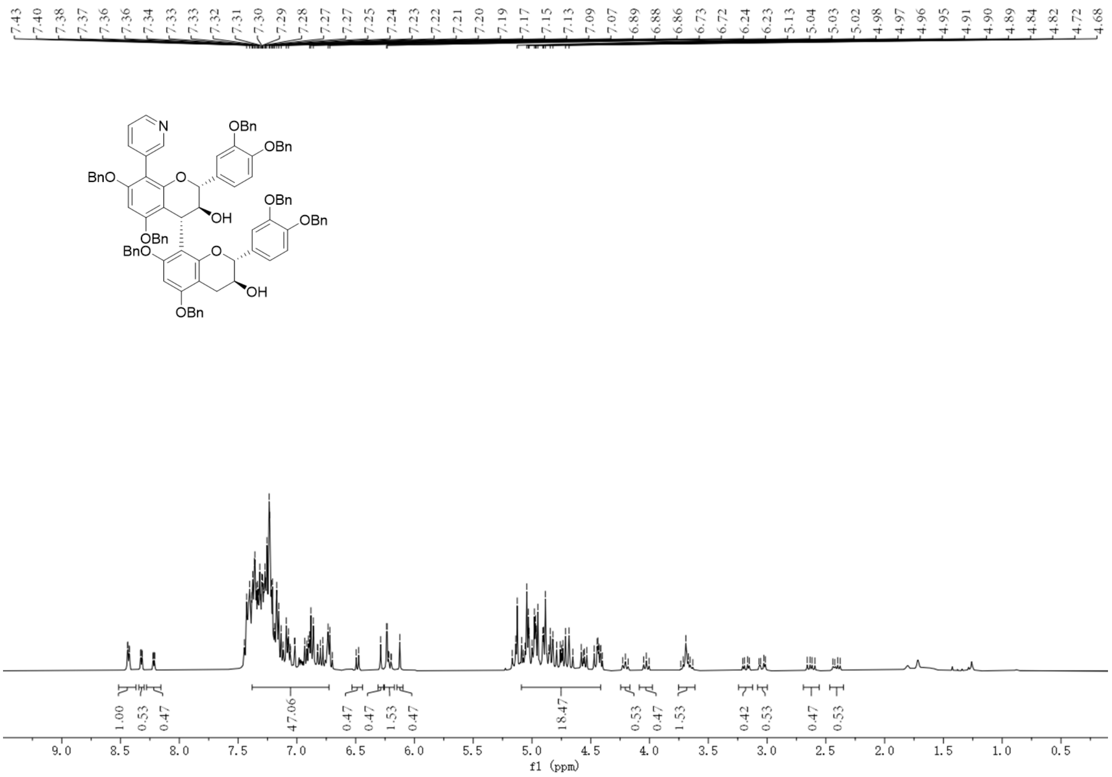

^{1}H-NMR spectrum of compound **5h** (400 MHz, $CDCl_3$)

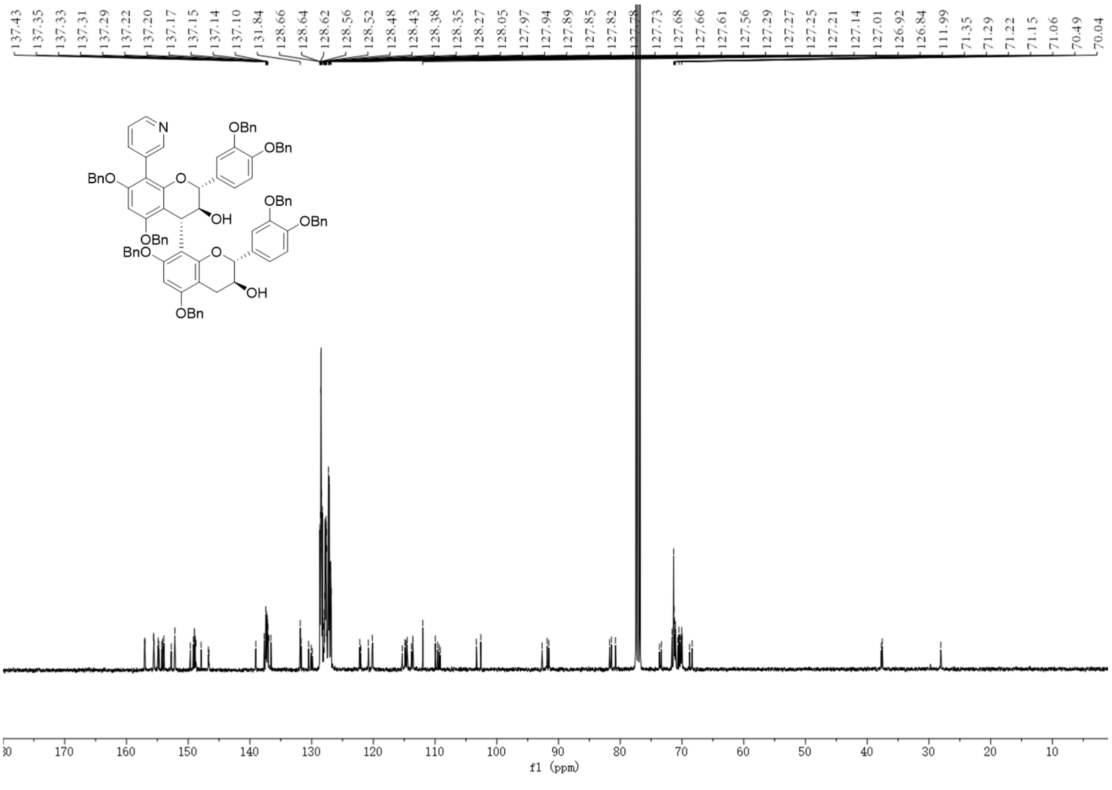

^{13}C-NMR spectrum of compound **5h** (100 MHz, $CDCl_3$)

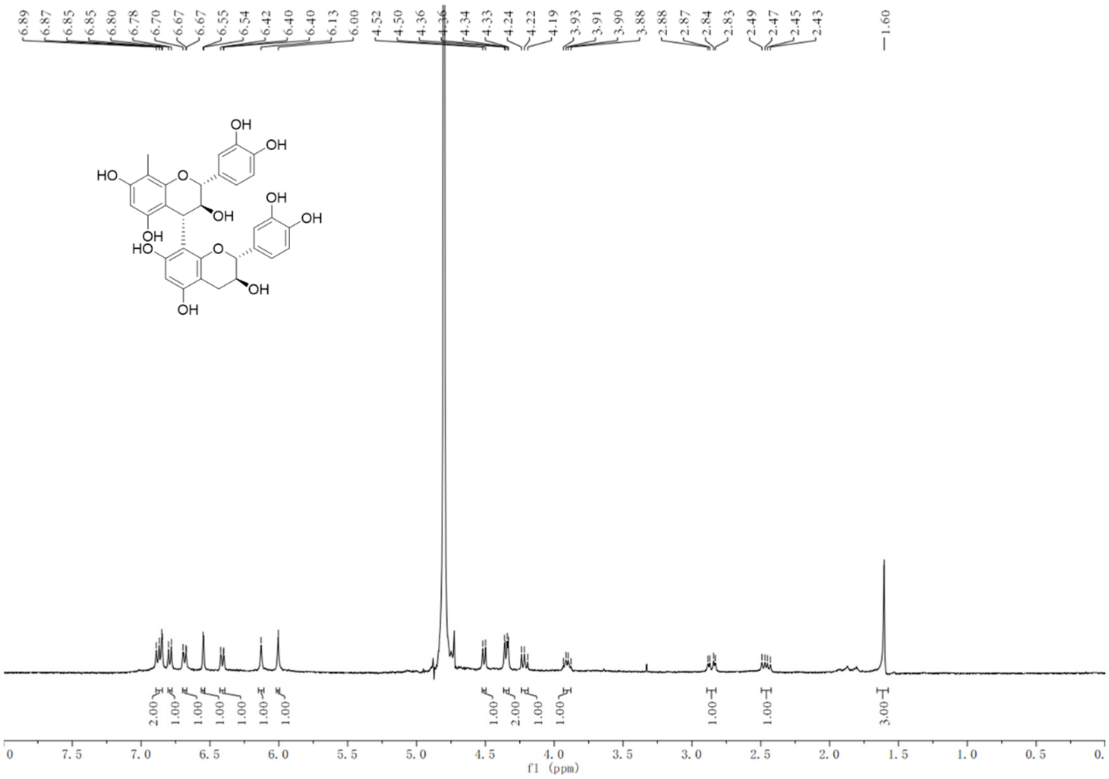

^{1}H-NMR spectrum of compound **6a** (400 MHz, D_2O)

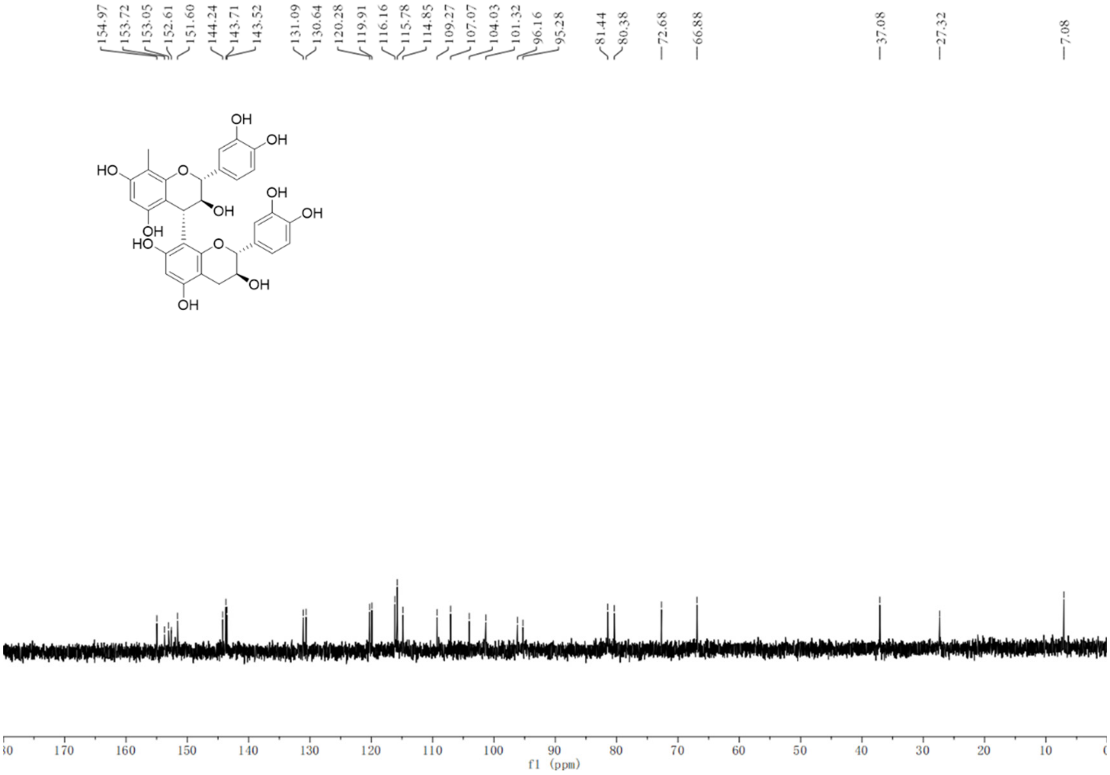

^{13}C-NMR spectrum of compound **6a** (100 MHz, D_2O)

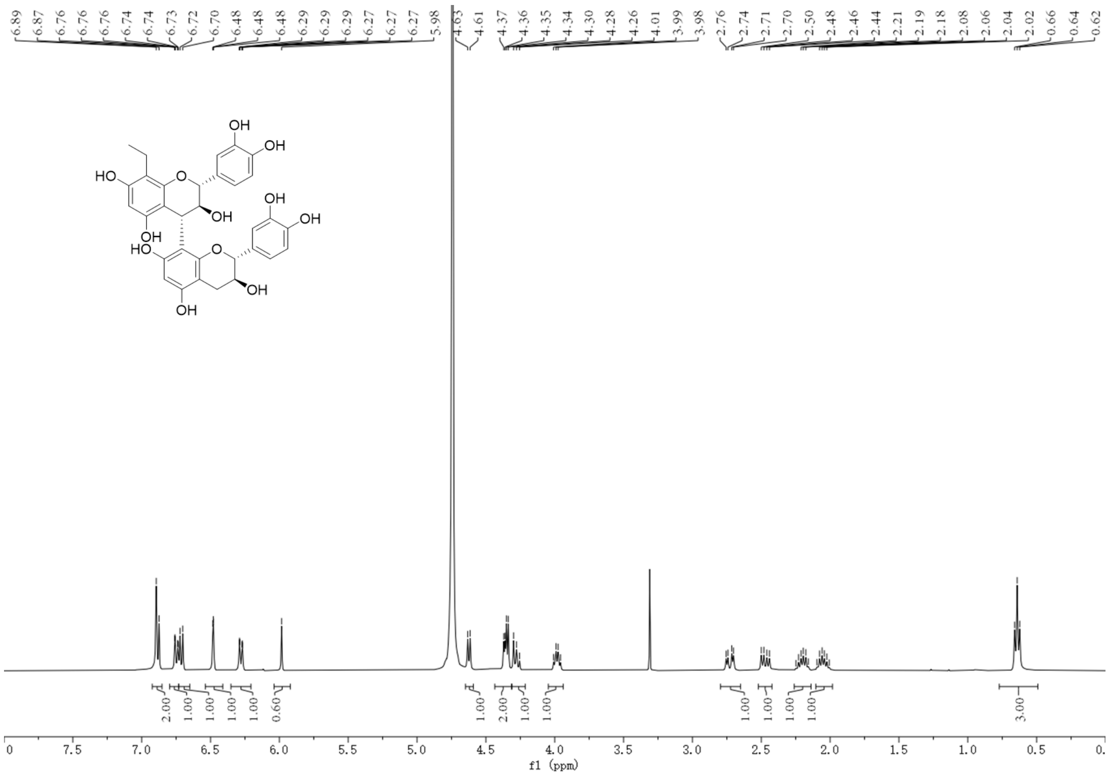

^{1}H-NMR spectrum of compound **6b** (400 MHz, D_2O)

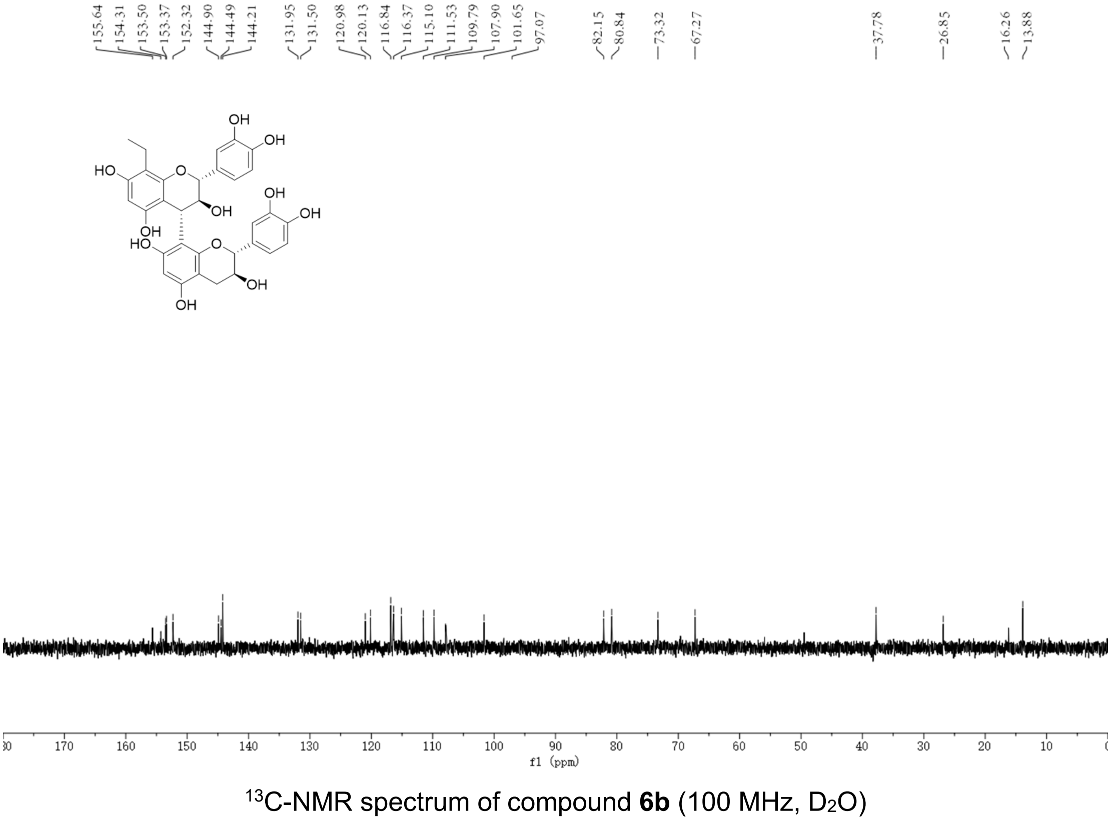

^{13}C-NMR spectrum of compound **6b** (100 MHz, D_2O)

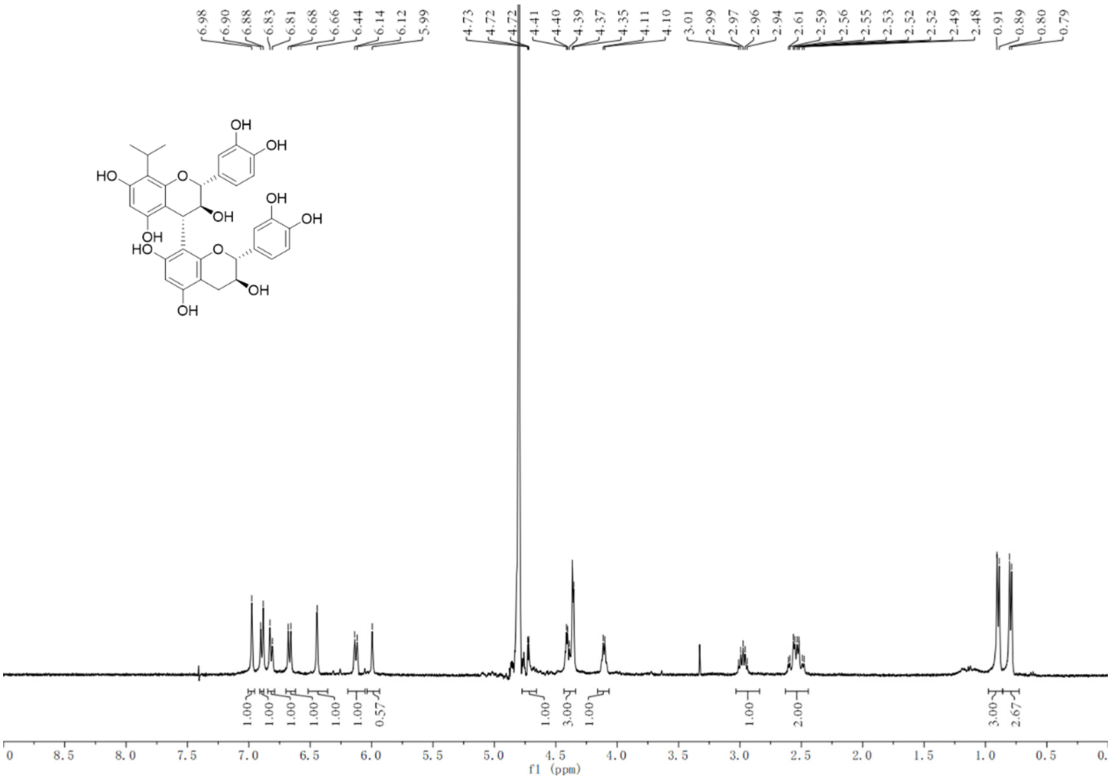

^{1}H-NMR spectrum of compound **6c** (400 MHz, D_2O)

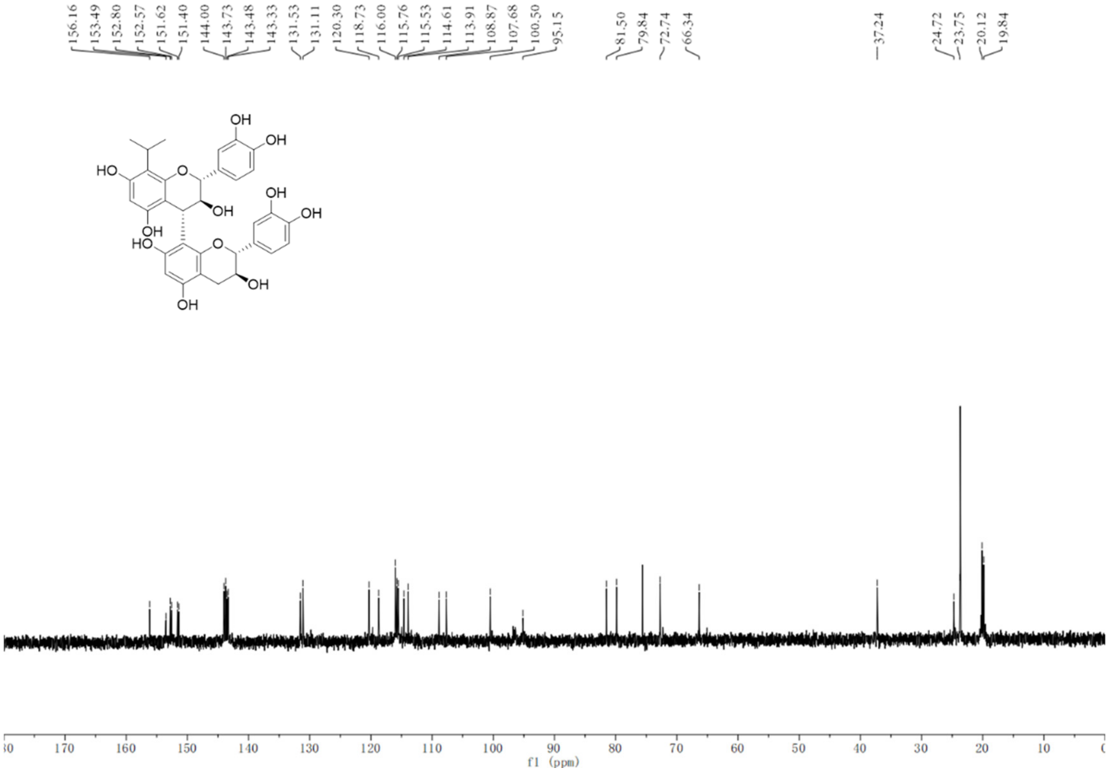

^{13}C-NMR spectrum of compound **6c** (100 MHz, D_2O)

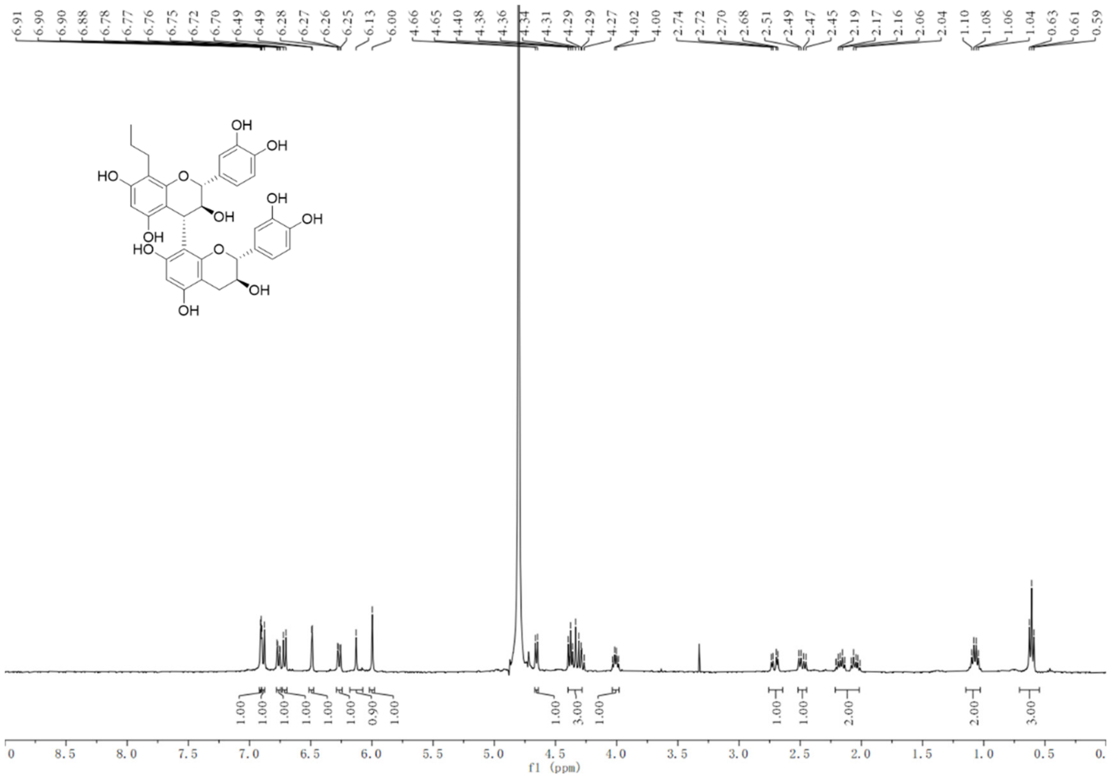

^{1}H-NMR spectrum of compound **6d** (400 MHz, D_2O)

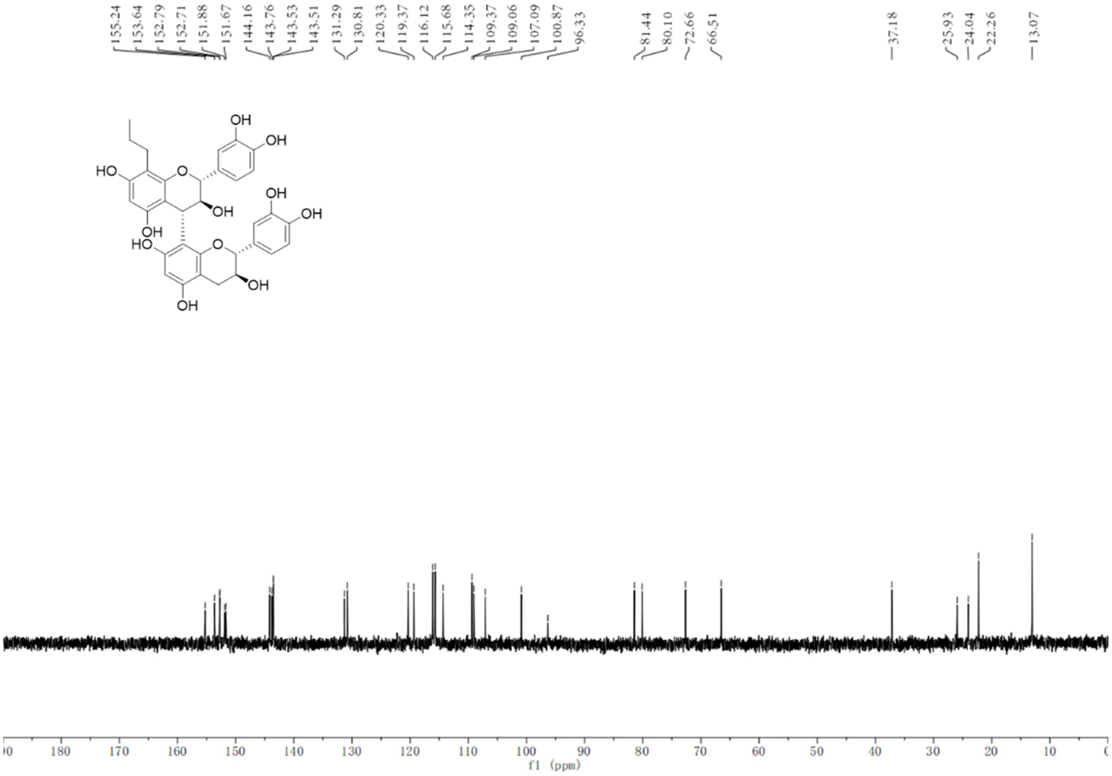

^{13}C-NMR spectrum of compound **6d** (100 MHz, D_2O)

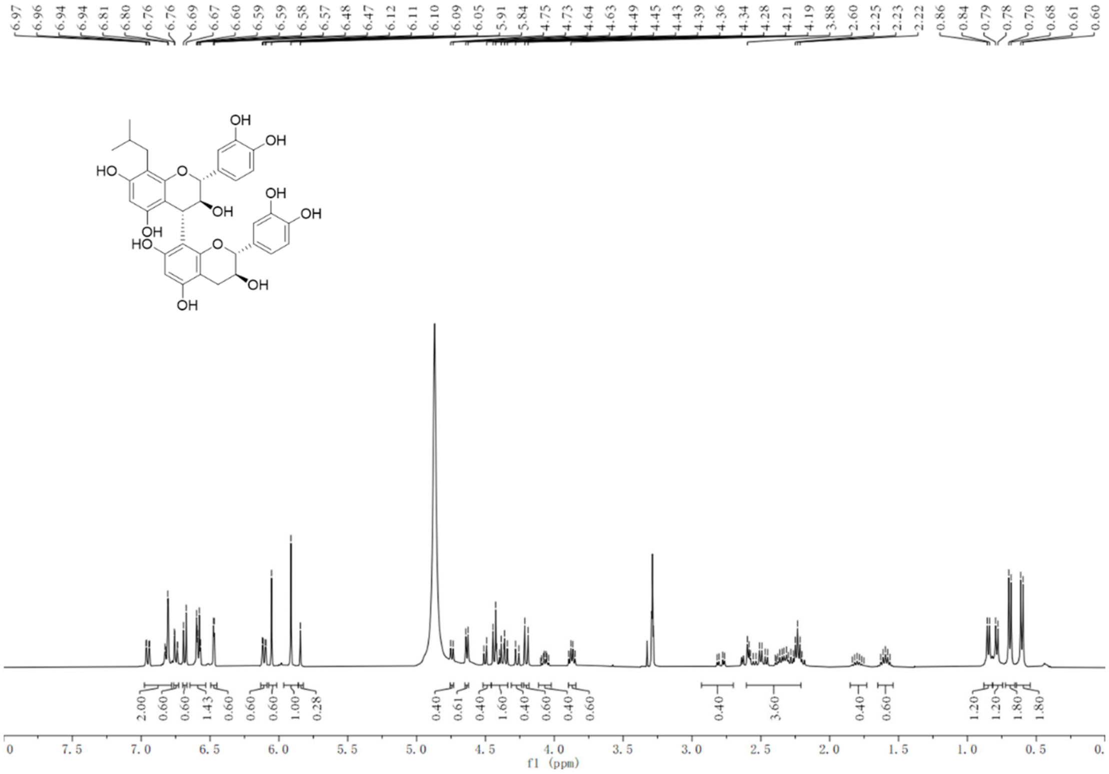

^{1}H-NMR spectrum of compound **6e** (400 MHz, CD_3OD)

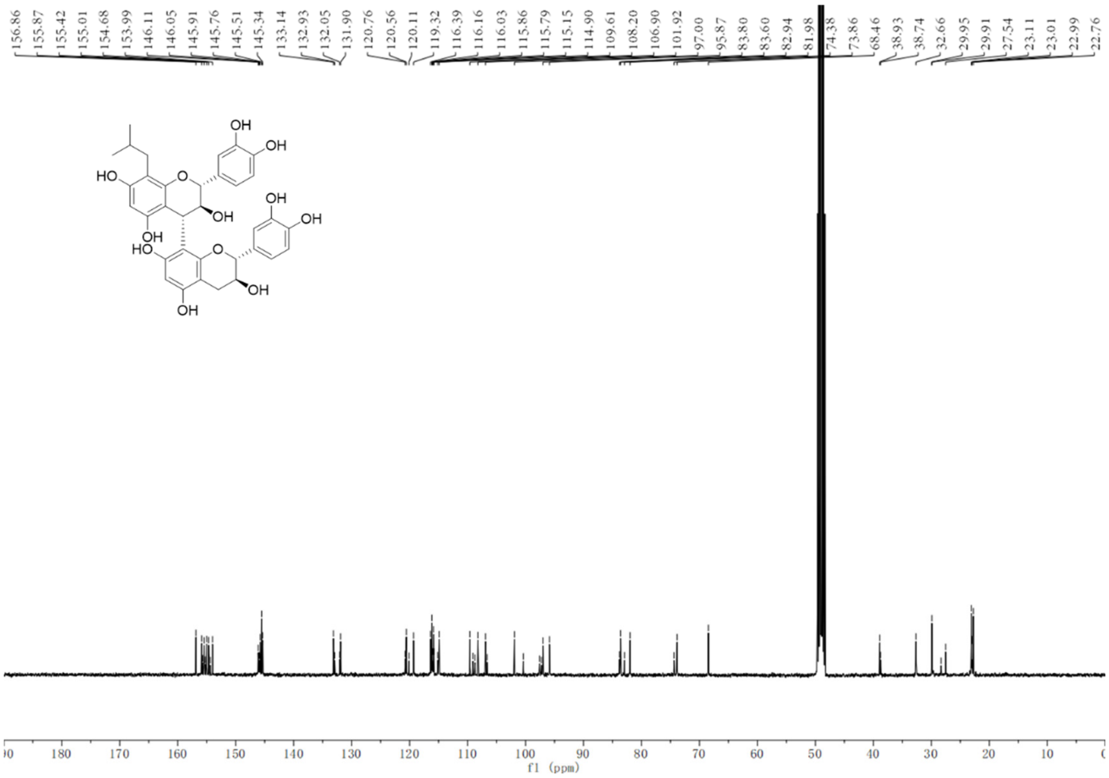

^{13}C-NMR spectrum of compound **6e** (100 MHz, CD_3OD)

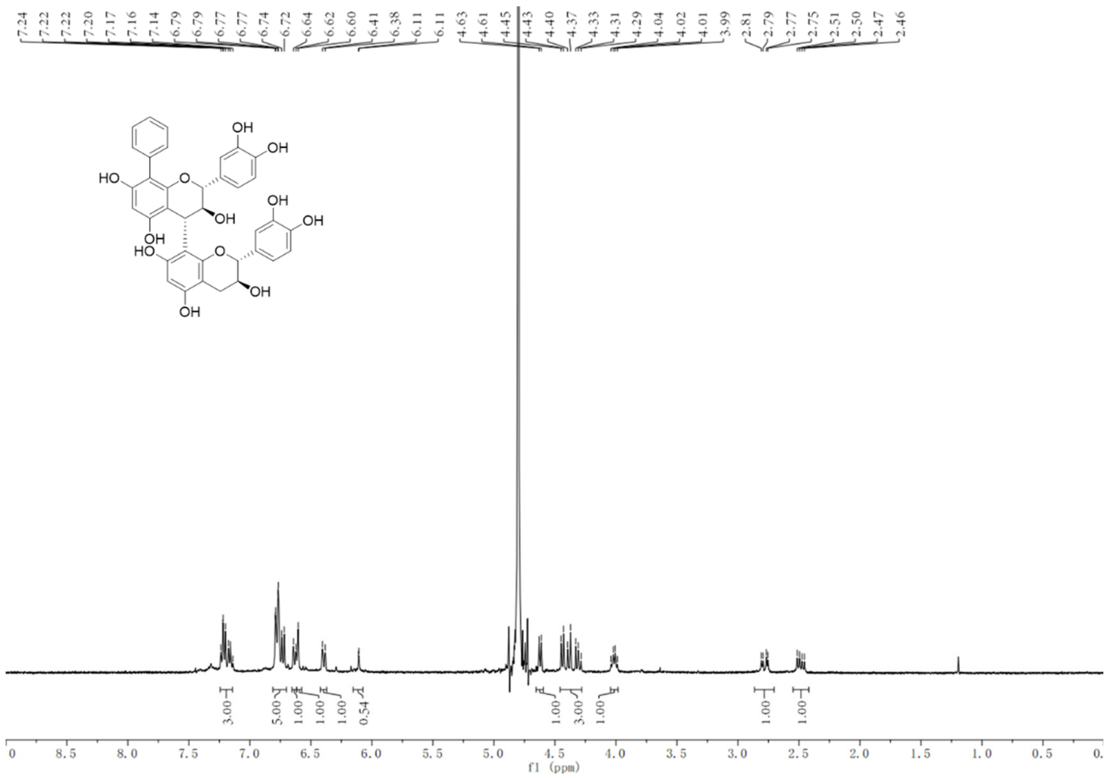

^{1}H-NMR spectrum of compound **6f** (400 MHz, D_2O)

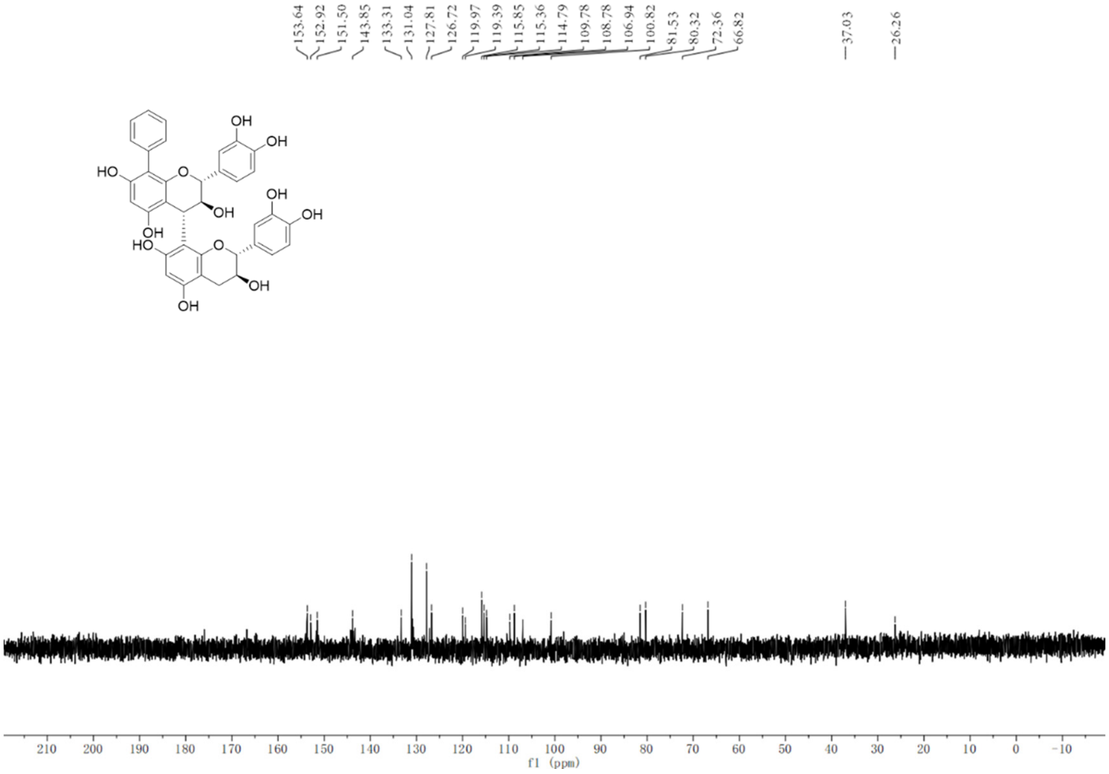

^{13}C-NMR spectrum of compound **6f** (100 MHz, D_2O)

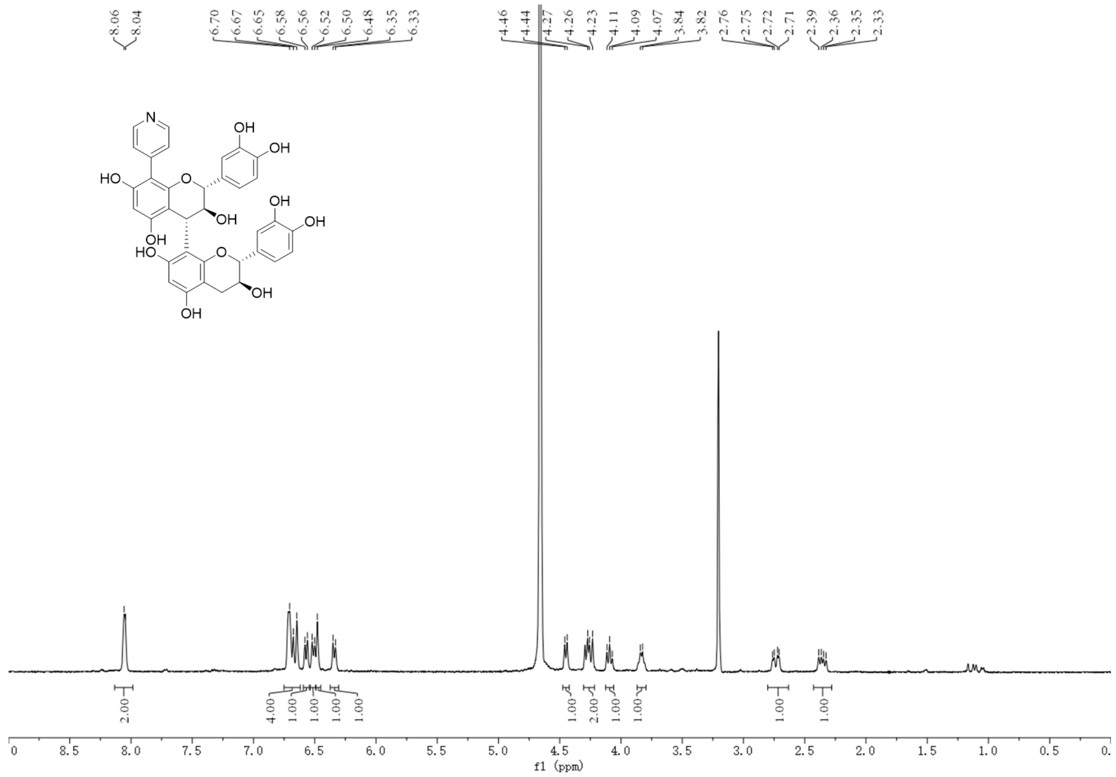

^{1}H-NMR spectrum of compound **6g** (400 MHz, D_2O)

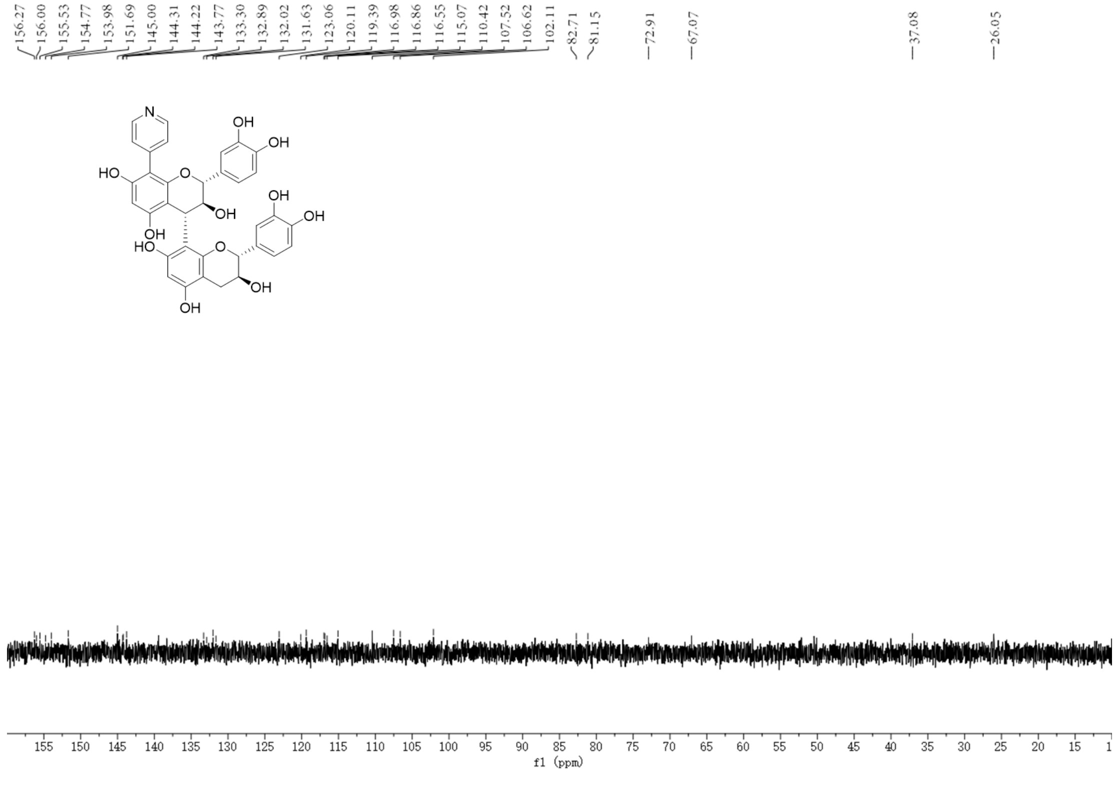

^{13}C-NMR spectrum of compound **6g** (100 MHz, D_2O)

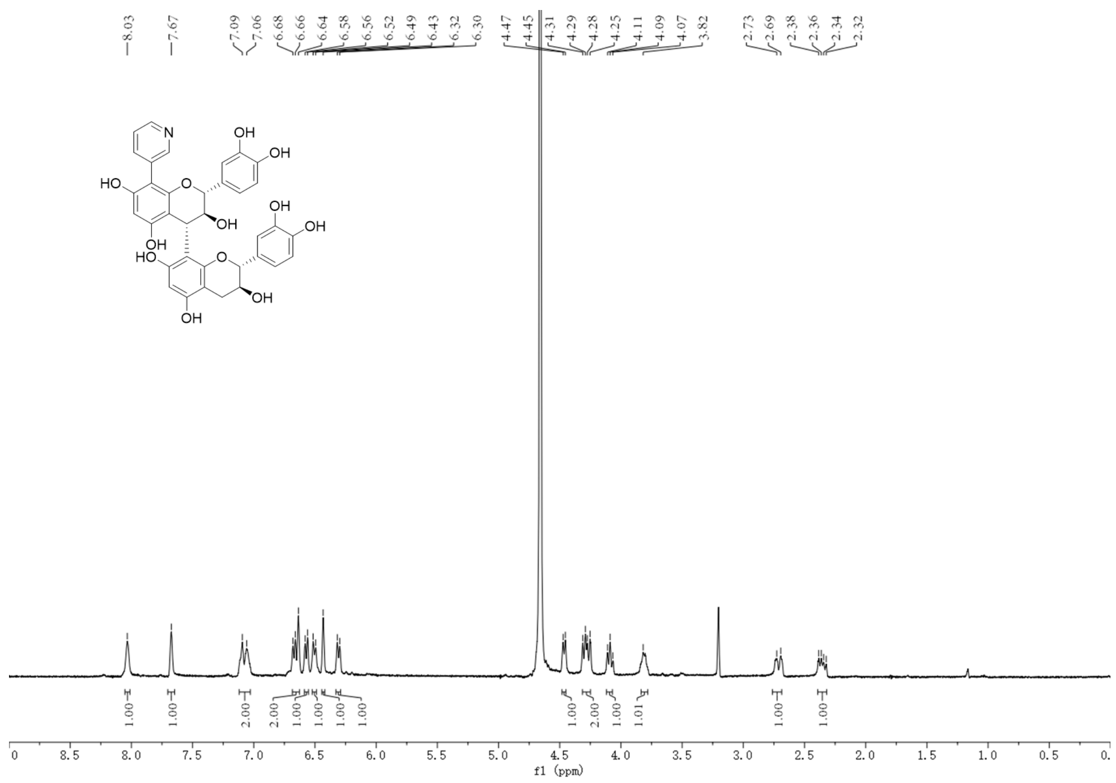

^{1}H-NMR spectrum of compound **6h** (400 MHz, D_2O)

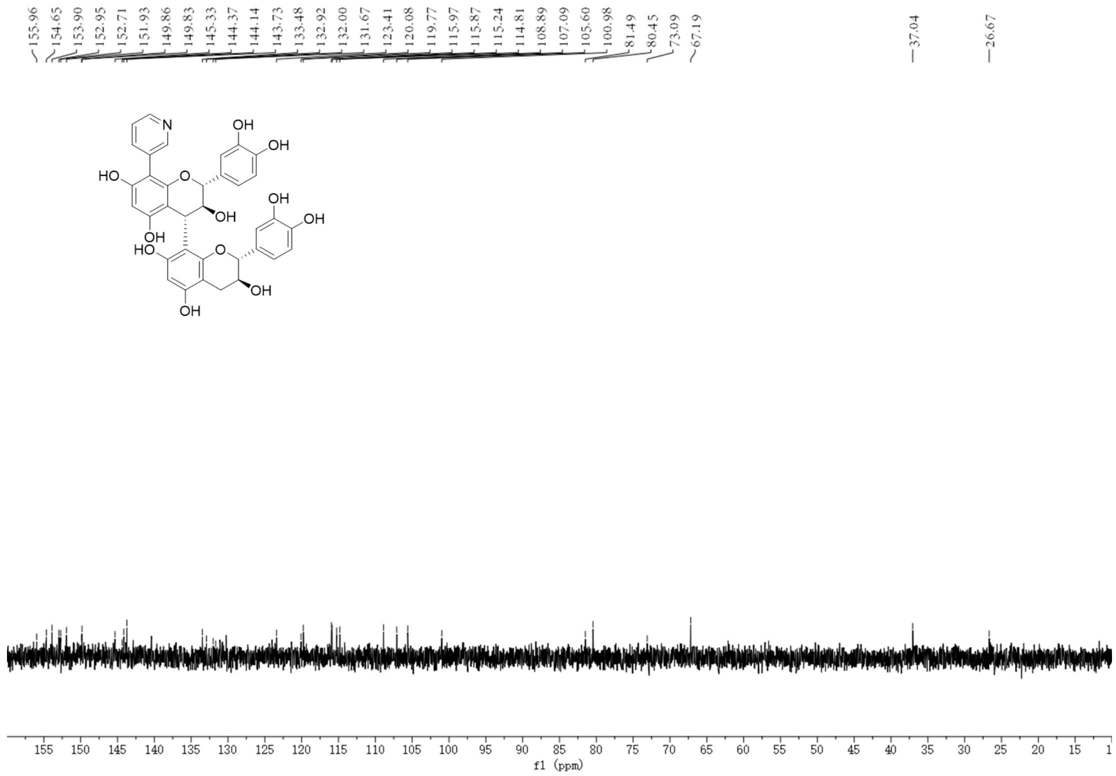

^{13}C-NMR spectrum of compound **6h** (100 MHz, D_2O)

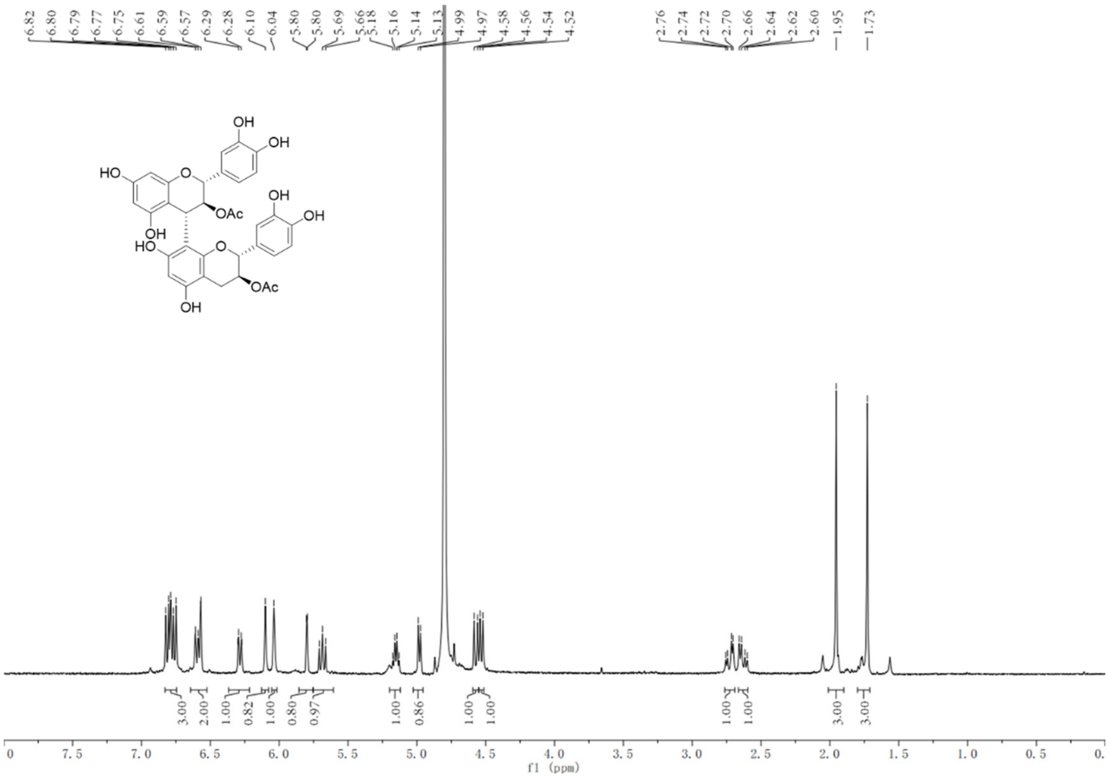

^{1}H-NMR spectrum of compound **8** (400 MHz, D_2O)

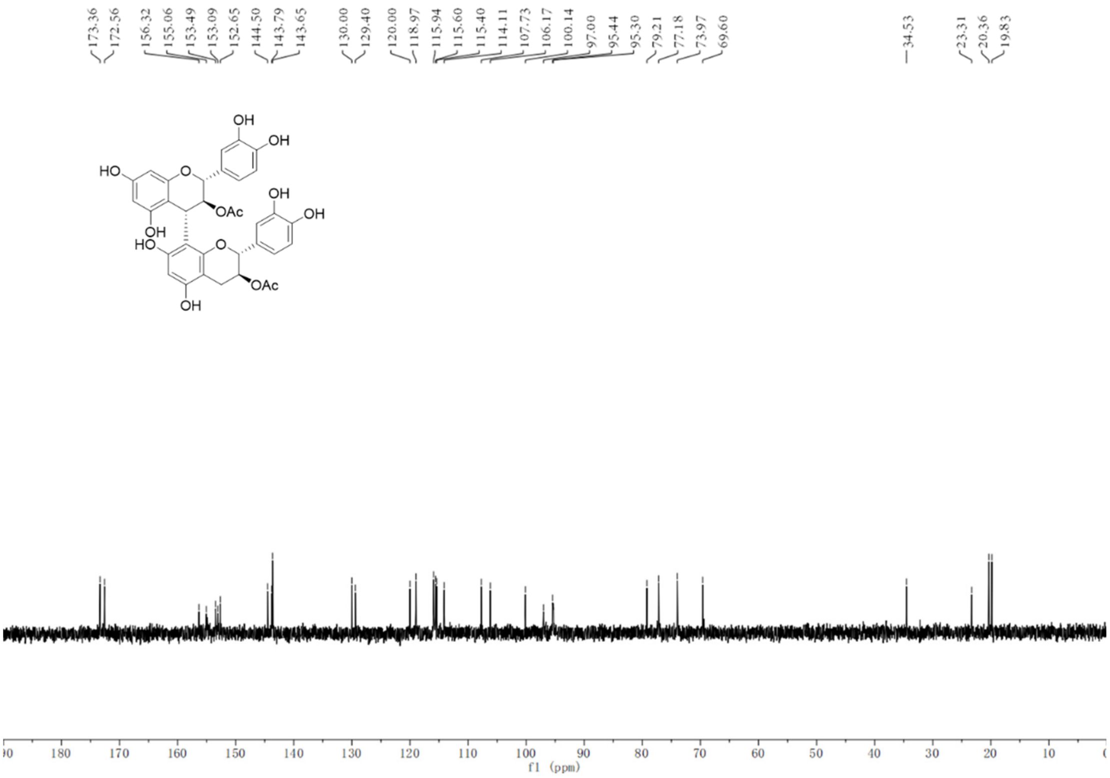

^{13}C-NMR spectrum of compound **8** (100 MHz, D_2O)

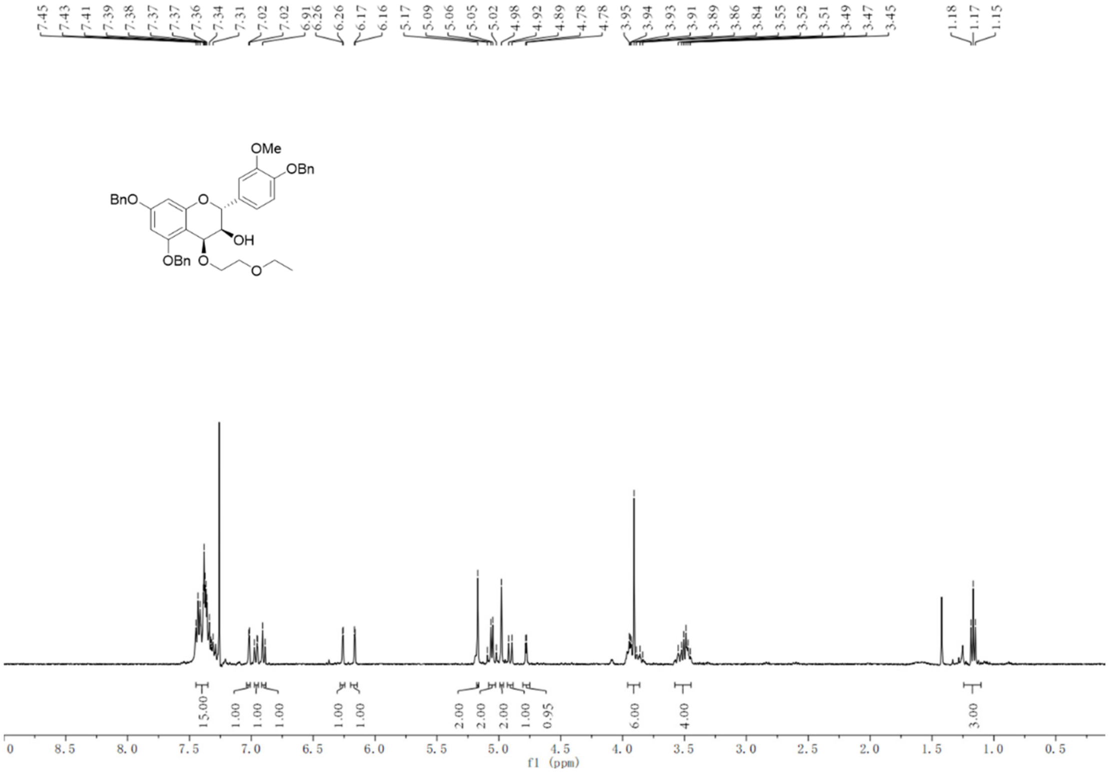

^{1}H-NMR spectrum of compound **12a** (400 MHz, $CDCl_3$)

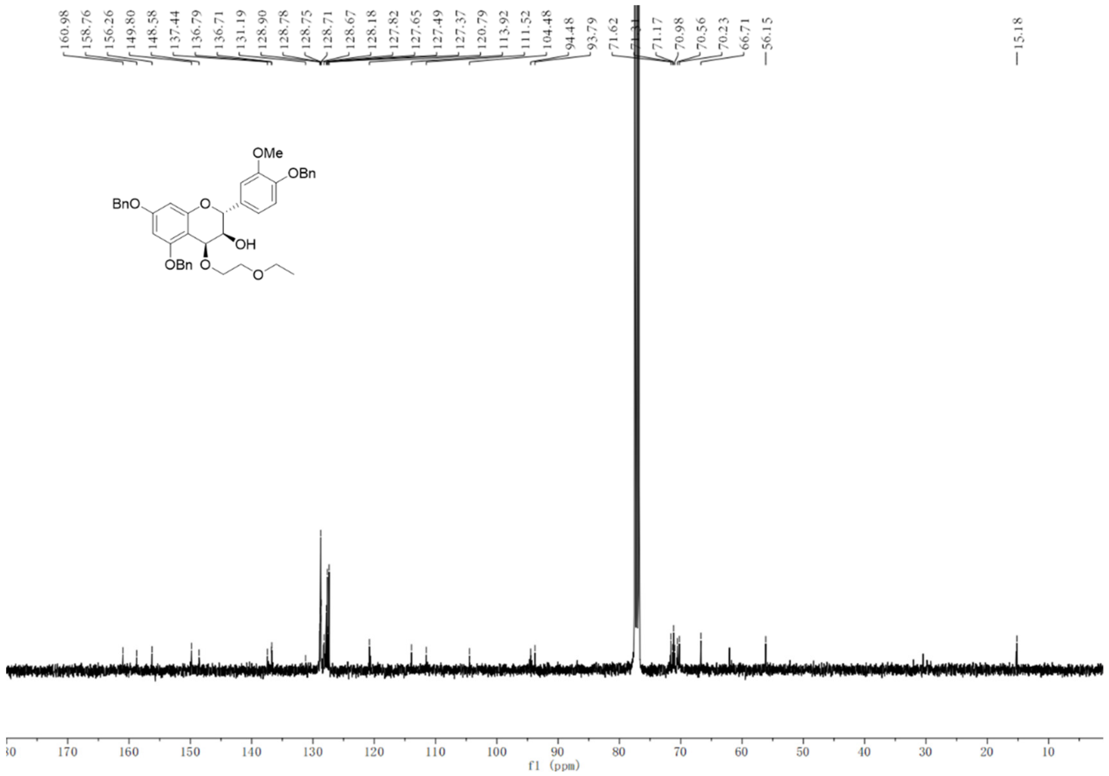

^{13}C-NMR spectrum of compound **12a** (100 MHz, $CDCl_3$)

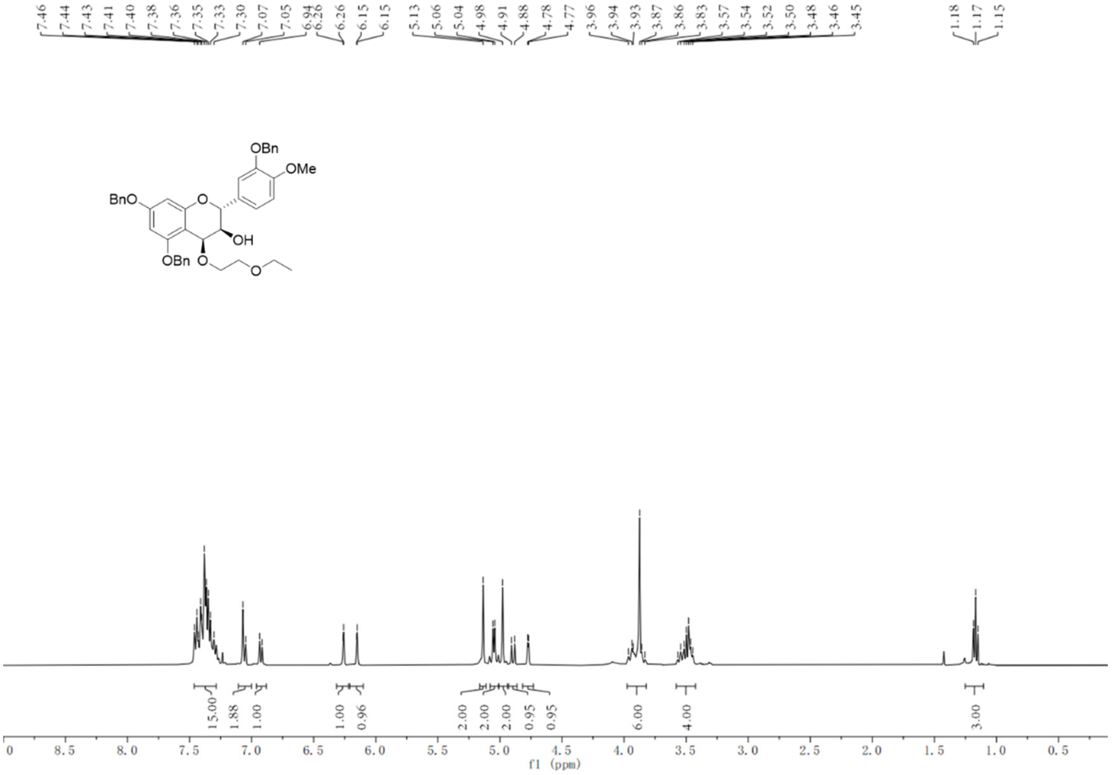

^{1}H-NMR spectrum of compound **12b** (400 MHz, $CDCl_3$)

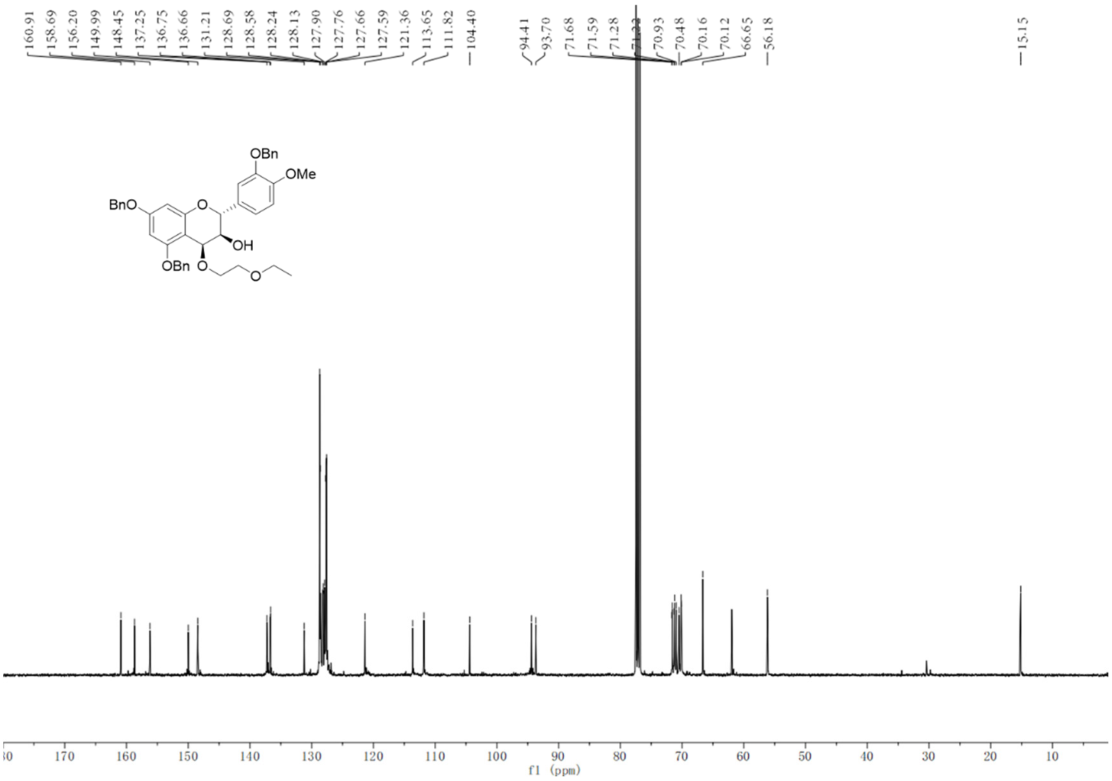

^{13}C-NMR spectrum of compound **12b** (100 MHz, $CDCl_3$)

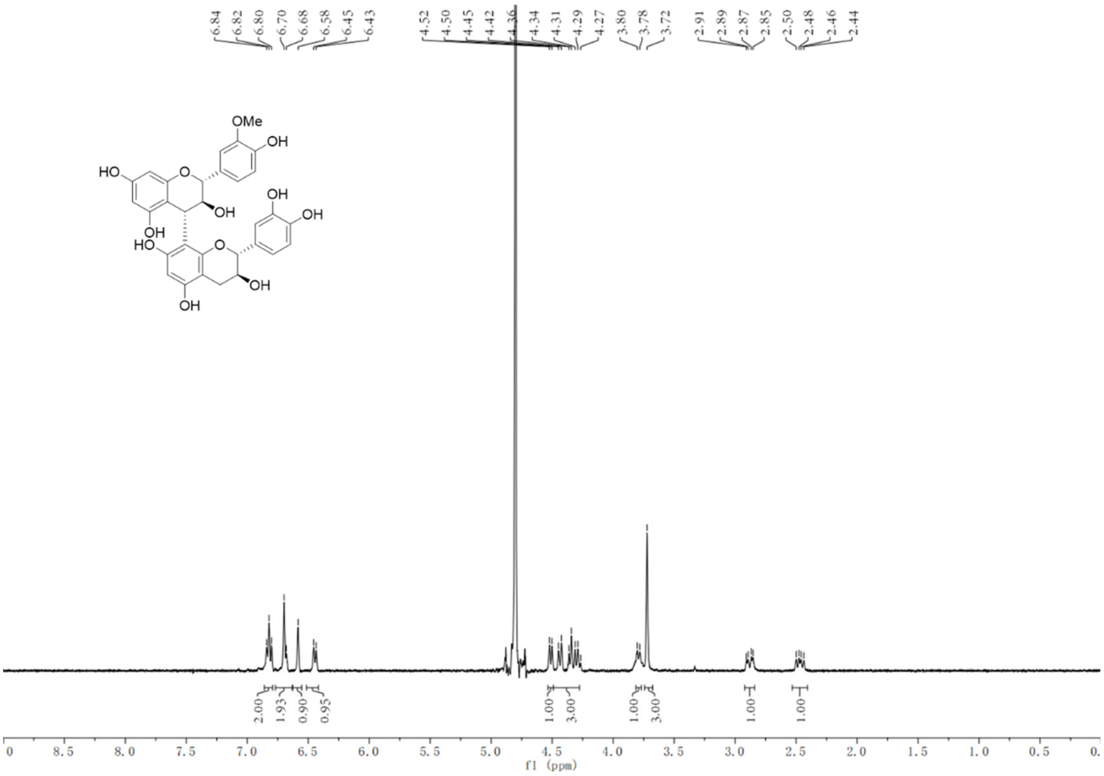

^{1}H-NMR spectrum of compound **13a** (400 MHz, D_2O)

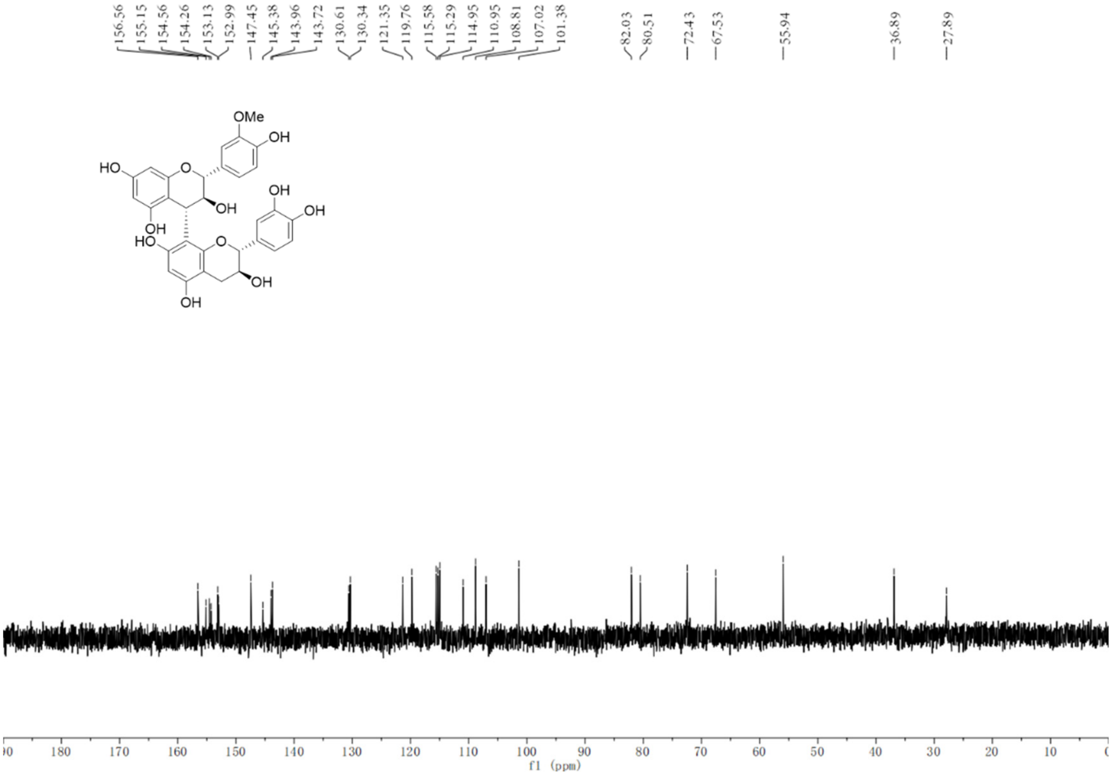

^{13}C-NMR spectrum of compound **13a** (100 MHz, D_2O)

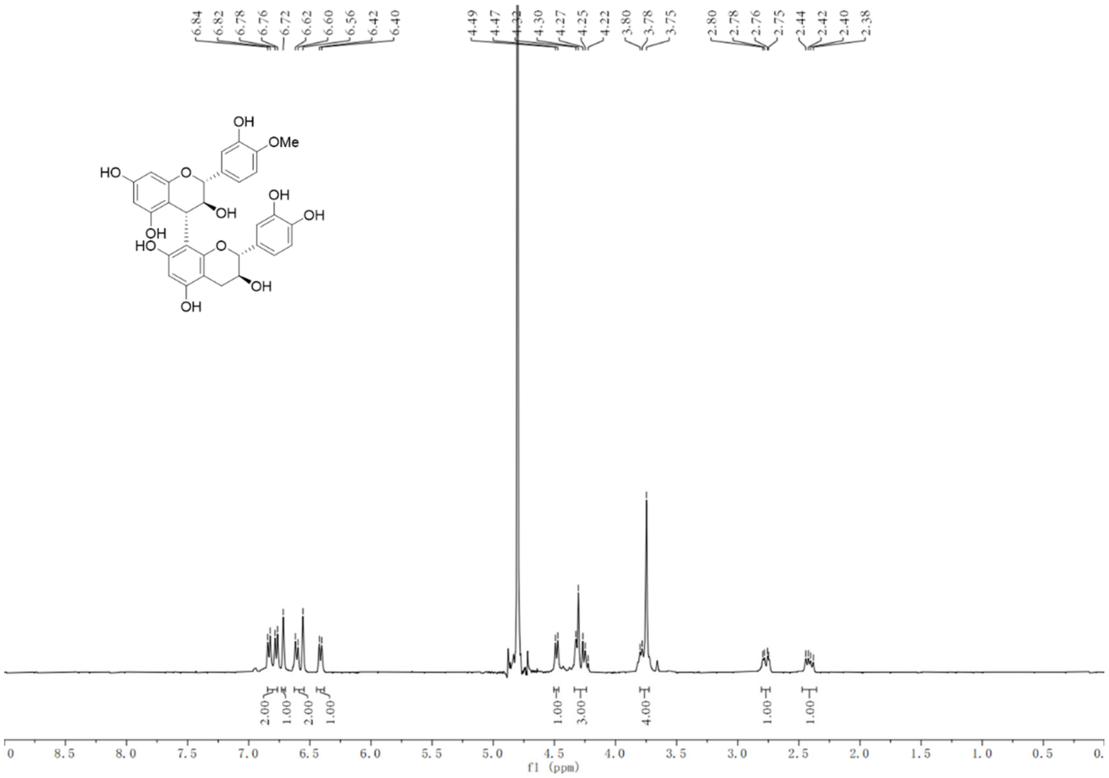

^{1}H-NMR spectrum of compound **13b** (400 MHz, D_2O)

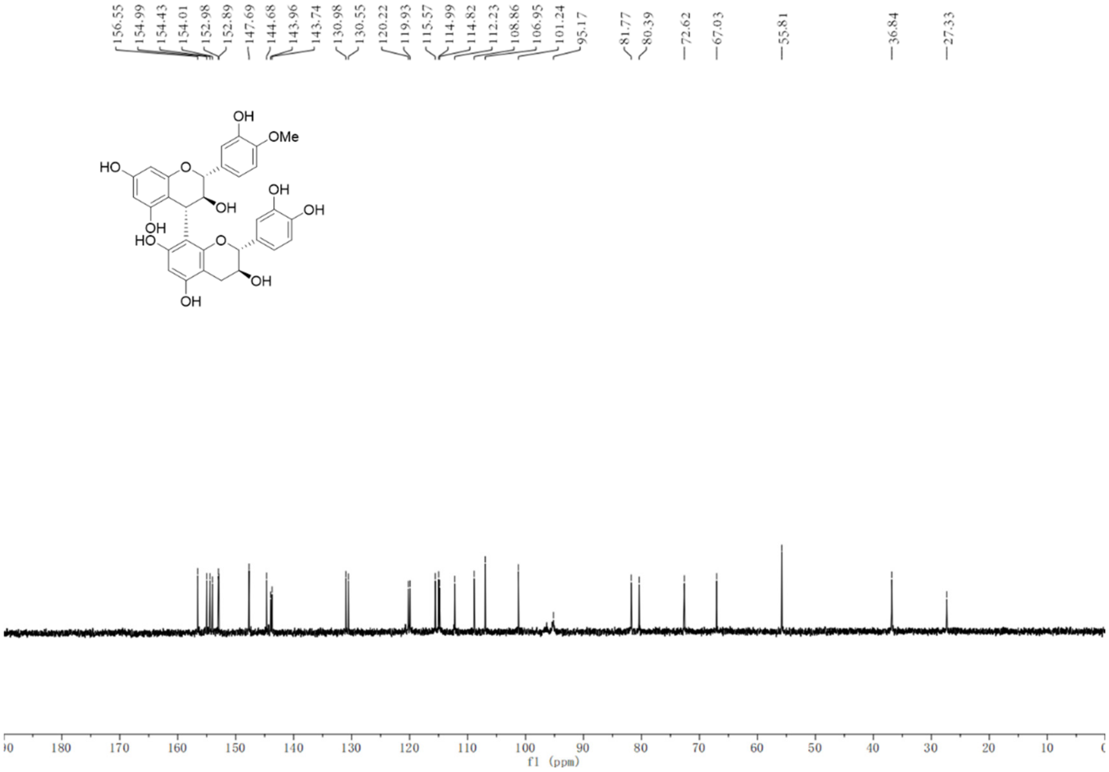

^{13}C-NMR spectrum of compound **13b** (100 MHz, D_2O)

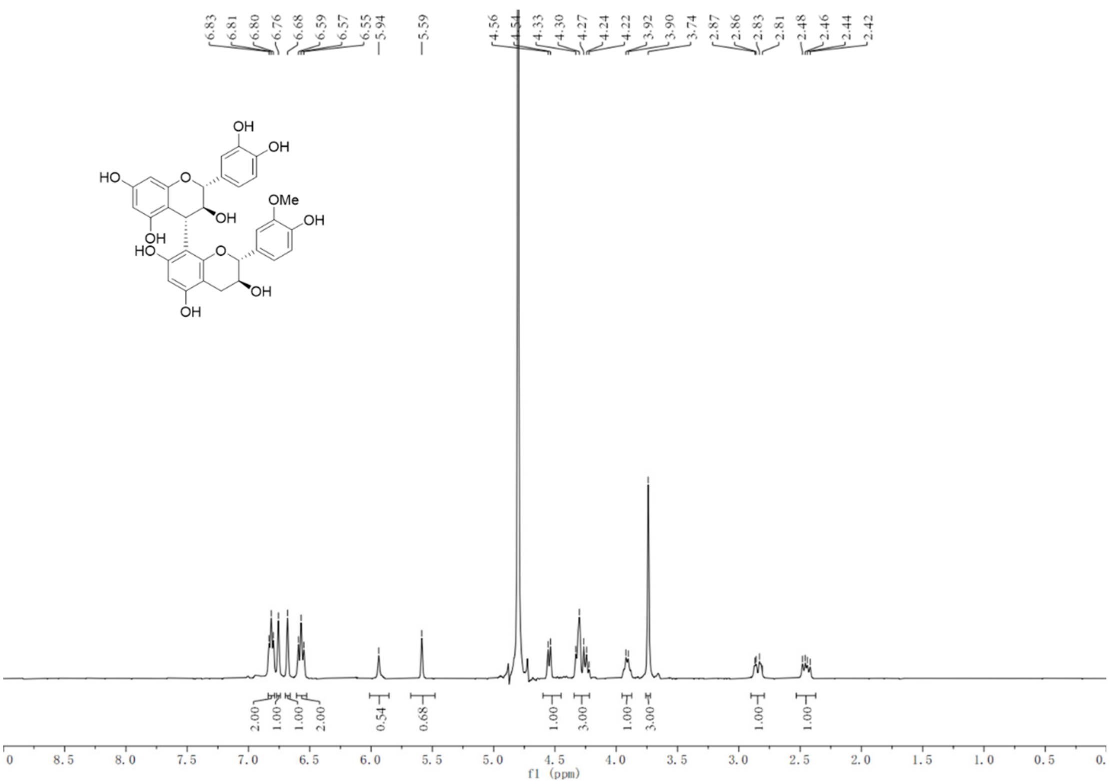

^{1}H-NMR spectrum of compound **13c** (400 MHz, D_2O)

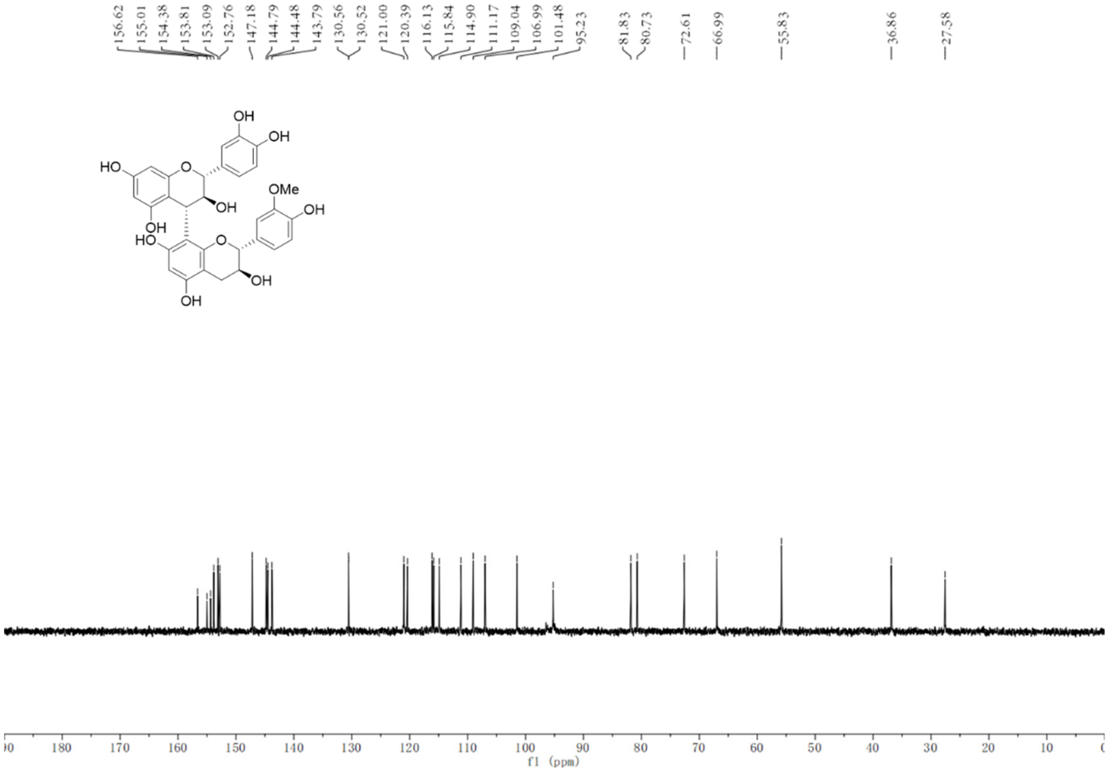

^{13}C-NMR spectrum of compound **13c** (100 MHz, D_2O)

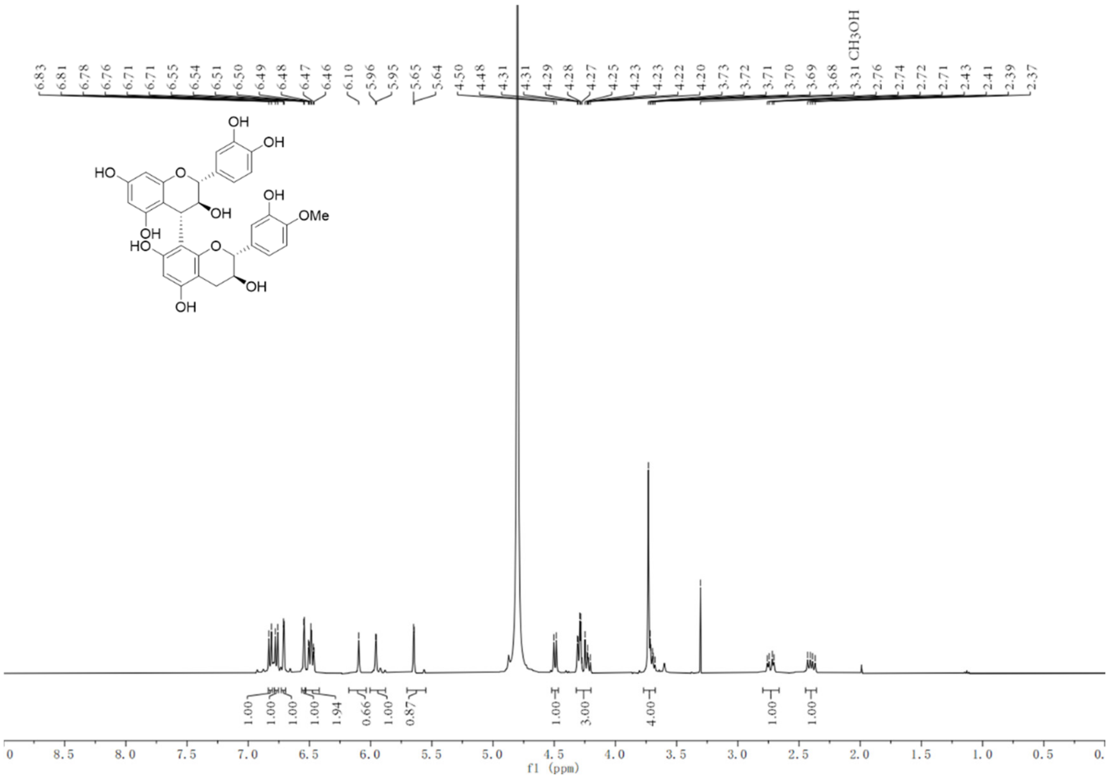

^{1}H-NMR spectrum of compound **13d** (400 MHz, D_2O)

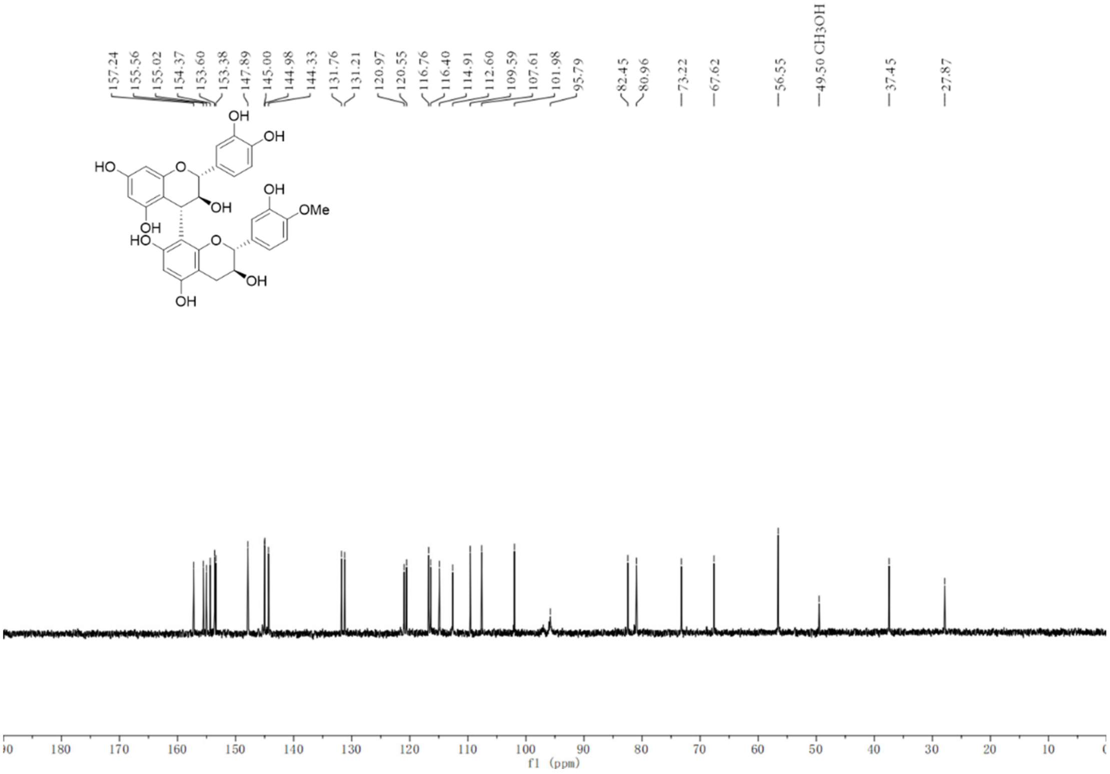

^{13}C-NMR spectrum of compound **13d** (100 MHz, D_2O)

Copies of HPLC Spectra

HPLC spectrum of procyanidin B3 (**1**).

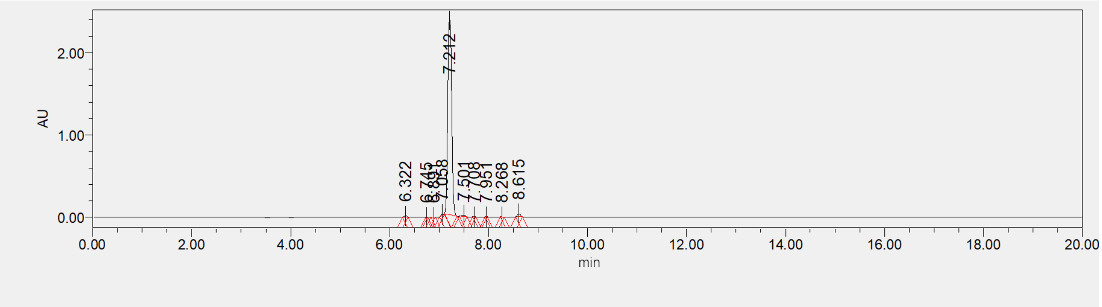

Peak	Retention time (min)	Area (%)
1	6.322	0.42
2	6.745	0.01
3	6.891	0.04
4	7.058	0.30
5	7.212	97.38
6	7.501	0.65
7	7.708	0.03
8	7.951	0.01
9	8.268	0.02
10	8.615	1.15

HPLC spectrum of compound **6b**.

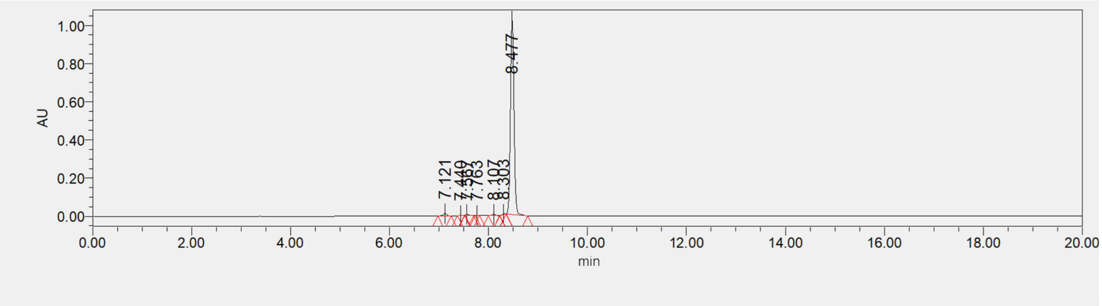

Peak	Retention time (min)	Area (%)
1	7.121	1.32
2	7.440	0.09
3	7.567	0.29
4	7.763	0.04
5	8.107	0.66
6	8.303	0.30
7	8.477	97.30

HPLC spectrum of compound **6c**.

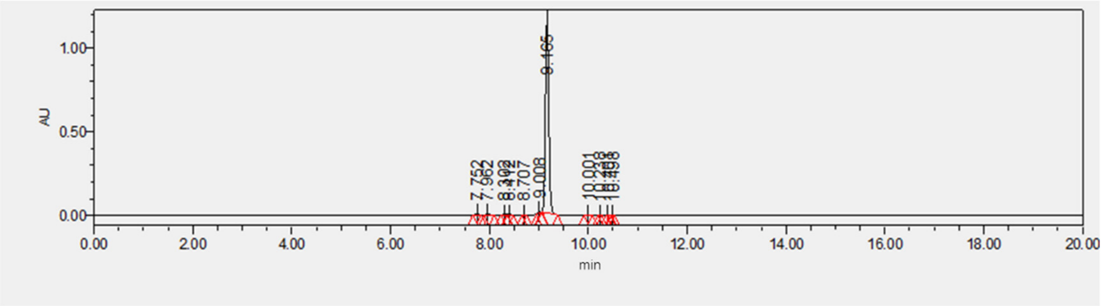

Peak	Retention time (min)	Area (%)
1	7.752	0.55
2	7.962	0.56
3	8.302	0.27
4	8.412	0.08
5	8.707	0.08
6	9.008	0.56
7	9.165	97.69
8	10.001	0.15
9	10.238	0.02
10	10.401	0.01
11	10.498	0.01

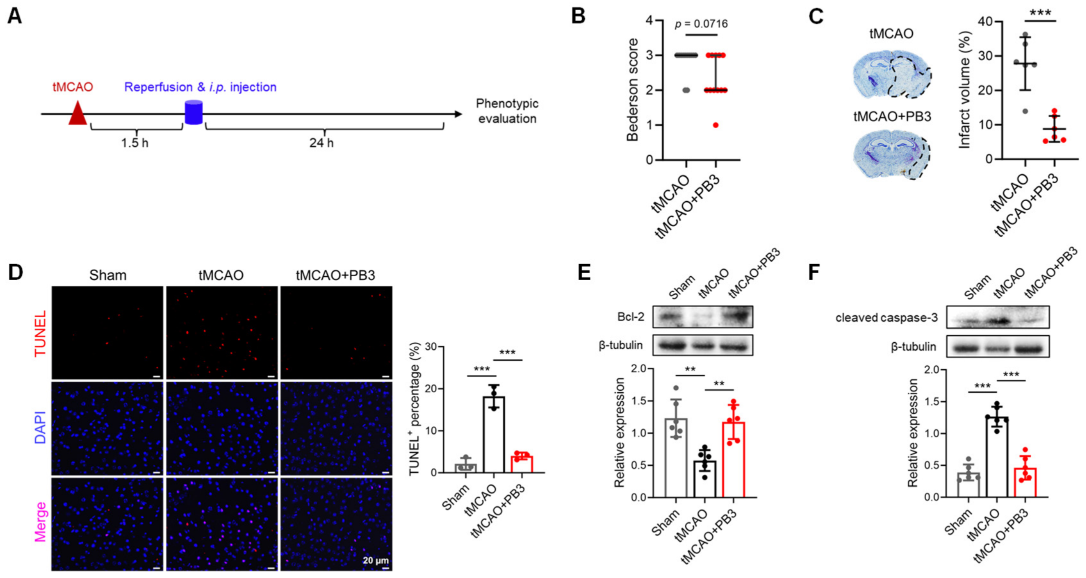

Figure S1. PB3 alleviates the neuronal damage and reduces the apoptosis level in the ischemic cortex of tMCAO mice. (A) The mouse experimental paradigm. (B) Neurological deficit score. Data are shown as median (IQR), Mann-Whitney test (n = 12). (C) Representative picture of Nissl staining, and quantification of infarct volumes. Data are expressed as mean ± S.D., $^{***}p < 0.001$, Student's *t*-test (n = 6). (D) Rate of TUNEL-positive cells in the frozen brain section of different experimental groups. Data are expressed as mean ± S.D., $^{***}p < 0.001$ vs. tMCAO, one-way ANOVA, Dunnett's multiple comparison test (n = 3). Scale bar, 20 μm. (E) Immunoblots showing the expression of Bcl-2 in different experimental groups. Data are expressed as mean ± S.D., $^{**}p < 0.01$ vs. tMCAO, one-way ANOVA, Dunnett's multiple comparison test (n = 6). (F) Immunoblots showing the expression of cleaved caspase-3 in different experimental groups. Data are expressed as mean ± S.D., $^{***}p < 0.001$ vs. tMCAO, one-way ANOVA, Dunnett's multiple comparison test (n = 6).

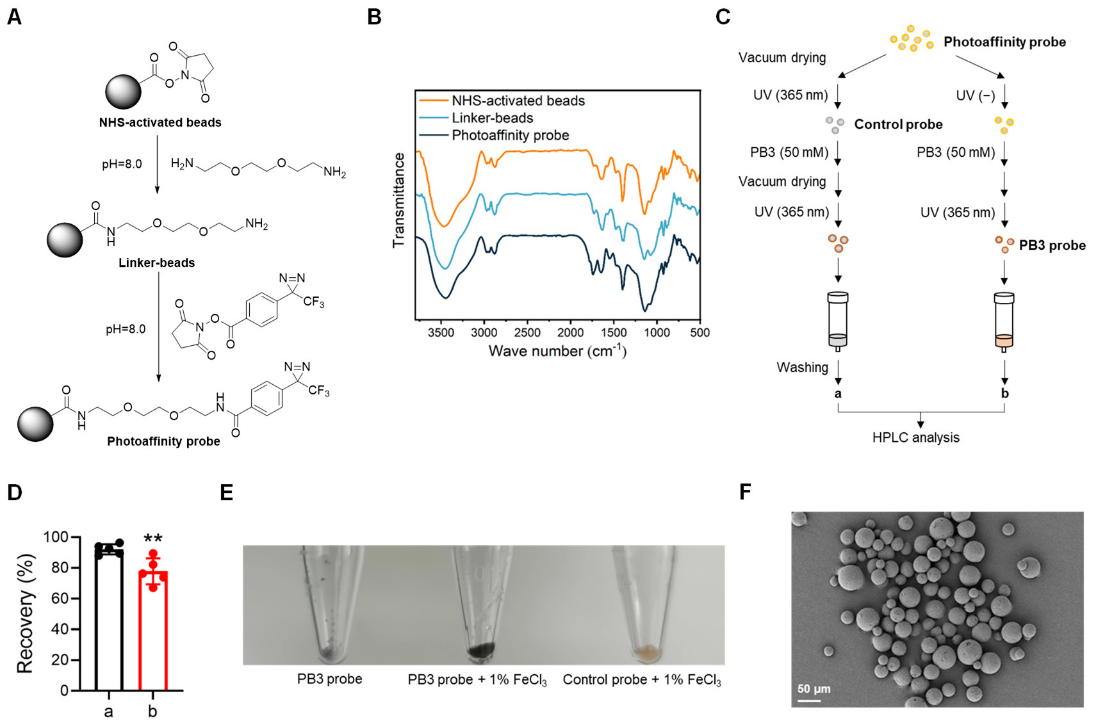

Figure S2. Preparation and identification of the PB3 probe. (A) The workflow for preparing photoaffinity probe. (B) Fourier-transform infrared spectroscopies of NHS-activated beads, linker-beads, and photoaffinity probe. The peaks shown at 1644 cm^{-1} and 1546 cm^{-1} suggested the amide bond were introduced. (C) The workflow for preparing control probe and PB3 probe. (D) Quantification of PB3 in eluent via high performance liquid chromatography. The recovery rate is equal to the ratio of the amount of PB3 in the eluent to the amount of PB3 added. Data are expressed as mean ± S.D., $^{**}p < 0.01$, Student's *t*-test (n = 5). (E) Ferric chloride staining of PB3 probe and control probe. (F) Scanning electron microscope analysis of PB3 probe. Scale bar, 50 μm.

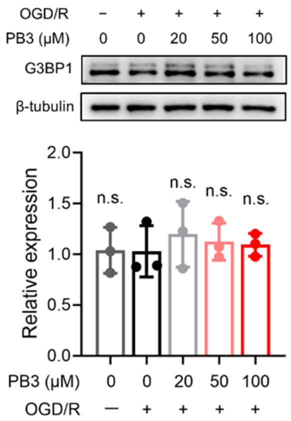

Figure S3. Immunoblots showing the expression of G3BP1 under different treatment. Data are expressed as mean ± S.D., n.s. = no significance vs. PB3 0 μM, OGD/R+, one-way ANOVA, Dunnett's multiple comparison test (n = 3).

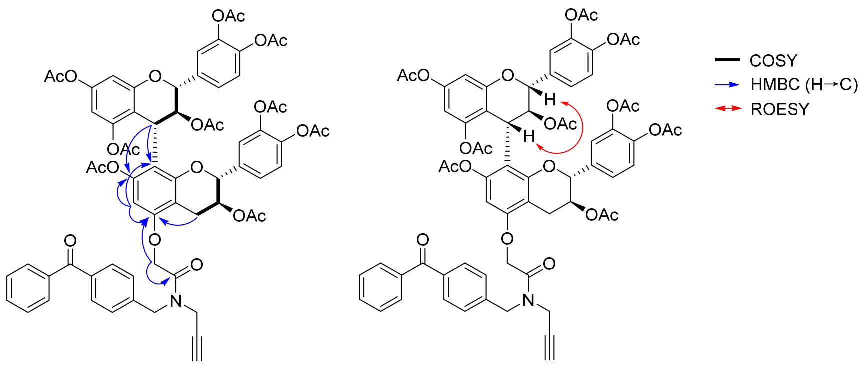

Figure S4. Key COSY, HMBC, and ROESY correlations for acetylated PB3-BP.

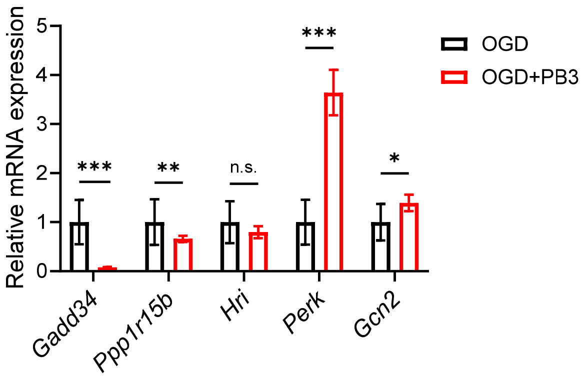

Figure S5. RT-qPCR analysis of the expression of key factors regulating SG dynamics during the degradation of SG. Data are expressed as mean ± S.D., $^{*}p < 0.05$, $^{**}p < 0.01$, $^{***}p < 0.001$, n.s. = no significance, Student's *t*-test (n = 4).

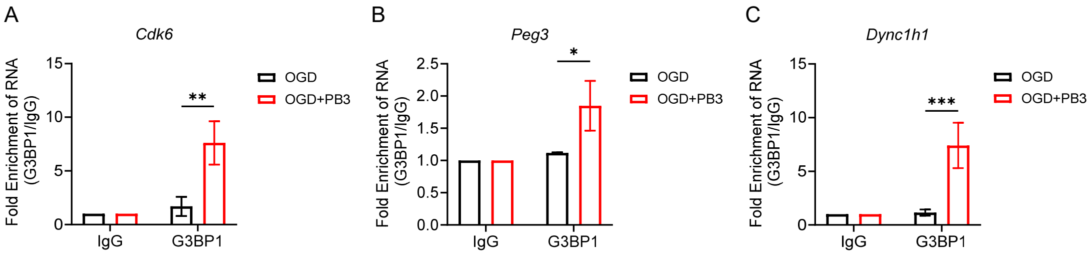

Figure S6. RT-qPCR analysis of G3BP1-bound mRNAs. Data are expressed as mean ± S.D., $^{*}p < 0.05$, $^{**}p < 0.01$, $^{***}p < 0.001$, Student's *t*-test (n = 3).

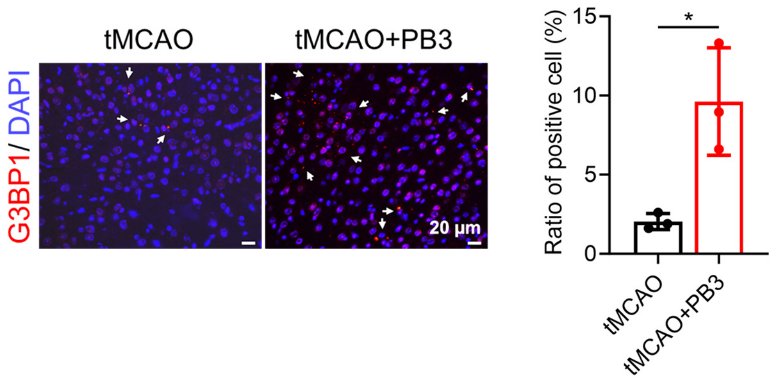

Figure S7. PB3 enhanced SG level in the ischemic cortex of tMCAO mice. Data are expressed as mean ± S.D., $^{*}p < 0.05$, Student's *t*-test (n = 3). Scale bar, 20 μm.

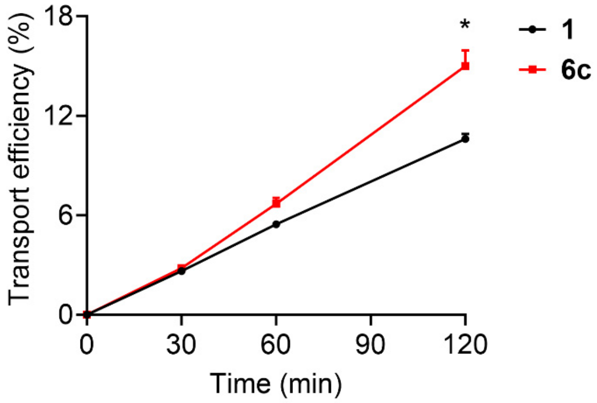

Figure S8. Transport efficiency of 1 and 6c at 100 μM in bEnd.3 cells. Transport efficiency per centage was calculated based on (compound concentrations at the basolateral side overtime)/(compound concentrations at the apical side at the zero minutes)*100. Data are expressed as mean ± S.D., **p* < 0.05, two-way ANOVA, Bonferroni's test (n = 3).

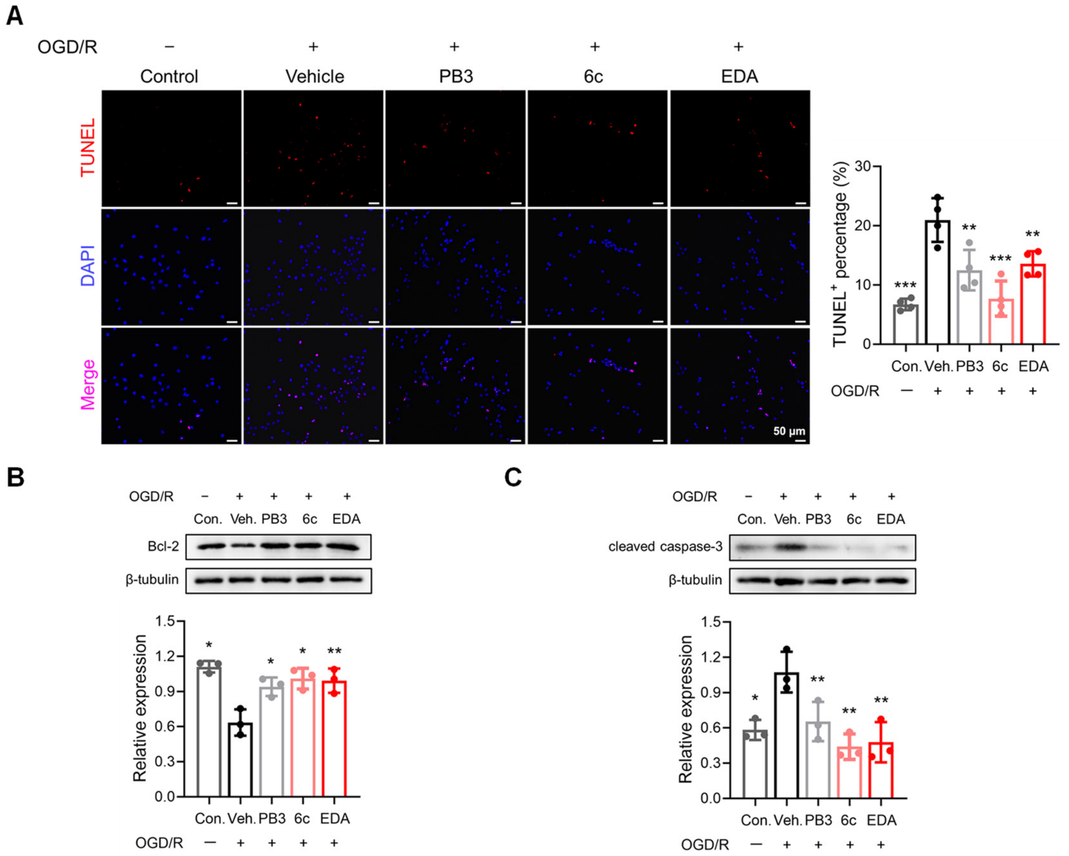

Figure S9. The anti-apoptotic effect of 6c. (A) Rate of TUNEL-positive cells in different experimental groups. Data are expressed as mean ± S.D., $^{**}p < 0.01$, $^{***}p < 0.001$ vs. Veh., one-way ANOVA, Dunnett's multiple comparison test (n = 4). Scale bar, 50 μm. (B) Immunoblots showing the expression of Bcl-2 in different experimental groups. Data are expressed as mean ± S.D., $^{*}p < 0.05$, $^{**}p < 0.01$ vs. Veh., one-way ANOVA, Dunnett's multiple comparison test (n = 3). (C) Immunoblots showing the expression of cleaved caspase-3 in different experimental groups. Data are expressed as mean ± S.D., $^{*}p < 0.05$, $^{**}p < 0.01$ vs. Veh., one-way ANOVA, Dunnett's multiple comparison test (n = 3).

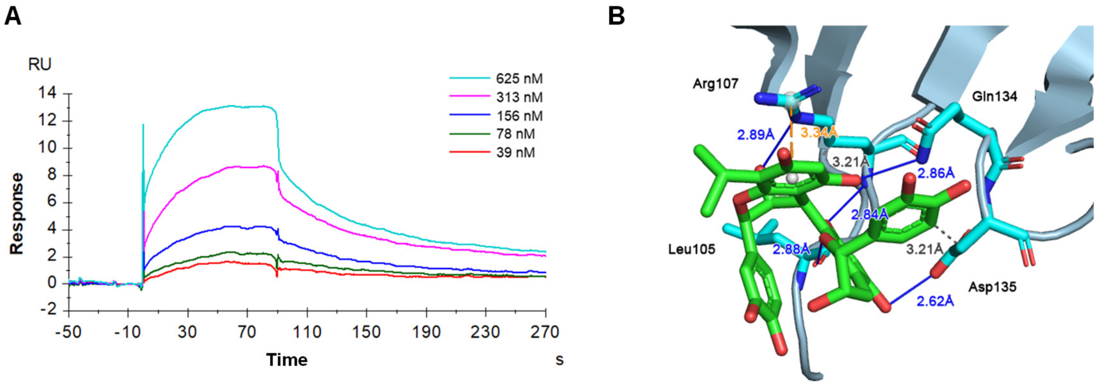

Figure S10. The interaction of 6c and G3BP1. (A) SPR analysis of 6c binding to G3BP1. (B) Docking simulation of 6c with the NTF2L domain of G3BP1. Hydrogen bonds are labeled as blue lines, hydrophobic interactions are labeled as grey dashed lines, and π-cation interaction is labeled as orange dashed lines.

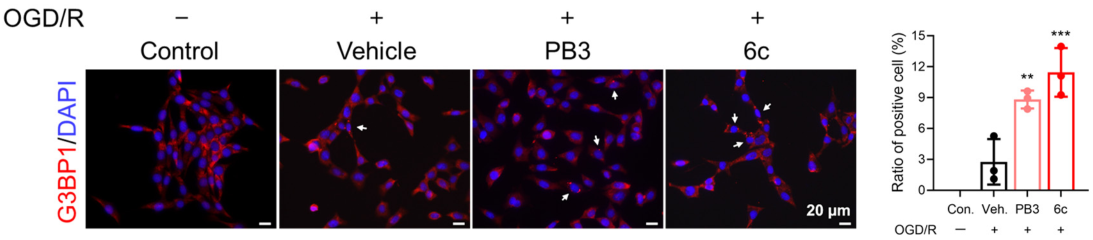

Figure S11. 6c enhanced SG level in HT22 cells under OGD/R injury. Data are expressed as mean ± S.D., $^{**}p < 0.01$, $^{***}p < 0.001$ vs. Veh., one-way ANOVA, Dunnett's multiple comparison test (n = 3). Scale bar, 20 μm.

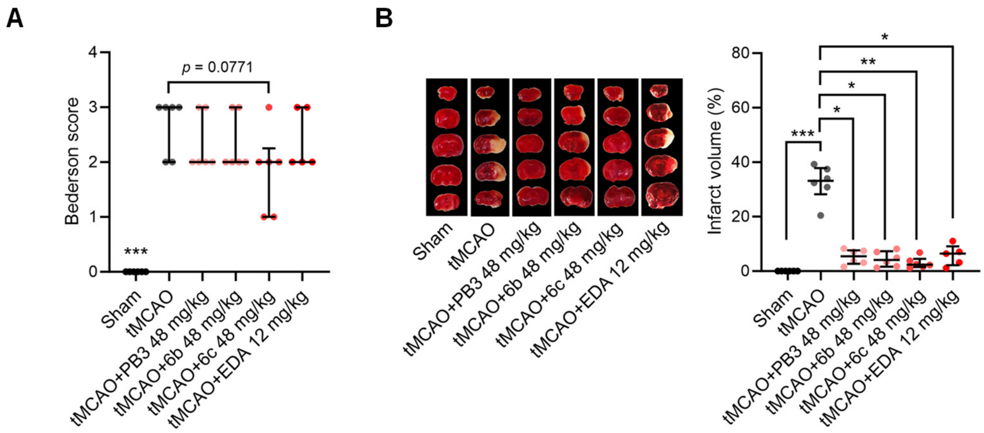

Figure S12. Effect of PB3, 6b, and 6c on infarct area and neurological function after 1 h of MCAO and 24 h of reperfusion. (A) Neurological deficit score. Data are shown as median (IQR), $^{***}p < 0.001$ vs. tMCAO, Kruskal-Wallis with Dunn's test (n = 5-6). (B) Representative picture of 2,3,5-triphenyltetrazolium chloride staining, and quantification of infarct volumes. Data are shown as median (IQR), $^{*}p < 0.05$, $^{**}p < 0.01$, $^{***}p < 0.001$, Kruskal-Wallis with Dunn's test (n = 5-6).

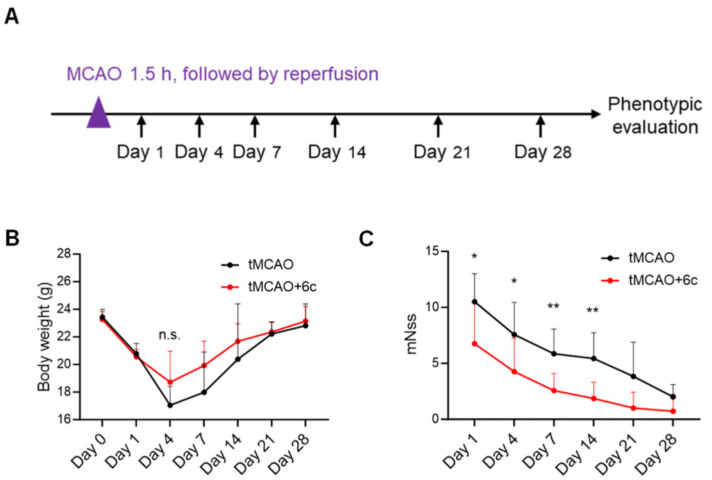

Figure S13. Effect of 6c on the long-term neurological recovery after tMCAO. (A) Treatment regime of mice, 6c was administered at a dose of 48 mg/kg daily, *i.p.* (n = 10). (B) Weight of mice during the treatment. Data are expressed as mean ± S.D., n.s. = no significance, Student's *t*-test. (C) The modified neurological severity score (mNss) of mice during the treatment. Data are expressed as mean ± S.D., $^{*}p < 0.05$, $^{**}p < 0.01$, Student's *t*-test. (D) The results of the rotarod test through measurement of time on the rod. Data are expressed as mean ± S.D., $^{*}p < 0.05$, Mann-Whitney test.

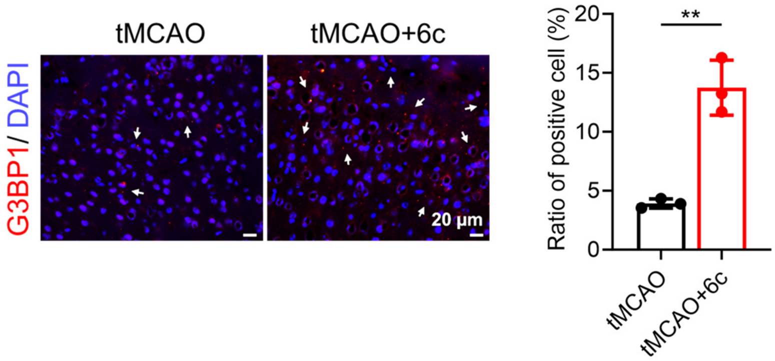

Figure S14. 6c enhanced SG level in the ischemic cortex of tMCAO mice. Data are expressed as mean ± S.D., $^{**}p < 0.01$, Student's *t*-test (n = 3). Scale bar, 20 μm.

Table S1. The list of PB3-specific binding proteins.

Gene name	Protein name	FC $_{experiment/control\ group}$	P value $_{experiment\ vs.\ control\ group}$	FC $_{experiment/\ competition\ group}$	P value $_{experiment\ vs.\ competition\ group}$
Mapk1	Mitogen-activated protein kinase 1	9.56	0.000148	0.243	0.00846
G3bp1	Ras GTPase-activating protein SH3 domain-binding protein 1	13.3	0.000188	1.33	0.0390
Gnb2l1	Small ribosomal subunit protein RACK1	3.38	0.00138	0.792	0.185
Hnrnpa0	Heterogeneous nuclear ribonucleoprotein A0	7.51	0.00370	1.70	0.0656
Slc25a4	ADP/ATP translocase 1	9.40	0.00472	0.552	0.0941
Pycr1;Pycr2	Pyrroline-5-carboxylate reductase 1;Pyrroline-5-carboxylate reductase 2	1.52	0.0114	1.20	0.710
Atp5o	ATP synthase subunit O, mitochondrial	3.32	0.0115	0.618	0.0761
Hnrnpk	Heterogeneous nuclear ribonucleoprotein K	16.4	0.0141	0.401	0.0401
Pspc1	Paraspeckle component 1	9.67	0.0159	0.615	0.235
Pdap1	28 kDa heat- and acid-stable phosphoprotein	10.8	0.0170	1.18	0.531
Cct8	T-complex protein 1 subunit theta	2.20	0.0175	0.692	0.260
Eef1a1;Eef1a2	Elongation factor 1-alpha 1; Elongation factor 1-alpha 2	14.1	0.0179	1.15	0.610
Rpsa	Small ribosomal subunit protein uS2	6.86	0.0198	0.478	0.0219
Sgpl1	Sphingosine-1-phosphate lyase 1	5.29	0.0208	0.442	0.171
Nono	Non-POU domain-containing octamer-binding protein	31.8	0.0223	1.71	0.208
Hadhb	Trifunctional enzyme subunit beta, mitochondrial	8.87	0.0239	0.503	0.0261
Ddx17	Probable ATP-dependent RNA helicase DDX17	3.99	0.0240	0.430	0.00793
Baiap2	BAR/IMD domain-containing adapter protein 2	22.7	0.0245	0.841	0.549

Ywhag	14-3-3 protein gamma	12.5	0.0247	2.11	0.209
Pkm	Pyruvate kinase PKM	11.9	0.0249	0.959	0.882
Eif2s1	Eukaryotic translation initiation factor 2 subunit 1	3.38	0.0262	0.871	0.676
Rps4x	Small ribosomal subunit protein eS4	3.14	0.0263	1.01	0.970
Prdx1	Peroxiredoxin-1	4.60	0.0269	0.663	0.123
Rps3	Small ribosomal subunit protein uS3	3.64	0.0274	0.850	0.504
Sfpq	Splicing factor, proline- and glutamine-rich	25.4	0.0278	0.389	0.00762
Acadl	Long-chain specific acyl-CoA dehydrogenase, mitochondrial	8.60	0.0284	0.778	0.418
Prdx4	Peroxiredoxin-4	4.65	0.0307	0.635	0.110
Eif4a1;Eif4a2	Eukaryotic initiation factor 4A-I; Eukaryotic initiation factor 4A-II	6.44	0.0314	1.12	0.701
Prkacb; Prkaca	cAMP-dependent protein kinase catalytic subunit beta; cAMP-dependent protein kinase catalytic subunit alpha	3.47	0.0323	0.514	0.196
Mdh2	Malate dehydrogenase, mitochondrial	8.30	0.0324	1.16	0.649
Cct4	T-complex protein 1 subunit delta	7.34	0.0368	0.524	0.0338
Dnajb1	DnaJ homolog subfamily B member 1	3.75	0.0457	0.857	0.671
Snrpb2	U2 small nuclear ribonucleoprotein B''	12.6	0.0471	0.839	0.588
Vim	Vimentin	4.30	0.0478	0.315	0.197
Ldha	L-lactate dehydrogenase A chain	11.8	0.0483	1.09	0.817
Anxa2	Annexin A2	4.20	0.0496	1.86	0.166
Hspa5	Transmembrane protein 132A	2.35	0.0498	0.646	0.298

Table S2. MMGBSA analysis of PB3 binding to wild type and mutants of the G3BP1 NTF2L domain.

	**MMGBSA Free energy (kcal/mol)**
**wild type**	-22.11 ± 1.94
**L105A**	-18.47 ± 1.23
**R107A**	-17.24 ± 1.91
**Q134A**	-19.28 ± 1.25
**D135A**	-16.61 ± 1.77

Table S3. siRNA used for transfection.

siRNA	Sequence (5' to 3')
siG3BP1-1	CCGACAUUCAAGAGGACAATT
	UUGUCCUCUUGAAUGUCGGTT
siG3BP1-2	CCUGUUCAGAAGGUCCUUATT
	UAAGGACCUUCUGAACAGGTT

Table S4. Primers used for RT-qPCR analysis.

Gene name	Forward primer sequence (5' to 3')	Reverse primer sequence (5' to 3')
Gadd34	GAGGAGAAGCTGGGTCCCTA	GGTTTCACTGGGAAGGGGAG
Ppp1r15b	CAAGTTACAGCGCCCTGAGA	GGAGTCCCTCCTCTAGCCAA
Hri	CACGGACACACACATCCAGA	AACCTTGGCATCGTACTGGG
Perk	TGGATGCCGAGAATGATGGG	ATCCCAACACACGCTCAGAG
Gcn2	CTACACAGCAACTCCGTGGT	ACGAACTCGAGCTTGCTCAA
Cdk6	GCATCGTGATCTGAAACCGC	ATAGCTGGACTGGAGCAGGA
Peg3	TCAGTGATCCAGGAGAGGGG	GCTTTTATGCTCATGGCCCG
Dync1h1	AGAACCTGAACTCCGTGCTG	GCGTCGCGTATTTCAAGTCC

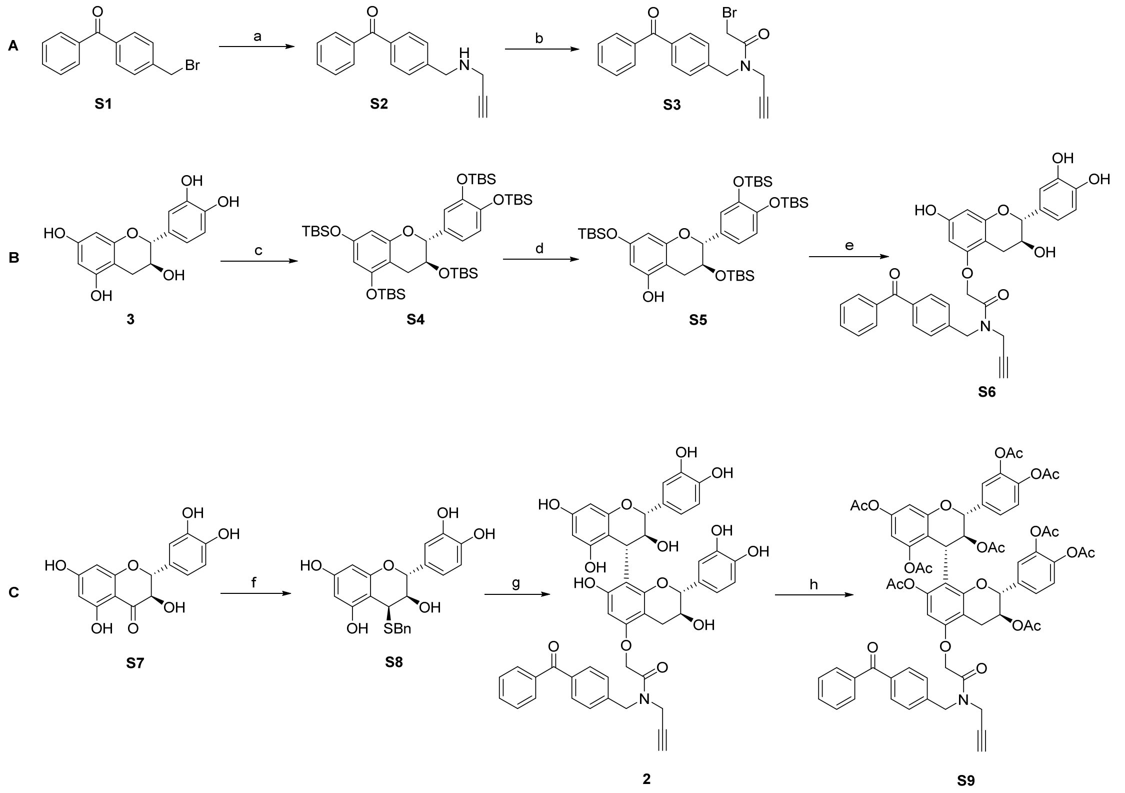

Scheme S1. Synthesis of PB3-BP. Reagents and conditions: (a) propargylamine, K_2CO_3, DCM/THF, r.t., overnight, 72%; (b) bromoacetyl bromide, $NaHCO_3$, DCM, -10°C to r.t., overnight, 72%; (c) imidazole, *t*-butyldimethylchlorosilane, DMF, r.t., 18 h, 97%; (d) trifluoroacetic acid, DCM, 0°C to r.t., 18 h, 10%; (e) 1) S3, K_2CO_3, acetone, r.t., 18 h; 2) acetic acid, tetrabutylammonium fluoride, THF, 45 min, 0°C; 3) FA, THF/H_2O, r.t., overnight, 21%; (f) $NaBH_4$, BnSH, acetic acid, EtOH, 0°C to r.t., overnight, 47%; (g) S6, $AgBF_4$, THF, 0°C, 30 min, 32%; (h) DMAP, pyridine, Ac_2O, r.t., 90 min, 52%. r.t. = room temperature.

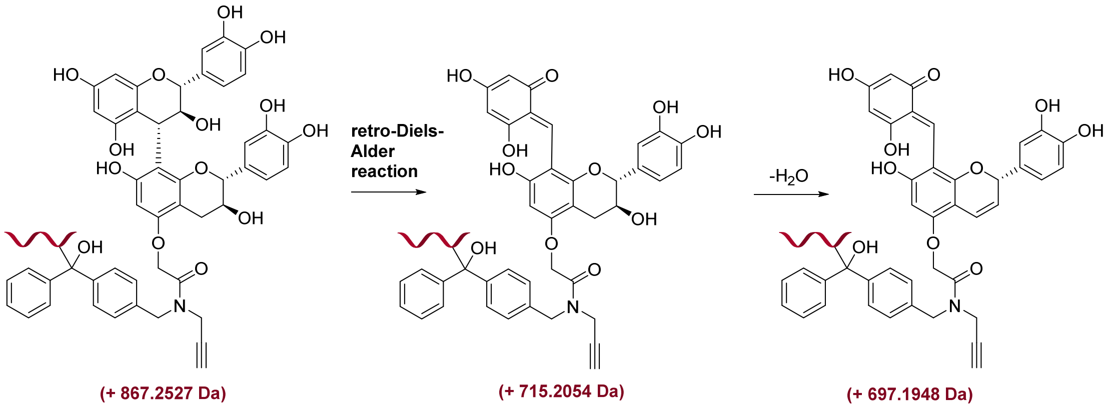

Scheme S2. Fragmentation pathway of PB3-BP modified peptides.
